# Functional diversity of snakes is explained by the landscape composition at multiple areas of influence

**DOI:** 10.1002/ece3.10352

**Published:** 2023-07-26

**Authors:** Mónica Rincón‐Aranguri, Felipe A. Toro‐Cardona, Sandra P. Galeano, Lilia Roa‐Fuentes, Nicolás Urbina‐Cardona

**Affiliations:** ^1^ Departamento de Ecología y Territorio, Facultad de Estudios Ambientales y Rurales Pontificia Universidad Javeriana Bogotá Colombia; ^2^ Grupo Herpetológico de Antioquia, Instituto de Biología Universidad de Antioquia Medellín Colombia; ^3^ Grupo de Ecología y Evolución de Vertebrados, Instituto de Biología Universidad de Antioquia Medellín Colombia; ^4^ Centro de Colecciones y Gestión de Especies Instituto de Investigación de Recursos Biológicos Alexander von Humboldt Villa de Leyva Colombia

**Keywords:** functional redundancy, land use/land cover change, reptiles, roadkill, scale of effect, trait probability density

## Abstract

Roadkill and landscape composition affect snakes at different spatial scales, depending on the functional trait value of the species, which is reflected in the functional diversity indices at the assemblage level. This study evaluated the effect of roads and landscape composition on snakes' functional diversity at different areas of influence (250, 500, 1000, and 2000 m buffer areas). We compared roadkill snake species with those assemblages inhabiting the adjacent vegetation in the Orinoco region, Colombia. We surveyed snakes using transects on the road and adjacent areas on 13 landscapes along the road. We evaluated the effect of 16 landscape metrics at six land cover classes on the snake's functional diversity at four different areas of influence (from 250 to 2000 m around the sampled sites). The functional redundancy index was higher for roadkill species, suggesting that roads eliminate species that play similar roles in the assemblage and ecosystem processes. Likewise, the low values of functional redundancy in the adjacent vegetation call attention to the fact that each species surviving in this transformed landscape has a crucial active role in ecosystem processes in snake assemblages. For roadkill snakes, forest metrics explained changes in functional richness and functional evenness at a 250 m area of influence. In comparison, transient crop and pasture metrics explained changes in functional evenness and divergence at 2000 m. For snakes inhabiting the adjacent vegetation, the cohesion of pasture explained changes in functional richness at 250 m, and forest metrics explained changes in functional redundancy and evenness at 2000 m. Anthropogenic landscape transformation may have a greater effect on snake functional diversity at local scales than roadkill. In savanna ecosystems, the presence of native forest at 2000 m radius around roads promotes the conservation of snake assemblages. However, within a 250 m radius, the risk of snake roadkill increases when the road borders native forest. Therefore, it is necessary to implement wildlife crossing in these sections of the road.

## Graphical abstract

This study investigated the impact of roads and landscape composition on the functional diversity of snakes in the Orinoco region of Colombia. The results suggest that landscape composition affects the functional diversity of both road‐killed snakes and those inhabiting the adjacent vegetation within an area of influence ranging from 250 to 2000 m. The functional redundancy index was higher for roadkill species, indicating that in the adjacent vegetation, each species plays a distinct role in ecosystem processes.

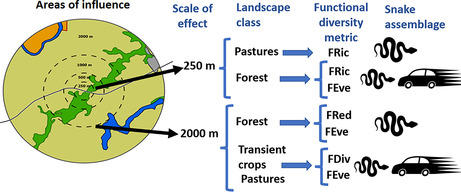

## Resumen

El atropellamiento en carreteras y la composición del paisaje afectan a las serpientes a diferentes escalas espaciales, dependiendo del valor del rasgo funcional de las especies, que se refleja en los índices de diversidad funcional a nivel de ensamblaje. Este estudio evaluó el efecto de las carreteras y la composición del paisaje en la diversidad funcional de las serpientes en diferentes áreas de influencia (250, 500, 1000 y 2000 m áreas de amortiguamiento). Comparamos las especies de serpientes atropelladas con los ensamblajes que habitan la vegetación adyacente en la región del Orinoco, Colombia. Muestreamos serpientes usando transectos en la carretera y áreas adyacentes en 13 paisajes a lo largo de una carretera. Evaluamos el efecto de 16 métricas de paisaje en seis clases de cobertura del suelo sobre la diversidad funcional de la serpiente en cuatro áreas diferentes de influencia (de 250 a 2000 m alrededor de los sitios muestreados). El índice de redundancia funcional fue mayor para las especies atropelladas, lo que sugiere que las carreteras eliminan especies que desempeñan funciones similares en los procesos de ensamblaje y ecosistema. Del mismo modo, los bajos valores de redundancia funcional en la vegetación adyacente llaman la atención sobre el hecho de que cada especie que sobrevive en este paisaje transformado tiene un papel crucial en los procesos ecosistémicos y dentro de los ensamblajes de serpientes. Para las serpientes atropelladas, las métricas de cobertura de bosque explicaron los cambios en la riqueza y la uniformidad funcional en un área de influencia de 250 m. En comparación, las métricas de cultivos transitorios y pastos explicaron los cambios en la uniformidad y divergencia funcional a 2000 m. Para las serpientes que habitan la vegetación adyacente, la cohesión de los pastizales explicó los cambios en la riqueza funcional a 250 m, y las métricas de cobertura de bosque explicaron los cambios en la redundancia y la uniformidad funcional a 2000 m. La transformación antropogénica del paisaje puede tener un mayor efecto en la diversidad funcional de las serpientes a escalas locales que los atropellos. En ecosistemas de sabana, la presencia de bosque nativo en un radio de 2000 m alrededor de las carreteras favorece la conservación de los ensamblajes de serpientes. Sin embargo, en un radio de 250 m se aumenta el riesgo de atropellamiento de serpientes cuando la carretera limita con bosque nativo, por lo que es necesario implementar pasos de fauna en estos tramos.

## 1 INTRODUCTION

Road development is one of the main drivers of land use and cover change, leading to habitat fragmentation, degradation, and loss of native species (Dixon et al., 2022; Moore et al., 2023). This anthropogenic drive also increases fauna collision probability, which varies depending on the value of the functional traits related to the species' vulnerability to roadkill (González‐Suárez et al., 2018; Rytwinski & Fahrig, 2012). Thus, the change in the composition and dominance of native species in the assemblages due to the roadkill of species with greater vulnerability is reflected in changes not only in taxonomic diversity (Coffin, 2007) but also in functional diversity, which is defined as the value and range of functional traits among the organisms, and how these traits are linked to the ecological functions and interactions of these species with their surrounding environment (Carmona et al., 2019; Mason et al., 2005). However, the composition and configuration of the landscape also mediate the effect that roads can have on assemblages, depending on the degree of complementation or supplementation the native species make in the landscape (Jackson & Fahrig, 2015; Moraga et al., 2019).

Variability in mortality rates of roadkill species has been described across life‐history traits such as ecological habits, behaviors, movement patterns, and reproductive times, among others (González‐Suárez et al., 2018; Medrano‐Vizcaíno et al., 2022). Some animal species may be more vulnerable to vehicle collisions and road mortality than others (Andrews et al., 2015), influencing changes in the functional diversity of assemblages (Trimble & Van Aarde, 2014). Road mortality is related to reptiles' ectothermic condition because the roads are heat sources (D'Amico et al., 2015). Snakes are some of the vertebrates most affected by roads (Mccardle & Fontenot, 2016; Sosa & Schalk, 2016), and their mortality is influenced by the wide range of life‐history traits of species within an assemblage, as well as their habitat preferences, daily and seasonal activity patterns, vagility, and foraging strategies (Bonnet et al., 1999).

Foraging strategy is an important trait that defines how a snake searches for and captures its prey, which can be active foraging (which requires a permanent expenditure of large amounts of energy in the exploration of the environment and the pursuit of prey), or sit and wait (in which the snake ambushes the prey after waiting for a long time without moving within a microhabitat) (Lillywhite, 2014). In that sense, it is expected to find a greater number of roadkill species whose foraging strategy is to actively search for prey because they have greater dispersal throughout the habitat. Also, the temporal dynamics of foraging reflects the circadian rhythm of the species, which is determined by melatonin levels in the body, and in turn affects internal body functions related to reproduction and exploration of light and temperature gradients in the environment (Lillywhite, 2014). It is expected for nocturnal species to be run over more frequently than diurnal while acquiring heat from asphalt (Das et al., 2007), one of the few surfaces that can maintain heat, which makes it an ecological trap (Mccardle & Fontenot, 2016). Finally, habitat preference may determine species' vulnerability to anthropogenic changes in natural cover. For example, fossorial species may be less affected by human constructions and infrastructure (Lillywhite, 2014) and inhabit pastures, while arboreal species tend to habit on the edges and interior of remnant native forests (Urbina‐Cardona et al., 2008). In this sense, terrestrial species are more likely to be victims of roadkill compared to species with different habitat preference. Rincón‐Aranguri et al. (2019), evaluated how these life‐history traits were related to landscape composition to explain road mortality. However, such an approach fails to clarify how the landscape composition explains roadkill and its effect on functional diversity. In a complementary way, considering fixed traits (such as life history) and field‐measured traits that vary within and between populations (Shipley et al., 2016) can give a helpful insight into how the roadkill events structure snake assemblages.

Measuring the functional traits of species living in the adjacent vegetation in contrast with road‐killed can be vital to understanding the link between the assembly structure and road risk factors (Lawing et al., 2012). Trait selection is a core topic in understanding the species´ response to environmental conditions (Shipley et al., 2016), and for snakes, it has been reported that the body size is sensitive to anthropogenic landscape transformation (Doherty et al., 2020; Pfeifer et al., 2017; Todd et al., 2017) and the pattern of road‐killed snakes (Mccardle & Fontenot, 2016; Sosa & Schalk, 2016) because of their association with vagility and home range (Andrews & Gibbons, 2005; Bonnet et al., 1999; Doherty et al., 2019). Total size and tail size in snakes are related to a kind of locomotion, habit (i.e., fossorial vs. arboreal), mate acquisition, and habitat preferences (Jayne, 1986; Lawing et al., 2012) and represent the risk of roadkill due to their influence on the shape and velocity of movement over the asphalt on the road (Andrews & Gibbons, 2005). In addition, both traits are good proxies for the matter and energy that everyone contributes to the ecosystem as prey, as a carcass, or as a top predator (Cortés‐Gomez et al., 2015; De Miranda, 2017; Lillywhite & Henderson, 1993). Then, larger body size increases the risk of mortality in roadkill events (Roe et al., 2006), which affects the population's fitness (Barron & Andraso, 2001) and predator–prey dynamics (King et al., 2002).

The Colombian Orinoco exhibits a highly heterogeneous landscape with natural regions such as savanna, Moriche palm fragments (*Mauritia flexuosa*), and gallery forests imbibed within anthropogenic land use areas such as production systems and pastures (Rangel‐Ch et al., 1995). Since 1970 this landscape has been transformed by introducing African grasses for cattle ranching (Etter et al., 2010), African palm plantations, and rice crops (Romero‐Ruiz et al., 2011). This transformation has exacerbated the development and use of roads, with a road network improvement and expansion of approximately 25% in the last 8 years (DNP, 2014, 2015). The Meta department, within the Orinoco region, harbors 47 of the 324 snake species recorded for Colombia (Ramírez‐Villalba et al., 2014; Rincón‐Aranguri et al., 2022; Uetz et al., 2021), with an estimated mortality by vehicle collision of about 30,129 individuals per year on 913 km of pavement roads (Lynch, 2012; Rincón‐Aranguri et al., 2019), posing a great challenge for the conservation of snake assemblages in this highly dynamic landscape.

In this study, we used the snake's body and tail size intraspecific variation to explore the range of values and the size of the variation at the assemblage level that is lost due to the runover. We selected the trait probability density as an analytical approach that considers all field‐observed variations on species abundance and functional trait value variation to unbiasedly calculate functional diversity indices (Carmona et al., 2019). Based on the distribution probabilities of both size traits values, it is possible to calculate the size of the functional volume occupied by each species (Functional richness per species or FRic_sp), the functional volume of all species that make up the assemblage (Functional richness or FRic), the equitability in the distribution of the abundances of the different values of the traits (Functional evenness or FEve), the distribution of abundances within the functional volume occupied by each assemblage (Functional divergence or FDiv) (Mason et al., 2005) and the volume of trait space overlap between species in a assemblage (Functional redundancy or FRed; Carmona et al., 2019).

We aimed to identify the variation in functional diversity between road‐killed species and those assemblages inhabiting the adjacent vegetation, as a function of changes in landscape composition at 13 surveyed landscapes on the road between Villavicencio city and Puerto López town at the Orinoco region of Colombia. Our specific goals were to: (1) describe the variation in five functional traits (three fixed traits: foraging strategy, temporal dynamic foraging, and habitat preferences; and two traits measured in the field: body and tail length) and the functional richness index per species (FRic_sp) of roadkill snakes and those found alive in the adjacent vegetation; (2) evaluate changes in the functional diversity indexes measured at the assemblage level between roadkill snakes and those inhabiting adjacent vegetation; (3) spatially represent changes on functional diversity indexes of snakes from the road and the adjacent vegetation cover for the 13 landscapes surveyed; and (4) determine the effect of landscape composition on changes in the functional diversity of roadkill snakes assemblages and those found alive inhabiting adjacent vegetation, at four buffer areas of influence (scale of effect; Jackson & Fahrig, 2015). We expected higher values of functional diversity for snakes inhabiting the adjacent vegetation than for roadkill snakes, suggesting that roads may filter a small portion of the regional pool of snake species that would be functionally similar (i.e., have high functional redundancy sensu Adams et al., 2022). This diversity of roadkill snakes would be explained by metrics related to the extent and connectivity of native forests, mainly within 250 and 500 m of the sampled site. This is because species inhabiting areas with high canopy cover tend to seek open areas to thermoregulate, being run over when exiting onto roads (Andrews et al., 2015). In contrast, the functional diversity of snakes inhabiting the adjacent vegetation was expected to be explained by metrics related to the extent of the different natural and anthropogenic classes (at 1000 and 2000 m around the sampled site) due to the ability of some species to perform landscape complementation and supplementation when land cover types offer refuges and hedgerows for their dispersal (Graitson et al., 2020; Lecq et al., 2017).

## 2 METHODS

### 2.1 Study site

The study was carried out in an urban–rural area in the Llanos Orientales of the Colombian Orinoco, at the Department of Meta, Colombia, on a paved road (width of 10 m) between Villavicencio city (4°06′41″ N and 73°36′16″ W) and Puerto Lopez town (4°04′51″ N and 72°58′11″ W) (see Appendix S1). The region has a unimodal bi‐seasonal rainfall regime, with an annual mean precipitation of 2414 mm and a mean temperature of 25.7°C (Instituto de Hidrología y Ministerio de Ambiente, 2005). The road is between 192 and 400 masl and has an extension of 79.7 km that travels from the foothills of the Eastern Cordillera to the Orinoco natural savannas. A suburban mosaic settlement dominates the current landscape, added to natural gallery forest remnants, natural savannas with linear palm elements (Morichales), exotic pastures for extensive ranching, and agricultural systems (Romero‐Ruiz et al., 2011).

### 2.2 Snakes sampling

We selected 13 landscapes along the road based on the inspection of satellite images. The landscapes were located at a minimum distance of 1 km (considering Neotropical reptile dispersal; Mendenhall et al., 2014), and each one presented different levels of traffic and extension of the different classes or types of land cover (see Appendix S4 in Rincón‐Aranguri et al., 2019). At each landscape, we surveyed snakes using a 1 km permanent transect along the road and 10 transects of 100 m located randomly within the vegetation cover types and parallel to the road (at a minimum distance of 10 m and a maximum distance of 800 m from the highway). The same two people sampled the transects using the visual encounter survey method from ground level up to 2 m into the understory (Urbina‐Cardona et al., 2008). We conducted six 13‐day surveys from March 2017 to January 2018. This method made it possible to sample the road and adjacent vegetation in the same landscape on the same day. Surveys were conducted for 9 h each day (1 h on the road and 8 h in the adjacent vegetation), completing a total sampling effort of 1404 person‐hour (from which 156 h corresponded to road transects and 1248 h to the adjacent vegetation transects). Although sampling was carried out in a standardized manner, covering the same distance on the road and at the adjacent vegetation for each landscape, it took less time to find the carcasses on the road than it did to detect the snakes inhabiting the adjacent vegetation. Corpses of roadkill snakes were collected, then fixed in 10% formalin and deposited at the Museo de Herpetología Universidad de Antioquia (Resolution 0524 of 2014 “framework permit for the collection of specimens of wild species of biological diversity for non‐commercial scientific research” National Environmental Licensing Authority—Ministry of Environment and Sustainable Development of Colombia). All specimens were identified to the lowest taxonomic level possible using herpetological keys (Köhler, 2003; Savage, 2002). Whenever it was not possible to identify individuals found in the adjacent vegetation, they were sacrificed with an anesthetic injection (protocol approved by the Committee for Ethical Experimentation with Animals of the Universidad de Antioquia; Act No. 106 of October 13, 2015) and deposited at the Museo de Herpetología Universidad de Antioquia.

### 2.3 Functional traits and diversity

For each snake found, we measured in the field two continuous morphological traits (Total length, Tail length) following a standard protocol (Rivas et al., 2008). When it was not possible to obtain a value for any of these traits from field data (i.e., because corpses were too damaged, tailless, or individuals escaped), we used the mean value reported in the literature for that species (five out of 119 total individuals found). Both continue traits were used to calculate the functional richness (FRic), functional evenness (FEve), functional divergence (FDiv), and functional redundancy (FRed) as described below.

Patterns of species richness were examined considering the three categorical functional traits (foraging strategy, temporal dynamic foraging, and habitat preferences) obtained from the scientific literature (Rincón‐Aranguri et al., 2019). The number of species was determined for each state or category of each functional trait by comparing the assemblages of roadkill snakes and snakes inhabiting the adjacent vegetation.

### 2.4 Characterization of landscape composition

We used a vectorial layer from a classified image with a 1:25,000 spatial scale from the GEF‐BID project (2016) to evaluate landscape structure and composition on each of the 13 landscapes along the road. Because the vectorial layer comprised Landsat eight images from 2014, and our snake field surveys were conducted from 2017 to 2018, we validated the land use and land cover during our field trips. We modified the land classes manually (adding and removing polygons to update the vegetation type in 2014). Finally, we grouped the vegetation cover types into six land classes: Native Forest, pastures, transitory crops, permanent crops, bodies of water, and urban infrastructure.

For each of the 13 landscapes, we converted the polygons to raster layers with a 6 m × 6 m pixel size according to the scale (1:25,000) following the user manual of FRAGSTATS 3.4. software (McGarigal & Marks, 1995). Each landscape was defined as “a spatial area with a diameter exceeding the dispersal distance of the species of interest” (Driscoll et al., 2013). We selected four buffer distances for each of the 13 landscapes, 2000 m (exceeding the dispersal distance of the most vagile species on the assemblage), 1000, 500, and 250 m. We defined the largest buffer for the landscape analysis considering the highest dispersion reported for some species in the region (Jackson & Fahrig, 2015), of which *Eunectes murinus* has an average distance per week of 1500 m (Rivas et al., 2016). We clipped the previously classified raster layer at all buffers for the 13 surveyed landscapes. We used FRAGSTATS 3.4. software (McGarigal & Marks, 1995) to calculate 16 metrics (Table 1) for each land class at each of analysis buffers.

**TABLE 1:** Landscape metrics calculated on FRAGSTATS 3.4. software for each of six land use classes (native forest, pastures, transitory crops, permanent crops, bodies of water, and urban infrastructure) measured at four buffers (2000, 1000, 500, and 250 m) for each of the 13 surveyed landscapes.

Metric number	Name of metric	Code of the metric
1	Total area	CA
2	Percentage of landscape	PLANO
3	Number of patches	NP
4	Patch density	PD
5	Mean_Patch area	AREA_MN
6	Standard deviation_Patch area	AREA_SD
7	Mean_Radius of Gyration of Patch area	GYRATE_MN
8	Standard deviation_Radius of Gyration of Patch area	GYRATE_SD
9	Mean_Perimeter – Area ratio of patch	PARA_MN
10	Standard deviation_Perimeter – Area ratio of patch	PARA_SD
11	Mean_Contiguity index of patch	CONTIG_MN
12	Standard deviation_Contiguity index of patch	CONTIG_SD
13	Mean_Euclidean nearest neighbor distance	ENN_MN
14	Standard deviation_Euclidean nearest neighbor distance	ENN_SD
15	Patch Cohesion index	COHESION
16	Effective mesh size	MESH

### 2.5 Data analyses

We calculated the trait probability density functions (TPD) of Total Length and Tail Length of each species found on the road and at the adjacent vegetation. The TPD calculation considers the variability within species before quantifying the distribution of the functional traits of species within the assemblage. Some trait values are more probable and reflect the probabilistic nature of functional diversity (Carmona et al., 2019). The TPD avoids using a single value to represent the trait values of a species and excludes the positive correlation with the number of species richness discussed in the functional ecology framework (Karadimou et al., 2016). We calculated the functional richness (FRic_sp) index per species on the road and the adjacent vegetation. To quantify species occupying peripheral or core positions in each single trait axis, we subtracted the mean trait value for the entire assemblage from the mean trait value for each species. The displacement of a species from the overall assemblage trait distribution was calculated. We described the variation in three fixed functional traits (foraging strategy, temporal dynamic foraging, and habitat preferences) through bar charts showing the richness by trait attribute of roadkill species and those that inhabit the adjacent vegetation. Then, together with the two functional traits measured in the field, the values of the five functional traits were standardized, and a Gower distance matrix was calculated. With this matrix, bootstrap averages were calculated, to estimate the mean functional space on a metric MDS (multidimensional scaling). This technique is used to evaluate the accuracy of the MDS results by sampling the data multiple times and computing the MDS for each sample. The averages per group (roadkill species and those that inhabit the adjacent vegetation) were calculated using 150 bootstraps per species with m = five‐dimensional mMDS space, which was used to estimate the variability of the ordination results.

Then we calculated the TPD at the assemblage level (TPDc), considering their species' relative abundances on each sampled site. Following, we calculated the indices of FRic, functional evenness (FEve), functional divergence (FDiv), and functional redundancy (FR) for each of the 13 landscapes on the road and the adjacent vegetation. These indices were calculated using the TPDs Mean function (Carmona et al., 2019) of the TPD package in the RStudio program version 1.2.5033 (RStudio Team, 2020).

Differences in functional diversity indices of snake assemblages between the road and the vegetation adjacent to the road were analyzed using a permutational multivariate analysis of variance (PERMANOVA), based on Euclidean distances matrix with the sequential sum of squares (Type I) and 9999 permutations of residuals under a reduced model (Anderson et al., 2008) in the software PRIMER 7.0.13 PERMANOVA add‐on (Clarke & Gorley, 2015). The experimental design is a model with one fixed factor (location) and two levels, road, and adjacent vegetation. We included the Longitude and Latitude of each transect as a covariate without interactions with the fixed factor. We calculated the effect size from the estimated variation of the location factor and the two covariables.

To spatially analyze changes in the functional diversity of roadkill snakes at the assemblage level, we performed an inverse distance weighted (IDW) interpolation of each of the functional diversity indices (search radius = 4 and power = 1.5) using ArcGis Pro (v 2.7). We used this method to check if the road, as an environmental filter, affects the values of functional diversity indices, being low on the road and higher further into the adjacent vegetation cover. IDW assumes that each measured point has a local influence that diminishes with distance (Shepard, 1968) and is one of the most used interpolation methods in environmental science (Li & Heap, 2011). We performed a deterministic method instead of a geostatistical method because the number of points we had was insufficient for the second one (Hengl, 2007). For this purpose, the value of each index of functional diversity of roadkill snakes was assigned to the centroid of each landscape on the road. In the case of the functional diversity index values of snakes inhabiting the adjacent vegetation, the value of each index was assigned to the 1000 m on each side of the road.

We evaluated the effect of the scale on functional diversity metrics by using a linear regression between the functional diversity index and the forest NP as the amount of habitat in each area of influence (Jackson & Fahrig, 2015; Miguet et al., 2016; Moraga et al., 2019). We calculated a Spearman correlation test for each land class to identify collinearity (*R* > .75) among the 16 landscape metrics. We carried out a principal coordinate analysis to visualize the variation in landscape metrics for each buffer (250, 500, 1000, and 2000 m). We evaluated the relationship between landscape metrics, considering 7 to 20 noncollinear metrics as predictor variables (Appendices S2–S5), with each one of the four snake functional diversity metrics (i.e., Functional richness, evenness, divergence, and redundancy) from the road and the adjacent vegetation (*n* = 26 for each buffer). We carried out a distance‐based linear model (DistLM; Legendre & Anderson, 1999) and identified the best candidate models that relate snake functional diversity to landscape metrics at the class level, exploring the lowest value of the corrected Akaike's Information Criterion for a small sample size (AICc; McArdle & Anderson, 2001). Using the BEST selection procedure, we identified the more adjusted models per response variable (Functional richness, evenness, divergence, and redundancy) of roadkill snakes assemblages and those found alive inhabiting adjacent vegetation at each measured buffer (250, 500, 1000, and 2000 m) for all possible combinations of predictor variables. These analyses were conducted with the PRIMER 7.0.13 PERMANOVA add‐on (Clarke & Gorley, 2015).

## 3 RESULTS

### 3.1 Variation in the functional traits of roadkill snakes and those found alive in the adjacent vegetation

**FIGURE 1:**
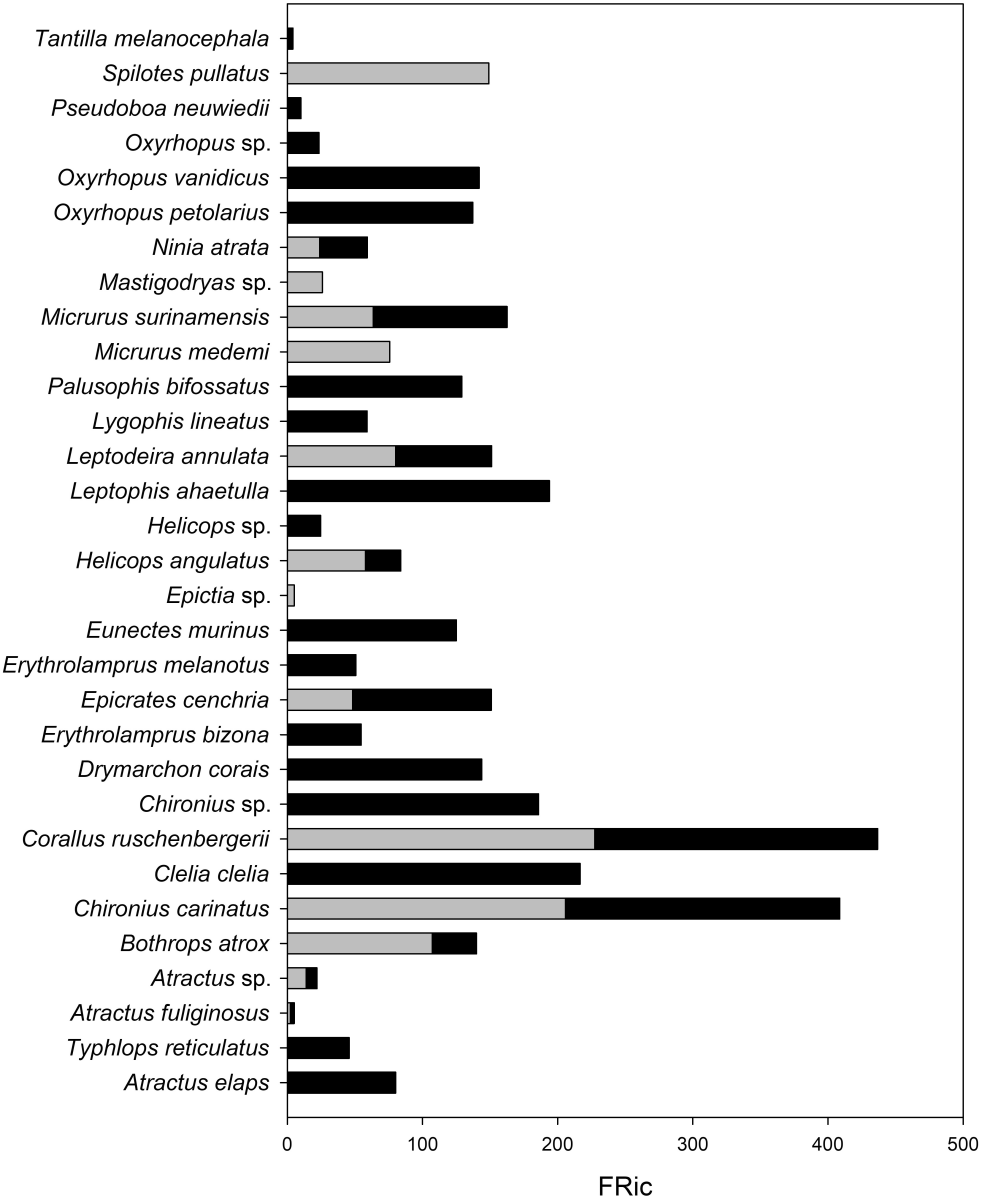
Functional richness values of roadkill snake species (black bar) and those found alive in the adjacent vegetation (gray bar) in the Colombian Orinoco Region. *Corallus ruschenbergerii* showed the highest values of functional richness followed by *Chironius carinatus*, while *Atractus fuliginosus* showed the lowest values.

**FIGURE 2:**
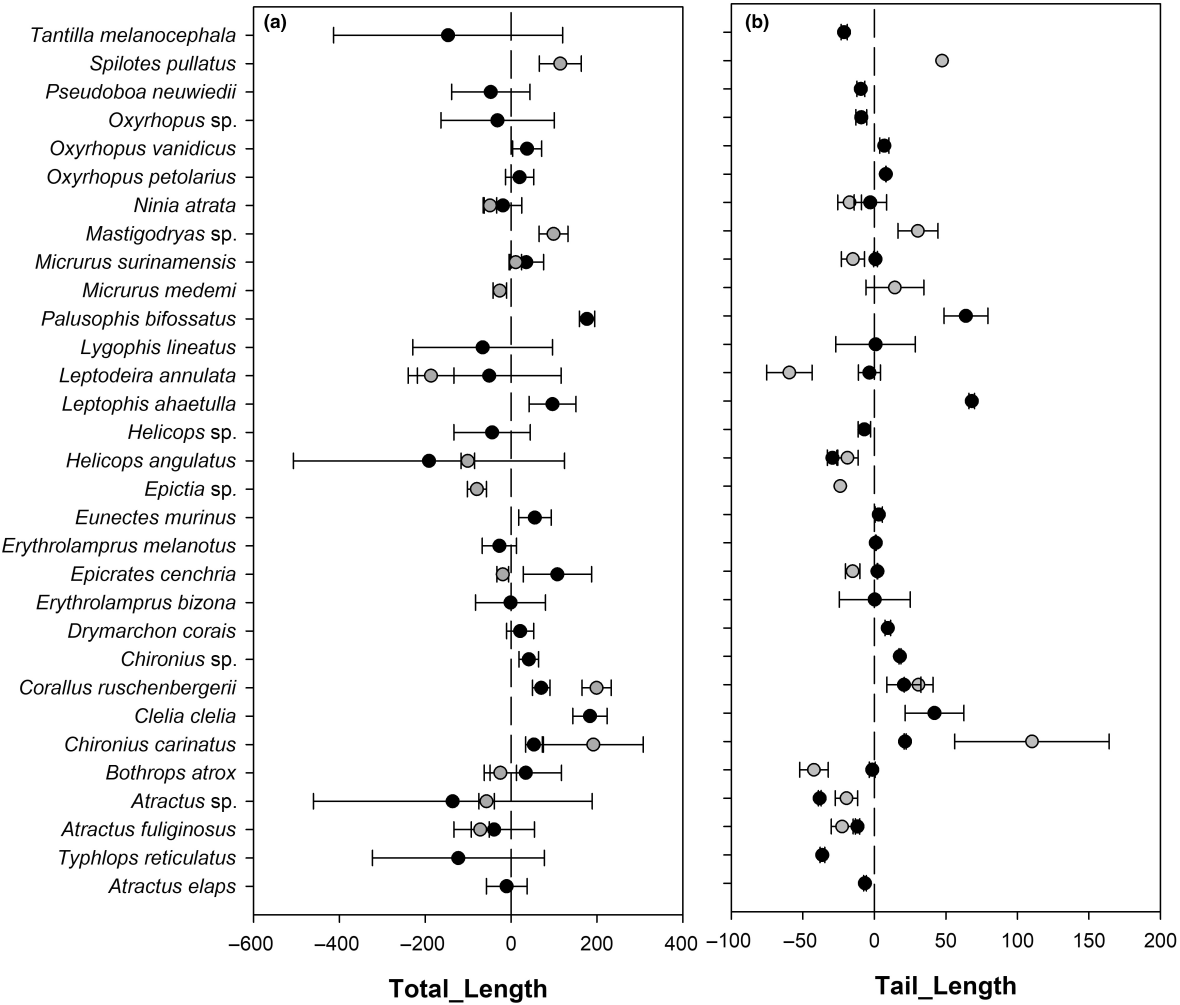
The relative position of species in the Total Length (left) and Tail Length (right) ratio of roadkill snake species (Black circle) and those from the adjacent vegetation (Gray circles) in the Colombian Orinoco Region. The *x*‐axis represents the difference between the species median trait value (Total Length and Tail Length) and the assemblage trait value.

We found 27 species on the road and 14 species on the adjacent vegetation. Ten (10) species were found in both sites (i.e., *Atractus fuliginosus*, *Atractus* sp., *Bothrops atrox*, *Chironius carinatus*, *Corallus ruschenbergerii*, *Epicrates cenchria*, *Helicops angulatus*, *Leptodeira annulata*, *Myrmotherula surinamensis*, and *Ninia atrata*). Two of these, *C. carinatus* and *C. ruschenbergerii*, showed the highest functional richness (FRic_sp) values (Figure 1). Shared species showed higher significant variation in Total Lenght and Tail Lenght in individuals at the adjacent vegetation than that roadkill (Figure 2). Six species (*Atractus* sp., *B. atrox*, *C. ruschenbergerii*, *H. angulatus*, *L. annulata*, and *N. atrata*) showed higher values of FRic_sp on the road compared to the adjacent vegetation, while *E. cenchria* and *M. surinamensis* showed higher values in the adjacent vegetation (Figure 1). At the assemblage level, the Tail Length showed the highest superposition between snake species compared with the Total Length (see Appendix S6).

**FIGURE 3:**
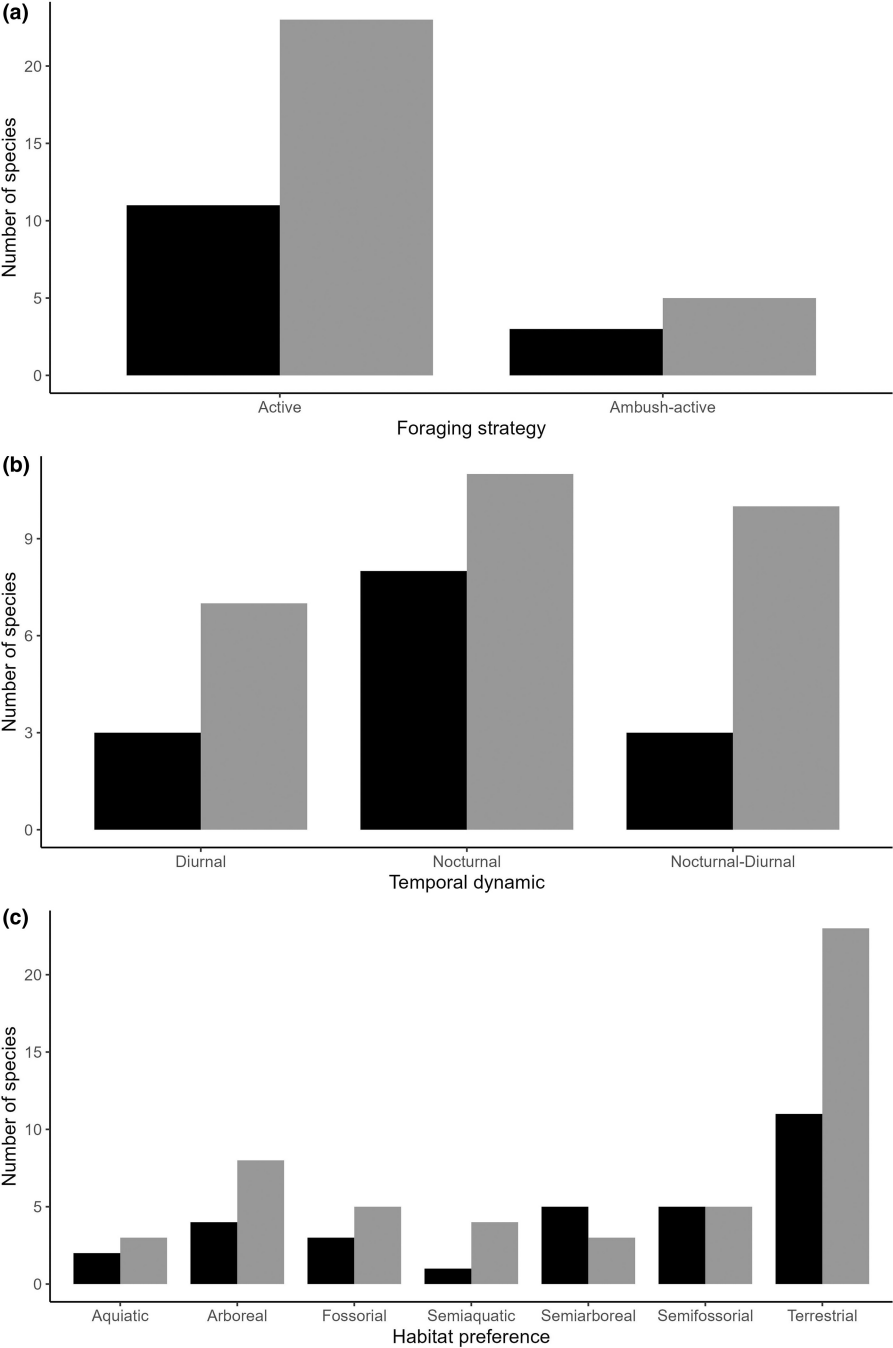
Number of snake species that inhabited the adjacent vegetation (gray bar) and roadkill (black bar) by type of functional life‐history trait: (a) foraging strategy, (b) temporal dynamics of foraging, and (c) habitat preferences.

All species that inhabited the adjacent vegetation and 82% of the roadkill species presented an active foraging strategy (Figure 3a). Regarding the temporal dynamics of foraging, 72% of the species that inhabited the adjacent vegetation were nocturnal, but only 39% of the nocturnal species were road‐killed (Figure 3b). Finally, regarding habitat preferences, all species that inhabited the adjacent vegetation and 82% of the roadkill species were terrestrial (Figure 3c).

**FIGURE 4:**
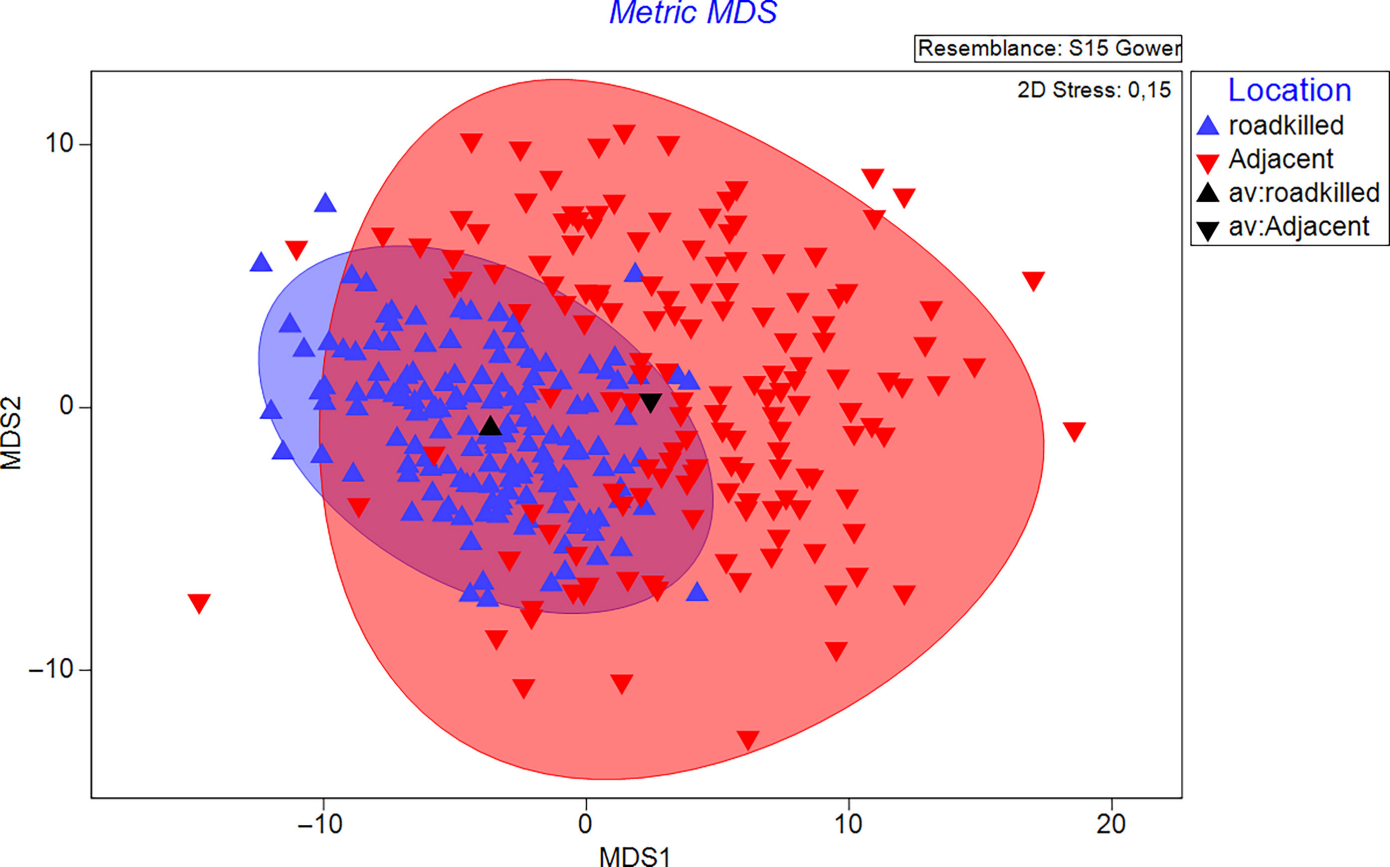
Bootstrap regions for the assemblages of roadkill snakes (blue triangles) and snakes living in the adjacent vegetation (red inverse triangles).

The bootstrap region for the roadkill snake assemblage displays a functional space that is nested within the snake assemblage living in the adjacent vegetation (Figure 4).

### 3.2 Changes in the functional diversity indexes measured at the assemblage level between roadkill snakes and those inhabiting adjacent vegetation

**FIGURE 5:**
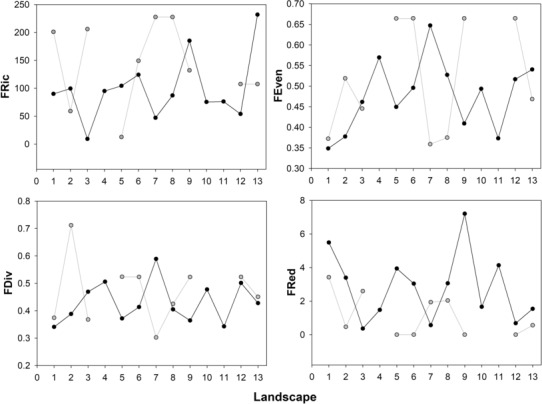
Functional diversity Index values (TPD) for each of the 13 landscapes on the roadkill snake assemblage (grey line) and the snakes from the adjacent vegetation (black line). Functional richness (FRic), Functional evenness (FEve), Functional divergence (FDiv), Functional redundancy (FRed). The 13 values on the *x*‐axis correspond to the 13 sampled sites, and the absence of data in specific sites indicates that the low number of species prevented the calculation of the functional diversity index for those sites.

We did not find statistical differences in functional richness (FRic) (Pseudo‐*F* = 2.67; *p*(perm) = .11), functional evenness (FEve) (Pseudo‐*F* = 0.83; *p*(perm) = .36), and functional divergence (FDiv) (Pseudo‐*F* = 1.12; *p*(perm) = .3) values between roadkill snakes and those found alive in the adjacent vegetation (Table 2). FRic had a slightly greater mean value at the adjacent vegetation (Mean = 142.9; Min = 12.57; Max = 227.5) than at the road (Mean = 98.3; Min = 9.2; Max = 231.76). FEve (Mean = 0.48–0.52; Min = 0.36–0.35; Max = 0.65–0.66) FDiv (Mean = 0.43–0.47; Min = 0.3–0.34; Max = 0.41–0.59) had similar mean and extreme values in both sites. Functional redundancy (FRed) showed statistical differences between the road and the adjacent vegetation (Pseudo‐*F* = 5.33; *p*(perm) = .023) with an effect size of 29.2% for the location factor (Table 2). Also, FRed showed a greater range of variation at the road (Mean = 2.81; Min = 0.36; Max = 7.21) compared with the adjacent vegetation (Mean = 1.1; Min = 0.01; Max = 3.42). Most species found both on the road and the adjacent vegetation tended to occupy periphery positions within the functional trait space of the complete assemblage, based on both measured traits (Total Length and Tail Length) (Figure 5).

**TABLE 2:** Results from the one‐way PERMANOVA analysis of functional diversity indexes between roadkill snakes and snakes inhabiting the adjacent vegetation. The *p*(perm)‐values in bold indicate significant differences in the Pseudo‐*F* test for a specific factor.

Functional diversity index	Source	DF	SS	MS	Pseudo‐*F*	*p*(perm)	Unique perms	Estimates of components of variation		Size effect (%)
								Estimate	Sq. root	
Functional richness (FRic)	Longitude	1	764.96	764.96	0.193	.662	9823	139.31	11.8	2.72
	Latitude	1	13,058	13,058	3.29	.082	9818	468.76	21.65	9.14
	Location	1	10,125	10,125	2.551	.131	9851	549.21	23.44	10.71
	Residuals	19	75,412	3969.1				3969.1	63	77.43
	Total	22	99,360							
Functional evenness (FEve)	Longitude	1	0.02	0.02	1.705	.204	9842	0	0.02	2.85
	Latitude	1	0.003	0.003	0.288	.596	9819	0	0.02	3.41
	Location	1	0.013	0.013	1.091	.313	9831	0	0.01	0.75
	Residuals	19	0.223	0.012				0.01	0.11	92.98
	Total	22	0.26							
Functional divergence (FDiv)	Longitude	1	0	0	0.015	.897	9825	0	0.02	3.99
	Latitude	1	0.007	0.007	0.761	.393	9847	0	0.01	1.15
	Location	1	0.011	0.011	1.217	.28	9845	0	0.01	1.8
	Residuals	19	0.177	0.009				0.01	0.1	93.07
	Total	22	0.196							
Functional redundancy (FRed)	Longitude	1	3.253	3.253	1.042	.322	9815	0.01	0.08	0.12
	Latitude	1	0.836	0.836	0.268	.609	9830	0.12	0.34	2.57
	Location	1	18.125	18.125	5.806	**.023**	9863	1.34	1.16	29.2
	Residuals	19	59.309	3.122				3.12	1.77	68.1
	Total	22	81.523							

### 3.3 Spatial representation of changes in the functional diversity indexes of snakes between road and adjacent vegetation cover

**FIGURE 6:**
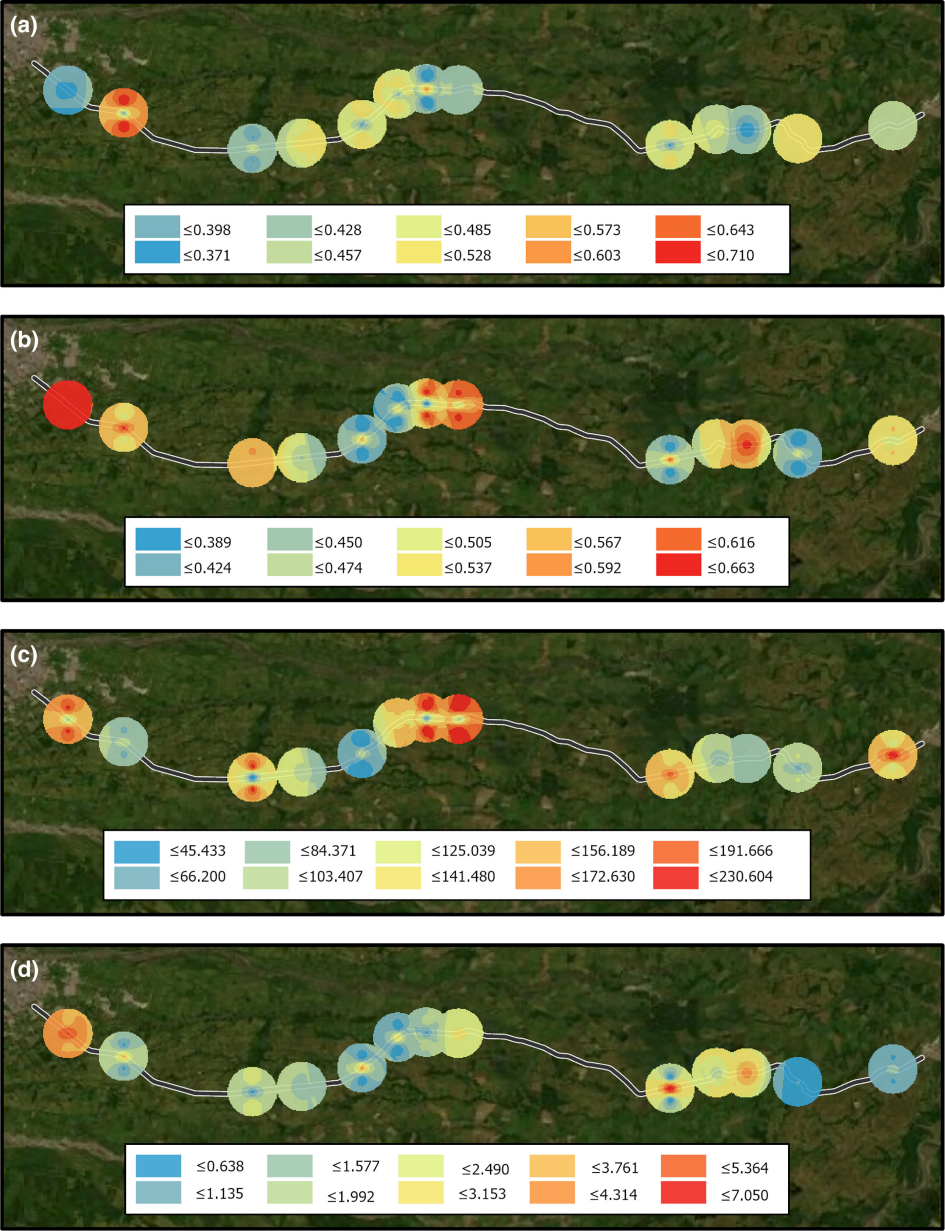
Spatial representation of the functional index through an inverse distance weighted (IDW) interpolation method for the 13 landscapes surveyed at the Colombian Orinoco region. (a) FDiv, (b) FEve, (c) FRic, (d) FRed.

We found variability in the index values across the studied landscapes (Figure 5). The spatial representation of these values for each functional diversity index corroborates this spatial heterogeneity from the road to the adjacent vegetation (Figure 6).

### 3.4 Effect of landscape composition on changes in the functional diversity of roadkill snake assemblages and those found alive in adjacent vegetation across four buffer areas of influence

The effect of the scale of the total amount of native forest (NP forest) on the functional diversity metrics, showed low explanatory power (*R* ^2^ values between <.0001 and .43) across all areas of influence (Appendix S7). However, functional richness (FRic) showed a consistent scale of effect at the 250 m area of influence for roadkill snakes (43%) and those inhabiting the adjacent vegetation (31.2%) (Appendix S7). However, additional vegetation cover classes helped to better predict changes in functional diversity indices at different spatial scales (Table 3).

**TABLE 3:** Summary of significant predictor variables for each response variable at four areas of influence (from 250 to 3000 m of measured spatial distances from the sampled site) in the best‐fit models (See Appendix S8).

Functional diversity index	Area of influence	Roadkill (% explained)	Living in adjacent vegetation (% explained)
Functional richness (FRic)	250	CA forest (43.3%)	COHESION pasture (36.9%)
Functional evenness (FEve)	500	NP forest (27.8%)	
	1000		AREA_SD pastures (49.7%)
	2000	PD_pasture (48.4%); PD_Transitory_crop (41.5%)	AREA_MN forest (62.4%); ENN_SD forest (56.6%)
Functional divergence (FDiv)	1000	PARA_MN bodies of water (42.7%)	AREA_SD pastures (41%)
	2000	PD_Transitory_crop (37.1%)	
Functional redundancy (FRed)	2000	ENN_SD pastures (38.7%)	AREA_MN forest (61.7%)

Functional diversity was explained from a 250 m area of influence (in the case of functional richness or FRic) to 2000 m in the case of functional redundancy (FRed), functional evenness (FEve), and functional divergence (FDiv). For roadkill snakes, at local scales (between 250 and 500 m area of influence), the total forest area and the number of native forest patches explained changes in FRic and FEve, respectively. While at 2000 m area of influence, the density of transient crop patches explained changes in FEve and FDiv, and the standard deviation of pastures (Euclidean nearest neighbor distance) explained changes in FRed (Table 3). For snakes inhabiting the adjacent vegetation, the cohesion of pasture explained changes in FRic at 250 m of the area of influence. In comparison, at 2000 m the mean patch area of the native forest explained changes in FRed and its interaction with mean Euclidean nearest neighbor distance between native forest explained FEve (Table 3).

## 4 DISCUSSION

Anthropogenic changes in land use impose environmental filters on reptile assemblages (Todd et al., 2017), and evidence suggests an increase in functional redundancy and functionally homogenizing (Adams et al., 2022). In this study, we found that the effect of landscape composition on functional diversity was higher than roadkill effects for snake assemblages along 13 landscapes on the road located in the Orinoco region of Colombia.

### 4.1 Roadkill snakes: From functional traits to functional diversity indices

In contrast with previous reports of lower functional richness and divergence (FRic and FDiv, respectively) in disturbed sites (Adams et al., 2022; Berriozabal‐Islas et al., 2017), we did not find statistical differences between roadkill and adjacent vegetation snake assemblages. However, the amount of functional space occupied by roadkill snakes was slightly small compared with the snake assemblages inhabiting adjacent vegetation, even though the roadkill species number was the highest. In addition, we found a pattern of higher functional richness values on six roadkill species (i.e., *Atractus* sp., *B. atrox*, *C. ruschenbergerii*, *H. angulatus*, *L. annulata*, and *N. atrata*), indicating higher intraspecific variation in body and tail length than those found in the adjacent vegetation. Our results point to excluding high heterogeneity of individuals from the snake assemblage. Snake body and tail length variation can occur at the intraspecific level because of the ontogenetic variation. Thus, ontogenetic niche changes refer to habitat use changes expressed in predation risk, motor skills, and thermal response norms (Blouin‐Demers et al., 2007). For example, *B. atrox* changes its dietary preferences between juvenile and adult stages and its microhabitat use (Silva et al., 2019). Also, in some snake species, adults tend to be more selective in their habitat use than juveniles. Individuals at early ages of development show less fidelity to a certain microhabitat (Blouin‐Demers et al., 2007). Also, prominent individuals select forest edges because these habitats facilitate thermoregulation (Blouin‐Demers & Weatherhead, 2001; Wagner et al., 2021).

Our results support the hypothesis that terrestrial species are more likely to be roadkill. However, it should be noted that some snakes that are run over may also be arboreal or fossorial. This pattern of snake roadkill, related to species habitat preference, had been previously reported by Sosa and Schalk (2016) across diverse ecosystems in central Bolivia, who draw attention to the importance of seasonality in rainfall for a better understanding of road ecology. We suggest that the overrun of aquatic and semi‐aquatic species (22% of the roadkill species) could be explained because, during the rainy season, the water channels designed to ensure the drainage of roads in Colombia (INVIAS, 2009) overflow and flood the road, allowing the temporary dispersion of these species on the road. Likewise, we believe that, during the rainy season, when the subterranean habitats of fossorial and semi‐fossorial snakes (35% of the roadkill species) are flooded, these species are forced to seek higher ground, reaching the road. In both cases, these (semi‐)aquatic and (semi‐)fossorial snake species are trapped on the road when the water level drops, leaving them at a high risk of being run over. In this sense, it is important that future studies evaluate changes in the value of functional traits and functional diversity indices considering variation in seasonality and even in the type of road construction (Machado et al., 2015).

However, the relationship between daily temporal dynamics in foraging and snake strike is still unclear. The present study's results do not support the hypothesis that nocturnal snakes would be more likely to be run over. We expected higher mortality in nocturnal snakes because, at the beginning of the night, individuals tend to seek the heat accumulated on the asphalt (Das et al., 2007; Hallisey et al., 2022), increasing the probability of being run over by drivers who also only manage to detect the snake on the road when they are too close to avoid it. However, 25% of the roadkill species show a temporal dynamic of diurnal foraging and 36% present a diurnal and nocturnal dynamic. The lack of consistent patterns in snake strike‐downs based on their temporal foraging dynamics may be due to behavioral and ecophysiological traits not explored in the present study. Still, they also call for the need for robust databases of species' life‐history traits, as they have been consolidated for other groups such as plants (Kattge et al., 2011), fish (Brosse et al., 2021; Mull et al., 2022), birds (Tobias et al., 2022) and amphibians (Oliveira et al., 2017).

The trend to lower value of FEve on roadkill snakes shows that the probabilities associated with tail and total length values were less even (i.e., uneven relative abundances) on the road than for the adjacent vegetation. This result contrasts with the one reported by Adams et al. (2022), who found higher FEve values for snake assemblages at sites with heavy anthropogenic management (induced fires and thinning operations). However, results seem to differ among study groups. For instance, Berriozabal‐Islas et al. (2017) found higher FEve values for lizards in mature natural environments without human disturbance. Snake species tend to occupy peripheral positions within the entire assemblage trait distribution. Therefore, we can suggest that most species contribute to community and ecosystem function.

The highest redundancy (FRed) on roadkill snakes suggests that they occupy the same functional space in the assemblage despite the greatest number of snake species registered. This is a functional space that increases vulnerability to being run over. Roadkill species showed a narrow range (or an aggregated pattern) of size values in the functional space, a pattern like that observed with snake assemblages on burned sites and with thinning operations (Adams et al., 2022).

### 4.2 Effect of landscape composition on functional diversity at multiple scales for roadkill species and adjacent vegetation assemblages

Recent reviews found that snake species with small size ranges tend to respond negatively to landscape transformation, even though no direct effect from body size was found for some species (Doherty et al., 2019; Todd et al., 2017). Some studies suggest that small individuals move more frequently but travel shorter distances, narrowing their range (Blouin‐Demers et al., 2007). Other studies propose a relationship between body size, the degree of habitat specialization, and the risk of extinction of Squamates but with no emphasis on snakes (Böhm et al., 2016; Tingley et al., 2013). In this sense, we believe it is essential to evaluate the effect of landscape composition on the functional diversity of snakes in different areas of influence, representing variation in the dispersal capacities of the species within the assemblage (following Driscoll et al., 2013).

We found that the most significant estimated component of variation for all functional diversity indices resides in the residuals (Table 2), indicating that the variance of these indices within each local landscape is greater than the variance between landscapes. This result suggests that the high heterogeneity in the composition of each landscape shapes the snake assemblages but with differential effects of landscape metrics between roadkill species and assemblages inhabiting adjacent vegetation. The habitat heterogeneity hypothesis, one of the foundational concepts in community ecology, suggests that structurally complex habitats provide more ecological niches and resources for a wide variety of species to exploit, thus supporting more diverse species assemblages (Bazzaz, 1975). Many examples of heterogeneous habitats provide varied resources (structural and provisional) for a larger number of species than more homogeneous habitats (Kissling et al., 2008; McElhinny et al., 2005; Tews et al., 2004). For example, functional richness (FRic) values generally are explained by landscape metrics related to forest and pasture classes. However, because these metrics are associated with the extension of the landscape, the best explanatory class metrics changed according to the area of influence.

The scale of the effect evaluated exclusively from the amount of native forest (as the only available habitat for snakes) underestimated the effect of landscape composition on functional diversity (see Appendix S7). While landscape metrics for the native forest class explained changes in functional richness and evenness (FRic and FEve, respectively) of road‐killed snakes and for functional redundancy and evenness (FRed and FEve, respectively) of snakes inhabiting adjacent vegetation. Some anthropogenic cover types such as pastures and transitory crops, as well as water bodies, help to complement our understanding of the scale of the effect on snake functional diversity, including the running over of (semi‐)aquatic and fossorial species during the rainy season; even functional divergence (FDiv) was explained exclusively by other covers different to native forest.

The functional diversity of snakes from adjacent vegetation was expected to be explained by different natural and anthropogenic classes at 1000 and 2000 m. The last was fulfilled since this assemblage was explained from the 1000 m area of influence for functional evenness and divergence (from changes in metrics of pastures) and from 2000 m for functional redundancy and evenness (from changes in metrics of the forest). While functional richness was explained at an influence area minor than 1000 m. Many snake species quickly adapt to anthropogenically influenced landscapes if food is available, as individuals may move over short distances and less frequently (Parent & Weatherhead, 2000). In contrast, other species prefer natural open environments (such as savannahs) and even anthropogenic environments (such as cattle pastures), suggesting that habitat quality at local scales, in terms of resource availability, may be more critical for reptiles than the configuration of the whole landscape (Cunningham et al., 2007; Fischer et al., 2004).

We hypothesized that the amount of forest would explain the functional diversity of roadkill snakes at smaller spatial scales (250 and 500 m of the sampling site). The interpolation analysis confirmed this hypothesis, showing that the functional richness index (FRic) trend to increase in sites with higher forest coverage. A general pattern found is that the FRic was explained by forest cover (for roadkill snakes) and pasture (for snakes inhabiting adjacent vegetation) at an area of influence of 250 m. The importance of small landscape elements for reptile dispersal has been previously pointed out in neotropical forests in Costa Rica (Mendenhall et al., 2014), so future studies of the scale of the effect should include areas of influence at more detailed resolutions (e.g., those obtained by remote sensing with drone overflights to show coverage heterogeneity; Duarte‐Ballesteros et al., 2021). Our results confirm the hypothesis that species with active foraging strategies tend to be more roadkill likely due to their greater ability to scan the environment and disperse across the landscape (Yue et al., 2019). In this sense, dispersal ability varies according to landscape composition and its effect at different scales at the assemblage level (Ramírez‐Mejía et al., 2020) or the species level, depending on sex, scale, and discrete categories of land use (Ramírez‐Mejía et al., 2020). Therefore, we suggest that road ecology should be strengthened by integrating landscape ecology at different spatial scales and involving functional trait ecology to better understand the causes and consequences of wildlife roadkill.

## 5 CONCLUSIONS

In contrast with our expectation, results did not show higher values for functional diversity indices of snake assemblages inhabiting the adjacent vegetation than roadkill snakes' diversity. Regarding the ecology of the snakes, roadkill is reducing the population sizes of some species even though they have high functional redundancy. The effect of roadkill is eliminating species that play similar functional roles in the assemblage and the ecosystem processes and services (i.e., from nutrient cycling and energy flow through food chains in their role as prey to pest control, secondary seed dispersal, and pharmacological properties of venoms, among others; Cortés‐Gomez et al., 2015; Reiserer et al., 2018). We consider it essential to emphasize that the functional redundancy of snakes in the adjacent vegetation was significantly lower than that on the roads, highlighting these assemblages' high vulnerability to landscape transformation. Despite not finding statistically significant differences in other indices of functional diversity among landscapes, the absolute patterns of the functional diversity index between both snake assemblages varied across the landscape (Figure 5). Although the absence of differences in the values of functional diversity indices between roadkill snakes and those inhabiting the adjacent vegetation may indicate functional homogenization of snake assemblages in the study landscapes, future research should investigate changes in functional diversity by differentiating between landscape elements that constitute snake habitat (e.g., Mendenhall et al., 2014) at a finer sampling grain. This will help to identify the adequate spatial scale to understand the study phenomenon, conduct the sampling, and run the statistical analyses (as recently proposed by Fletcher et al., 2023).

All functional diversity indexes of roadkill snakes and those from adjacent vegetation were predicted by forest metrics, pastures, bodies of water, and transitory crops, suggesting the need to conduct future studies at the interpopulation level for different assemblage species. Our results reinforce the need to continue exploring the scale of the effect at which habitat quantity is measured (Watling et al., 2020) and to include species response to edge effect, as species in an assemblage may extensively use the anthropogenic matrix, be habitat specialists inside the native forest or be generalists to vegetation cover gradients (Schneider‐Maunoury et al., 2016). In this sense, studies of edge effects should be considered in future studies on landscape composition and configuration in biotic communities (Pfeifer et al., 2017).

## AUTHOR CONTRIBUTIONS

**Mónica Rincón‐Aranguri:** Conceptualization (equal); data curation (lead); funding acquisition (lead); investigation (lead); methodology (equal); writing – original draft (equal). **Felipe A. Toro:** Conceptualization (equal); data curation (supporting); formal analysis (equal); methodology (equal); visualization (equal); writing – review and editing (equal). **Sandra P. Galeano:** Conceptualization (equal); investigation (supporting); methodology (equal); supervision (equal); writing – review and editing (equal). **Lilia Roa‐Fuentes:** Formal analysis (equal); methodology (supporting); validation (equal); visualization (equal); writing – review and editing (equal). **Nicolás Urbina‐Cardona:** Conceptualization (equal); data curation (supporting); formal analysis (equal); investigation (supporting); methodology (equal); supervision (equal); visualization (equal); writing – original draft (equal).

## FUNDING INFORMATION

This research was funded by the Fondo para la Educación Superior, Gobernación del Meta (Beca de Maestría Resolución 333–2015), Fundación Alejandro Ángel Escobar (Beca Colombia Biodiversa II‐2016). Crowdfunding The Highway of death: Snakes vs. Cars (Experiment Crowdfunding 7‐05‐2017).

## CONFLICT OF INTEREST STATEMENT

The authors declare that there are no potential conflicts of interest concerning this article's research, authorship, and publication.

## Supporting information


Appendix S1–S8

## Data Availability

Upon publication, the raw data will be available at “Functional diversity of snakes is explained by the landscape composition at multiple areas of influence”, Mendeley Data, DOI: 10.17632/wp8gw23kd2.1.
