# Supplementary material for: Functional diversity of snakes is explained by the landscape composition at multiple areas of influence
Source: Ecol Evol. 2023 Jul 26;13(7):e10352. doi: 10.1002/ece3.10352 (PMC10369374; doi:10.1002/ece3.10352)
Supplement: Supplementary file 1 — Appendix S1–S8 [file ECE3-13-e10352-s001.docx]

Supporting information:

*Ecology and Evolution*

**Functional diversity of snakes is explained by the landscape composition at multiple areas of influence**

Mónica Rincón-Aranguri1,2, Felipe A. Toro-Cardona3, Sandra P. Galeano4, Lilia Roa-Fuentes1, Nicolás Urbina-Cardona1*

1 Pontificia Universidad Javeriana, Facultad de Estudios Ambientales y Rurales, Departamento de Ecología y Territorio, Bogotá, Colombia.

2 Grupo Herpetológico de Antioquia - Instituto de Biología - Universidad de Antioquia, Medellín, Colombia.

3 Grupo de Ecología y Evolución de Vertebrados, Instituto de Biología, Universidad de Antioquia, Medellín, Colombia.

4 Instituto de Investigación de Recursos Biológicos Alexander von Humboldt, Villa de Leyva, Colombia.

*Corresponding author: Nicolás Urbina-Cardona

E-mail: [urbina-j@javeriana.edu.co](mailto:urbina-j@javeriana.edu.co)

Supporting information for Rincón-Aranguri et al., “Landscape composition and functional diversity of snakes,” E&E

**Functional diversity of snakes is explained by the landscape composition at multiple areas of influence**

*Mónica Rincón-Aranguri, Felipe A. Toro-Cardona, Sandra P. Galeano, Lilia Roa-Fuentes, and Nicolás Urbina-Cardona*

This document contains all appendices:

**Appendix S1:** Location of the study area in the Colombian Orinoco Region. White circles represent the thirteen sampled landscapes along the paved highway (red line). The dark green polygon represents the eastern cordillera of Colombia, and the blue lines represent the two main rivers found in the region.

**Appendix S2:** Spearman correlation between 16 landscape metrics for each land class at 250 m area of influence. We found 11 independent (non-collinear) metrics at six classes: urban infrastructure (CA and PD), pastures (CA, PD and COHESION), forest (CA, PARA_MN, and COHESION), CA of transitory crops, CA of bodies of water and CA of permanent crops. The link for downloading the spreadsheet online is: <https://docs.google.com/spreadsheets/d/1N3pfuOvuHY8I6MCeoah7X7CsHO6J4bsD6dAkwXfmwFI/edit?usp=sharing>

**Appendix S3:** Spearman correlation between 16 landscape metrics for each land class at 500 m area of influence. We found 10 independent metrics at four classes: Pastures (CA, PD, AREA_MN and PARA_SD), Forest (PLAND, NP and PD), Bodies of water (CA and PLAND) and AREA_MN of transitory crops. The link for downloading the spreadsheet online is: <https://docs.google.com/spreadsheets/d/1N3pfuOvuHY8I6MCeoah7X7CsHO6J4bsD6dAkwXfmwFI/edit?usp=sharing>

**Appendix S4:** Spearman correlation between 16 landscape metrics for each land class at 1000 m area of influence. We found 7 independent metrics at three classes: Pastures (CA, AREA_SD and CONTIG_MN) Bodies of water (AREA_MN and PARA_MN) and Urban buildings (CONTIG_SD and COHESION). The link for downloading the spreadsheet online is: <https://docs.google.com/spreadsheets/d/1N3pfuOvuHY8I6MCeoah7X7CsHO6J4bsD6dAkwXfmwFI/edit?usp=sharing>

**Appendix S5:** Spearman correlation between 16 landscape metrics for each land class at 2000 m area of influence. We found 20 independent metrics at five classes: Pastures (PD, AREA_MN, PARA_MN, ENN_SD, and COHESION), Urban buildings (PD and AREA_SD), Water of bodies (CA and GYRATE_SD), Forests (CA, PD, AREA_MN, AREA_SD, ENN_MN, and ENN_SD), and Transitory crops (CA, PD, AREA_MN, PARA_MN and COHESION). The link for downloading the spreadsheet online is: <https://docs.google.com/spreadsheets/d/1N3pfuOvuHY8I6MCeoah7X7CsHO6J4bsD6dAkwXfmwFI/edit?usp=sharing>

**Appendix S6:** Inter-species variability of snakes based on two functional traits: LTotal = Total length; Cola = Tail length. A. killed on the highway; and B. alive on adjacent vegetation in the Colombian Orinoco Region.

**Appendix S7:** Scale of the effect of habitat amount (NP forest landscape metric) on functional diversity indices of road-killed snakes and those inhabiting surrounding vegetation. The scale of the effect is that area of influence at which the R2 value of the linear regression between the amount of native forest and the index of functional diversity evaluated is greater (highlighted in bold).

**Appendix S8:** Best fitted models explaining changes in functional diversity indices on the road and adjacent coverages at four areas of influence (from 250 to 3000 m of measured spatial distances from the sampled site). For the best fitted models, we report for each variable the pseudo F test statistic, its p-value and its percentage of explanation. The link for downloading the spreadsheet online is: <https://docs.google.com/spreadsheets/d/1N3pfuOvuHY8I6MCeoah7X7CsHO6J4bsD6dAkwXfmwFI/edit?usp=sharing>

**Appendix S1:**


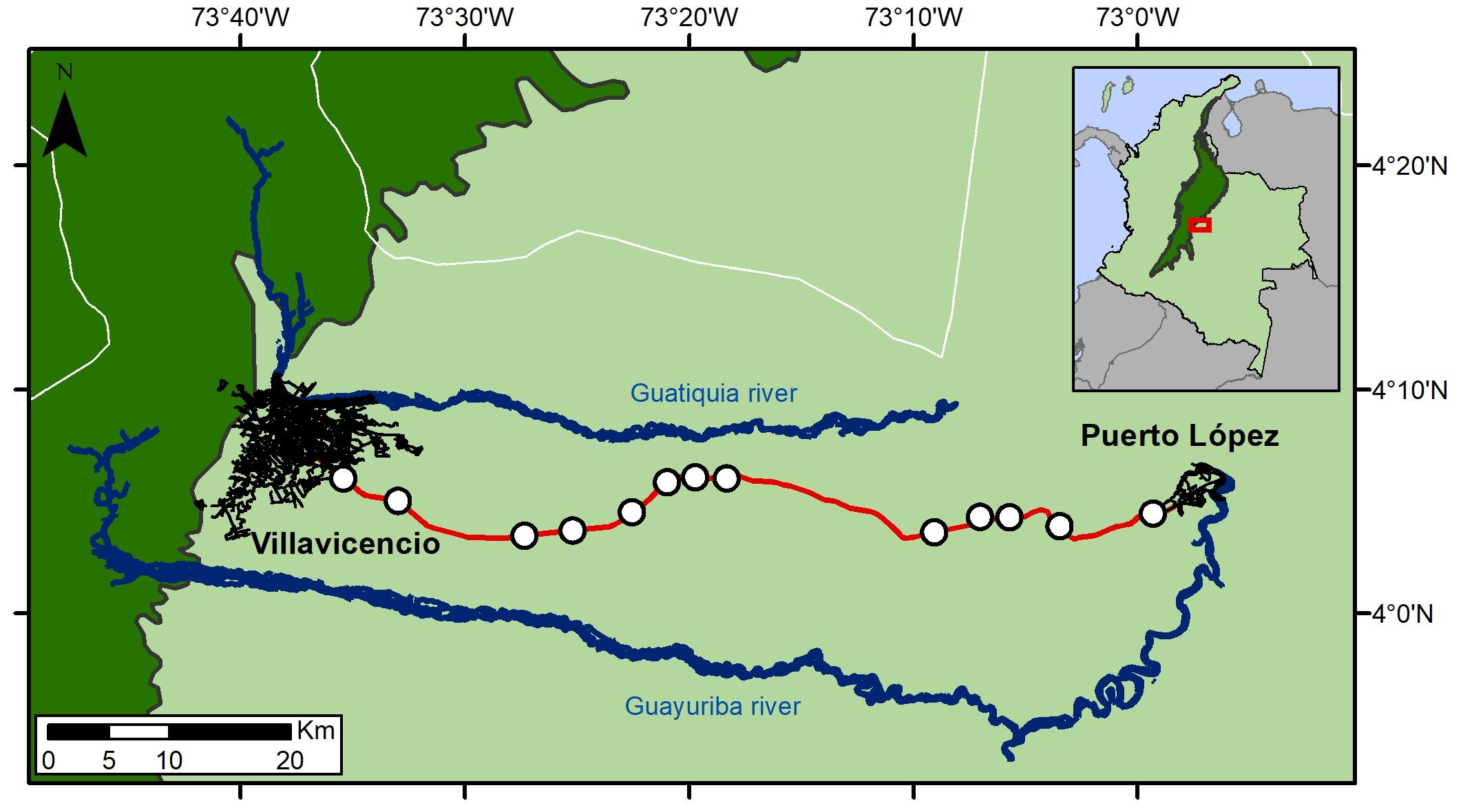


**Figure S1.** Location of the study area in the Colombian Orinoco Region. White circles represent the thirteen sampled landscapes along the paved highway (red line). The dark green polygon represents the eastern cordillera of Colombia, and the blue lines represent the two main rivers found in the region.

**Appendix S2:**

**Table S1**  Spearman correlation between 16 landscape metrics for each land class at 250 m area of influence. We found 11 independent (non-collinear) metrics at six classes: urban infrastructure (CA and PD), pastures (CA, PD and COHESION), forest (CA, PARA_MN, and COHESION), CA of transitory crops, CA of bodies of water and CA of permanent crops. The link for downloading the spreadsheet online is: https://docs.google.com/spreadsheets/d/1N3pfuOvuHY8I6MCeoah7X7CsHO6J4bsD6dAkwXfmwFI/edit?usp=sharing

| **250 m buffer** | **Metric ID** | 1 | 2 | 3 | 4 | 5 | 6 | 7 | 8 | 9 | 10 | 11 | 12 | 13 | 14 | 15 | 16 | 17 | 18 | 19 | 20 | 21 | 22 | 23 | 24 | 25 | 26 | 27 | 28 | 29 | 30 | 31 | 32 | 33 | 34 | 35 | 36 | 37 | 38 | 39 | 40 | 41 | 42 |
| --- | --- | --- | --- | --- | --- | --- | --- | --- | --- | --- | --- | --- | --- | --- | --- | --- | --- | --- | --- | --- | --- | --- | --- | --- | --- | --- | --- | --- | --- | --- | --- | --- | --- | --- | --- | --- | --- | --- | --- | --- | --- | --- | --- |
| CA Urban infrastructure | 1 |  |  |  |  |  |  |  |  |  |  |  |  |  |  |  |  |  |  |  |  |  |  |  |  |  |  |  |  |  |  |  |  |  |  |  |  |  |  |  |  |  |  |
| PLAND Urban infrastructure | 2 | 1.00 |  |  |  |  |  |  |  |  |  |  |  |  |  |  |  |  |  |  |  |  |  |  |  |  |  |  |  |  |  |  |  |  |  |  |  |  |  |  |  |  |  |
| NP Urban infrastructure | 3 | 0.84 | 0.84 |  |  |  |  |  |  |  |  |  |  |  |  |  |  |  |  |  |  |  |  |  |  |  |  |  |  |  |  |  |  |  |  |  |  |  |  |  |  |  |  |
| PD Urban infrastructure | 4 | 0.74 | 0.84 | 1.00 |  |  |  |  |  |  |  |  |  |  |  |  |  |  |  |  |  |  |  |  |  |  |  |  |  |  |  |  |  |  |  |  |  |  |  |  |  |  |  |
| AREA_MN Urban infrastructure | 5 | 1.00 | 1.00 | 0.84 | 0.84 |  |  |  |  |  |  |  |  |  |  |  |  |  |  |  |  |  |  |  |  |  |  |  |  |  |  |  |  |  |  |  |  |  |  |  |  |  |  |
| PARA_MN Urban infrastructure | 6 | 0.67 | 0.67 | 0.97 | 0.97 | 0.67 |  |  |  |  |  |  |  |  |  |  |  |  |  |  |  |  |  |  |  |  |  |  |  |  |  |  |  |  |  |  |  |  |  |  |  |  |  |
| COHESION Urban infrastructure | 7 | 0.84 | 0.84 | 1.00 | 1.00 | 0.84 | 0.97 |  |  |  |  |  |  |  |  |  |  |  |  |  |  |  |  |  |  |  |  |  |  |  |  |  |  |  |  |  |  |  |  |  |  |  |  |
| MESH Urban infrastructure | 8 | 0.98 | 0.98 | 0.72 | 0.72 | 0.98 | 0.52 | 0.73 |  |  |  |  |  |  |  |  |  |  |  |  |  |  |  |  |  |  |  |  |  |  |  |  |  |  |  |  |  |  |  |  |  |  |  |
| CA Pastures | 9 | -0.34 | -0.34 | -0.05 | -0.05 | -0.34 | 0.08 | -0.06 | -0.41 |  |  |  |  |  |  |  |  |  |  |  |  |  |  |  |  |  |  |  |  |  |  |  |  |  |  |  |  |  |  |  |  |  |  |
| NP Pastures | 10 | 0.63 | 0.63 | 0.39 | 0.39 | 0.63 | 0.24 | 0.40 | 0.67 | -0.26 |  |  |  |  |  |  |  |  |  |  |  |  |  |  |  |  |  |  |  |  |  |  |  |  |  |  |  |  |  |  |  |  |  |
| PD Pastures | 11 | 0.63 | 0.63 | 0.39 | 0.39 | 0.63 | 0.24 | 0.40 | 0.67 | -0.26 | 1.00 |  |  |  |  |  |  |  |  |  |  |  |  |  |  |  |  |  |  |  |  |  |  |  |  |  |  |  |  |  |  |  |  |
| AREA_MN Pastures | 12 | -0.36 | -0.36 | -0.06 | -0.06 | -0.36 | 0.08 | -0.07 | -0.44 | 0.95 | -0.50 | -0.50 |  |  |  |  |  |  |  |  |  |  |  |  |  |  |  |  |  |  |  |  |  |  |  |  |  |  |  |  |  |  |  |
| AREA_SD Pastures | 13 | 0.20 | 0.20 | 0.08 | 0.08 | 0.20 | 0.02 | 0.09 | 0.23 | -0.05 | 0.88 | 0.88 | -0.36 |  |  |  |  |  |  |  |  |  |  |  |  |  |  |  |  |  |  |  |  |  |  |  |  |  |  |  |  |  |  |
| PARA_MN Pastures | 14 | 0.42 | 0.42 | 0.25 | 0.25 | 0.42 | 0.14 | 0.25 | 0.45 | -0.63 | 0.29 | 0.29 | -0.61 | 0.09 |  |  |  |  |  |  |  |  |  |  |  |  |  |  |  |  |  |  |  |  |  |  |  |  |  |  |  |  |  |
| PARA_SD Pastures | 15 | 0.96 | 0.96 | 0.66 | 0.66 | 0.96 | 0.45 | 0.67 | 0.99 | -0.43 | 0.73 | 0.73 | -0.48 | 0.32 | 0.45 |  |  |  |  |  |  |  |  |  |  |  |  |  |  |  |  |  |  |  |  |  |  |  |  |  |  |  |  |
| COHESION Pastures | 16 | 0.03 | 0.03 | 0.07 | 0.07 | 0.03 | 0.08 | 0.07 | 0.02 | 0.47 | 0.06 | 0.06 | 0.43 | 0.06 | -0.88 | 0.02 |  |  |  |  |  |  |  |  |  |  |  |  |  |  |  |  |  |  |  |  |  |  |  |  |  |  |  |
| MESH Pastures | 17 | -0.23 | -0.23 | 0.03 | 0.03 | -0.23 | 0.15 | 0.03 | -0.31 | 0.95 | -0.29 | -0.29 | 0.94 | -0.17 | -0.42 | -0.34 | 0.29 |  |  |  |  |  |  |  |  |  |  |  |  |  |  |  |  |  |  |  |  |  |  |  |  |  |  |
| CA Native forest | 18 | 0.05 | 0.05 | -0.06 | -0.06 | 0.05 | -0.10 | -0.06 | 0.08 | 0.12 | 0.65 | 0.65 | -0.13 | 0.79 | -0.03 | 0.16 | 0.11 | -0.02 |  |  |  |  |  |  |  |  |  |  |  |  |  |  |  |  |  |  |  |  |  |  |  |  |  |
| AREA_MN Native forest | 19 | -0.07 | -0.07 | -0.14 | -0.14 | -0.07 | -0.16 | -0.14 | -0.04 | 0.17 | 0.57 | 0.57 | -0.08 | 0.77 | -0.09 | 0.04 | 0.11 | 0.02 | 0.99 |  |  |  |  |  |  |  |  |  |  |  |  |  |  |  |  |  |  |  |  |  |  |  |  |
| AREA_SD Native forest | 20 | 0.97 | 0.97 | 0.67 | 0.67 | 0.97 | 0.46 | 0.68 | 1.00 | -0.44 | 0.67 | 0.67 | -0.46 | 0.24 | 0.45 | 1.00 | 0.01 | -0.33 | 0.09 | -0.03 |  |  |  |  |  |  |  |  |  |  |  |  |  |  |  |  |  |  |  |  |  |  |  |
| PARA_MN Native forest | 21 | 0.16 | 0.16 | 0.52 | 0.52 | 0.16 | 0.64 | 0.52 | 0.02 | 0.19 | -0.11 | -0.11 | 0.21 | -0.12 | -0.14 | -0.04 | 0.14 | 0.16 | -0.18 | -0.17 | -0.03 |  |  |  |  |  |  |  |  |  |  |  |  |  |  |  |  |  |  |  |  |  |  |
| COHESION Native forest | 22 | 0.37 | 0.37 | 0.38 | 0.38 | 0.37 | 0.34 | 0.38 | 0.34 | 0.03 | 0.51 | 0.51 | -0.10 | 0.45 | -0.02 | 0.36 | 0.21 | -0.07 | 0.64 | 0.60 | 0.33 | 0.44 |  |  |  |  |  |  |  |  |  |  |  |  |  |  |  |  |  |  |  |  |  |
| MESH Native forest | 23 | -0.13 | -0.13 | -0.17 | -0.17 | -0.13 | -0.18 | -0.17 | -0.11 | 0.18 | 0.59 | 0.59 | -0.09 | 0.84 | -0.10 | -0.02 | 0.10 | 0.03 | 0.96 | 0.98 | -0.10 | -0.16 | 0.51 |  |  |  |  |  |  |  |  |  |  |  |  |  |  |  |  |  |  |  |  |
| CA Transitory crops | 24 | -0.34 | -0.34 | -0.41 | -0.41 | -0.34 | -0.39 | -0.41 | -0.29 | -0.57 | -0.41 | -0.41 | -0.44 | -0.36 | 0.29 | -0.30 | -0.48 | -0.52 | -0.46 | -0.42 | -0.27 | -0.47 | -0.64 | -0.39 |  |  |  |  |  |  |  |  |  |  |  |  |  |  |  |  |  |  |  |
| PLAND Transitory crops | 25 | -0.34 | -0.34 | -0.41 | -0.41 | -0.34 | -0.39 | -0.41 | -0.29 | -0.57 | -0.41 | -0.41 | -0.44 | -0.36 | 0.29 | -0.30 | -0.48 | -0.52 | -0.46 | -0.42 | -0.27 | -0.47 | -0.64 | -0.39 | 1.00 |  |  |  |  |  |  |  |  |  |  |  |  |  |  |  |  |  |  |
| AREA_MN Transitory crops | 26 | -0.34 | -0.34 | -0.41 | -0.41 | -0.34 | -0.39 | -0.41 | -0.29 | -0.57 | -0.41 | -0.41 | -0.44 | -0.36 | 0.29 | -0.30 | -0.48 | -0.52 | -0.46 | -0.42 | -0.27 | -0.47 | -0.64 | -0.39 | 1.00 | 1.00 |  |  |  |  |  |  |  |  |  |  |  |  |  |  |  |  |  |
| PARA_MN Transitory crops | 27 | -0.36 | -0.36 | -0.43 | -0.43 | -0.36 | -0.42 | -0.43 | -0.31 | -0.34 | -0.43 | -0.43 | -0.21 | -0.38 | -0.07 | -0.32 | -0.08 | -0.41 | -0.47 | -0.43 | -0.29 | -0.47 | -0.58 | -0.41 | 0.84 | 0.84 | 0.84 |  |  |  |  |  |  |  |  |  |  |  |  |  |  |  |  |
| CA Bodies of water | 28 | -0.12 | -0.12 | -0.14 | -0.14 | -0.12 | -0.14 | -0.14 | -0.10 | -0.37 | -0.14 | -0.14 | -0.32 | -0.12 | -0.08 | -0.10 | 0.04 | -0.31 | -0.18 | -0.17 | -0.10 | -0.19 | -0.32 | -0.14 | 0.49 | 0.49 | 0.49 | 0.24 |  |  |  |  |  |  |  |  |  |  |  |  |  |  |  |
| PLAND Bodies of water | 29 | -0.12 | -0.12 | -0.14 | -0.14 | -0.12 | -0.14 | -0.14 | -0.10 | -0.37 | -0.14 | -0.14 | -0.32 | -0.12 | -0.08 | -0.10 | 0.04 | -0.31 | -0.18 | -0.17 | -0.10 | -0.19 | -0.32 | -0.14 | 0.49 | 0.49 | 0.49 | 0.24 | 1.00 |  |  |  |  |  |  |  |  |  |  |  |  |  |  |
| NP Bodies of water | 30 | -0.12 | -0.12 | -0.14 | -0.14 | -0.12 | -0.14 | -0.14 | -0.10 | -0.37 | -0.14 | -0.14 | -0.32 | -0.12 | -0.08 | -0.10 | 0.04 | -0.31 | -0.18 | -0.17 | -0.10 | -0.19 | -0.32 | -0.14 | 0.49 | 0.49 | 0.49 | 0.24 | 1.00 | 1.00 |  |  |  |  |  |  |  |  |  |  |  |  |  |
| PD Bodies of water | 31 | -0.12 | -0.12 | -0.14 | -0.14 | -0.12 | -0.14 | -0.14 | -0.10 | -0.37 | -0.14 | -0.14 | -0.32 | -0.12 | -0.08 | -0.10 | 0.04 | -0.31 | -0.18 | -0.17 | -0.10 | -0.19 | -0.32 | -0.14 | 0.49 | 0.49 | 0.49 | 0.24 | 1.00 | 1.00 | 1.00 |  |  |  |  |  |  |  |  |  |  |  |  |
| AREA_MN Bodies of water | 32 | -0.12 | -0.12 | -0.14 | -0.14 | -0.12 | -0.14 | -0.14 | -0.10 | -0.37 | -0.14 | -0.14 | -0.32 | -0.12 | -0.08 | -0.10 | 0.04 | -0.31 | -0.18 | -0.17 | -0.10 | -0.19 | -0.32 | -0.14 | 0.49 | 0.49 | 0.49 | 0.24 | 1.00 | 1.00 | 1.00 | 1.00 |  |  |  |  |  |  |  |  |  |  |  |
| GYRATE_MN Bodies of water | 33 | -0.12 | -0.12 | -0.14 | -0.14 | -0.12 | -0.14 | -0.14 | -0.10 | -0.37 | -0.14 | -0.14 | -0.32 | -0.12 | -0.08 | -0.10 | 0.04 | -0.31 | -0.18 | -0.17 | -0.10 | -0.19 | -0.32 | -0.14 | 0.49 | 0.49 | 0.49 | 0.24 | 1.00 | 1.00 | 1.00 | 1.00 | 1.00 |  |  |  |  |  |  |  |  |  |  |
| PARA_MN Bodies of water | 34 | -0.12 | -0.12 | -0.14 | -0.14 | -0.12 | -0.14 | -0.14 | -0.10 | -0.37 | -0.14 | -0.14 | -0.32 | -0.12 | -0.08 | -0.10 | 0.04 | -0.31 | -0.18 | -0.17 | -0.10 | -0.19 | -0.32 | -0.14 | 0.49 | 0.49 | 0.49 | 0.24 | 1.00 | 1.00 | 1.00 | 1.00 | 1.00 | 1.00 |  |  |  |  |  |  |  |  |  |
| CA Permanent crops | 35 | -0.12 | -0.12 | -0.14 | -0.14 | -0.12 | -0.14 | -0.14 | -0.10 | -0.09 | -0.14 | -0.14 | -0.05 | -0.12 | -0.10 | -0.10 | 0.07 | -0.15 | -0.17 | -0.16 | -0.10 | 0.58 | 0.21 | -0.14 | -0.27 | -0.27 | -0.27 | -0.29 | -0.10 | -0.10 | -0.10 | -0.10 | -0.10 | -0.10 | -0.10 |  |  |  |  |  |  |  |  |
| PLAND Permanent crops | 36 | -0.12 | -0.12 | -0.14 | -0.14 | -0.12 | -0.14 | -0.14 | -0.10 | -0.09 | -0.14 | -0.14 | -0.05 | -0.12 | -0.10 | -0.10 | 0.07 | -0.15 | -0.17 | -0.16 | -0.10 | 0.58 | 0.21 | -0.14 | -0.27 | -0.27 | -0.27 | -0.29 | -0.10 | -0.10 | -0.10 | -0.10 | -0.10 | -0.10 | -0.10 | 1.00 |  |  |  |  |  |  |  |
| NP Permanent crops | 37 | -0.12 | -0.12 | -0.14 | -0.14 | -0.12 | -0.14 | -0.14 | -0.10 | -0.09 | -0.14 | -0.14 | -0.05 | -0.12 | -0.10 | -0.10 | 0.07 | -0.15 | -0.17 | -0.16 | -0.10 | 0.58 | 0.21 | -0.14 | -0.27 | -0.27 | -0.27 | -0.29 | -0.10 | -0.10 | -0.10 | -0.10 | -0.10 | -0.10 | -0.10 | 1.00 | 1.00 |  |  |  |  |  |  |
| PD Permanent crops | 38 | -0.12 | -0.12 | -0.14 | -0.14 | -0.12 | -0.14 | -0.14 | -0.10 | -0.09 | -0.14 | -0.14 | -0.05 | -0.12 | -0.10 | -0.10 | 0.07 | -0.15 | -0.17 | -0.16 | -0.10 | 0.58 | 0.21 | -0.14 | -0.27 | -0.27 | -0.27 | -0.29 | -0.10 | -0.10 | -0.10 | -0.10 | -0.10 | -0.10 | -0.10 | 1.00 | 1.00 | 1.00 |  |  |  |  |  |
| AREA_MN Permanent crops | 39 | -0.12 | -0.12 | -0.14 | -0.14 | -0.12 | -0.14 | -0.14 | -0.10 | -0.09 | -0.14 | -0.14 | -0.05 | -0.12 | -0.10 | -0.10 | 0.07 | -0.15 | -0.17 | -0.16 | -0.10 | 0.58 | 0.21 | -0.14 | -0.27 | -0.27 | -0.27 | -0.29 | -0.10 | -0.10 | -0.10 | -0.10 | -0.10 | -0.10 | -0.10 | 1.00 | 1.00 | 1.00 | 1.00 |  |  |  |  |
| AREA_SD Permanent crops | 40 | -0.12 | -0.12 | -0.14 | -0.14 | -0.12 | -0.14 | -0.14 | -0.10 | -0.09 | -0.14 | -0.14 | -0.05 | -0.12 | -0.10 | -0.10 | 0.07 | -0.15 | -0.17 | -0.16 | -0.10 | 0.58 | 0.21 | -0.14 | -0.27 | -0.27 | -0.27 | -0.29 | -0.10 | -0.10 | -0.10 | -0.10 | -0.10 | -0.10 | -0.10 | 1.00 | 1.00 | 1.00 | 1.00 | 1.00 |  |  |  |
| PARA_MN Permanent crops | 41 | -0.12 | -0.12 | -0.14 | -0.14 | -0.12 | -0.14 | -0.14 | -0.10 | -0.09 | -0.14 | -0.14 | -0.05 | -0.12 | -0.10 | -0.10 | 0.07 | -0.15 | -0.17 | -0.16 | -0.10 | 0.58 | 0.21 | -0.14 | -0.27 | -0.27 | -0.27 | -0.29 | -0.10 | -0.10 | -0.10 | -0.10 | -0.10 | -0.10 | -0.10 | 1.00 | 1.00 | 1.00 | 1.00 | 1.00 | 1.00 |  |  |
| PARA_SD Permanent crops | 42 | -0.12 | -0.12 | -0.14 | -0.14 | -0.12 | -0.14 | -0.14 | -0.10 | -0.09 | -0.14 | -0.14 | -0.05 | -0.12 | -0.10 | -0.10 | 0.07 | -0.15 | -0.17 | -0.16 | -0.10 | 0.58 | 0.21 | -0.14 | -0.27 | -0.27 | -0.27 | -0.29 | -0.10 | -0.10 | -0.10 | -0.10 | -0.10 | -0.10 | -0.10 | 1.00 | 1.00 | 1.00 | 1.00 | 1.00 | 1.00 | 1.00 |  |
| MESH Permanent crops | 43 | -0.12 | -0.12 | -0.14 | -0.14 | -0.12 | -0.14 | -0.14 | -0.10 | -0.09 | -0.14 | -0.14 | -0.05 | -0.12 | -0.10 | -0.10 | 0.07 | -0.15 | -0.17 | -0.16 | -0.10 | 0.58 | 0.21 | -0.14 | -0.27 | -0.27 | -0.27 | -0.29 | -0.10 | -0.10 | -0.10 | -0.10 | -0.10 | -0.10 | -0.10 | 1.00 | 1.00 | 1.00 | 1.00 | 1.00 | 1.00 | 1.00 | 1.00 |

**Appendix S3:**

**Table S2**. Spearman correlation between 16 landscape metrics for each land class at 500 m area of influence. We found 10 independent metrics at four classes: Pastures (CA, PD, AREA_MN and PARA_SD), Forest (PLAND, NP and PD), Bodies of water (CA and PLAND) and AREA_MN of transitory crops. The link for downloading the spreadsheet online is: https://docs.google.com/spreadsheets/d/1N3pfuOvuHY8I6MCeoah7X7CsHO6J4bsD6dAkwXfmwFI/edit?usp=sharing

| **500 m buffer** | **Metric ID** | 1 | 2 | 3 | 4 | 5 | 6 | 7 | 8 | 9 | 10 | 11 | 12 | 13 | 14 | 15 | 16 | 17 | 18 | 19 | 20 | 21 | 22 | 23 | 24 | 25 | 26 | 27 | 28 | 29 | 30 | 31 | 32 | 33 | 34 | 35 | 36 | 37 | 38 | 39 | 40 | 41 | 42 | 43 | 44 | 45 | 46 | 47 | 48 | 49 | 50 | 51 | 52 | 53 | 54 | 55 | 56 | 57 | 58 | 59 | 60 | 6162 63 64 65 66 67 68 69 70 71 72 73 74 75 76 77 78 79 80 81 82 83 84 |
| --- | --- | --- | --- | --- | --- | --- | --- | --- | --- | --- | --- | --- | --- | --- | --- | --- | --- | --- | --- | --- | --- | --- | --- | --- | --- | --- | --- | --- | --- | --- | --- | --- | --- | --- | --- | --- | --- | --- | --- | --- | --- | --- | --- | --- | --- | --- | --- | --- | --- | --- | --- | --- | --- | --- | --- | --- | --- | --- | --- | --- | --- | --- |
| CA Pastures | 1 | 1.00 |  |  |  |  |  |  |  |  |  |  |  |  |  |  |  |  |  |  |  |  |  |  |  |  |  |  |  |  |  |  |  |  |  |  |  |  |  |  |  |  |  |  |  |  |  |  |  |  |  |  |  |  |  |  |  |  |  |  |  |  |
| PLAND Pastures | 2 | 1.00 | 1.00 |  |  |  |  |  |  |  |  |  |  |  |  |  |  |  |  |  |  |  |  |  |  |  |  |  |  |  |  |  |  |  |  |  |  |  |  |  |  |  |  |  |  |  |  |  |  |  |  |  |  |  |  |  |  |  |  |  |  |  |
| NP Pastures | 3 | 0.29 | 0.29 | 1.00 |  |  |  |  |  |  |  |  |  |  |  |  |  |  |  |  |  |  |  |  |  |  |  |  |  |  |  |  |  |  |  |  |  |  |  |  |  |  |  |  |  |  |  |  |  |  |  |  |  |  |  |  |  |  |  |  |  |  |
| PD Pastures | 4 | 0.31 | 0.31 | 0.90 | 1.00 |  |  |  |  |  |  |  |  |  |  |  |  |  |  |  |  |  |  |  |  |  |  |  |  |  |  |  |  |  |  |  |  |  |  |  |  |  |  |  |  |  |  |  |  |  |  |  |  |  |  |  |  |  |  |  |  |  |
| AREA_MN Pastures | 5 | 0.76 | 0.76 | -0.32 | -0.21 | 1.00 |  |  |  |  |  |  |  |  |  |  |  |  |  |  |  |  |  |  |  |  |  |  |  |  |  |  |  |  |  |  |  |  |  |  |  |  |  |  |  |  |  |  |  |  |  |  |  |  |  |  |  |  |  |  |  |  |
| AREA_SD Pastures | 6 | 0.63 | 0.63 | 0.83 | 0.75 | 0.06 | 1.00 |  |  |  |  |  |  |  |  |  |  |  |  |  |  |  |  |  |  |  |  |  |  |  |  |  |  |  |  |  |  |  |  |  |  |  |  |  |  |  |  |  |  |  |  |  |  |  |  |  |  |  |  |  |  |  |
| GYRATE_MN Pastures | 7 | 0.28 | 0.28 | -0.69 | -0.53 | 0.75 | -0.44 | 1.00 |  |  |  |  |  |  |  |  |  |  |  |  |  |  |  |  |  |  |  |  |  |  |  |  |  |  |  |  |  |  |  |  |  |  |  |  |  |  |  |  |  |  |  |  |  |  |  |  |  |  |  |  |  |  |
| GYRATE_SD Pastures | 8 | 0.49 | 0.49 | 0.83 | 0.71 | -0.06 | 0.93 | -0.50 | 1.00 |  |  |  |  |  |  |  |  |  |  |  |  |  |  |  |  |  |  |  |  |  |  |  |  |  |  |  |  |  |  |  |  |  |  |  |  |  |  |  |  |  |  |  |  |  |  |  |  |  |  |  |  |  |
| PARA_MN Pastures | 9 | -0.02 | -0.02 | 0.72 | 0.55 | -0.51 | 0.63 | -0.78 | 0.78 | 1.00 |  |  |  |  |  |  |  |  |  |  |  |  |  |  |  |  |  |  |  |  |  |  |  |  |  |  |  |  |  |  |  |  |  |  |  |  |  |  |  |  |  |  |  |  |  |  |  |  |  |  |  |  |
| PARA_SD Pastures | 10 | 0.49 | 0.49 | 0.86 | 0.69 | -0.08 | 0.92 | -0.56 | 0.98 | 0.80 | 1.00 |  |  |  |  |  |  |  |  |  |  |  |  |  |  |  |  |  |  |  |  |  |  |  |  |  |  |  |  |  |  |  |  |  |  |  |  |  |  |  |  |  |  |  |  |  |  |  |  |  |  |  |
| CONTIG_MN Pastures | 11 | -0.04 | -0.04 | -0.72 | -0.58 | 0.47 | -0.66 | 0.73 | -0.82 | -0.99 | -0.82 | 1.00 |  |  |  |  |  |  |  |  |  |  |  |  |  |  |  |  |  |  |  |  |  |  |  |  |  |  |  |  |  |  |  |  |  |  |  |  |  |  |  |  |  |  |  |  |  |  |  |  |  |  |
| CONTIG_SD Pastures | 12 | 0.49 | 0.49 | 0.86 | 0.69 | -0.08 | 0.92 | -0.56 | 0.98 | 0.80 | 1.00 | -0.82 | 1.00 |  |  |  |  |  |  |  |  |  |  |  |  |  |  |  |  |  |  |  |  |  |  |  |  |  |  |  |  |  |  |  |  |  |  |  |  |  |  |  |  |  |  |  |  |  |  |  |  |  |
| COHESION Pastures | 13 | 0.81 | 0.81 | -0.23 | -0.14 | 0.97 | 0.21 | 0.67 | 0.09 | -0.36 | 0.06 | 0.30 | 0.06 | 1.00 |  |  |  |  |  |  |  |  |  |  |  |  |  |  |  |  |  |  |  |  |  |  |  |  |  |  |  |  |  |  |  |  |  |  |  |  |  |  |  |  |  |  |  |  |  |  |  |  |
| MESH Pastures | 14 | 0.95 | 0.95 | 0.13 | 0.18 | 0.86 | 0.52 | 0.41 | 0.39 | -0.13 | 0.37 | 0.06 | 0.37 | 0.92 | 1.00 |  |  |  |  |  |  |  |  |  |  |  |  |  |  |  |  |  |  |  |  |  |  |  |  |  |  |  |  |  |  |  |  |  |  |  |  |  |  |  |  |  |  |  |  |  |  |  |
| CA Native forest | 15 | -0.18 | -0.18 | 0.45 | 0.30 | -0.42 | -0.08 | -0.14 | -0.15 | -0.11 | -0.05 | 0.17 | -0.05 | -0.49 | -0.26 | 1.00 |  |  |  |  |  |  |  |  |  |  |  |  |  |  |  |  |  |  |  |  |  |  |  |  |  |  |  |  |  |  |  |  |  |  |  |  |  |  |  |  |  |  |  |  |  |  |
| PLAND Native forest | 16 | -0.18 | -0.18 | 0.45 | 0.30 | -0.42 | -0.08 | -0.14 | -0.15 | -0.11 | -0.05 | 0.17 | -0.05 | -0.49 | -0.26 | 1.00 | 1.00 |  |  |  |  |  |  |  |  |  |  |  |  |  |  |  |  |  |  |  |  |  |  |  |  |  |  |  |  |  |  |  |  |  |  |  |  |  |  |  |  |  |  |  |  |  |
| NP Native forest | 17 | -0.09 | -0.09 | 0.23 | 0.30 | -0.15 | 0.26 | -0.26 | 0.14 | -0.01 | 0.02 | -0.01 | 0.02 | 0.02 | 0.02 | -0.05 | -0.05 | 1.00 |  |  |  |  |  |  |  |  |  |  |  |  |  |  |  |  |  |  |  |  |  |  |  |  |  |  |  |  |  |  |  |  |  |  |  |  |  |  |  |  |  |  |  |  |
| PD Native forest | 18 | 0.20 | 0.20 | 0.45 | 0.64 | 0.09 | 0.42 | -0.34 | 0.17 | -0.02 | 0.12 | 0.00 | 0.12 | 0.15 | 0.25 | 0.12 | 0.12 | 0.73 | 1.00 |  |  |  |  |  |  |  |  |  |  |  |  |  |  |  |  |  |  |  |  |  |  |  |  |  |  |  |  |  |  |  |  |  |  |  |  |  |  |  |  |  |  |  |
| AREA_MN Native forest | 19 | -0.12 | -0.12 | 0.19 | 0.07 | -0.28 | -0.24 | 0.08 | -0.22 | -0.10 | -0.08 | 0.14 | -0.08 | -0.41 | -0.23 | 0.81 | 0.81 | -0.59 | -0.34 | 1.00 |  |  |  |  |  |  |  |  |  |  |  |  |  |  |  |  |  |  |  |  |  |  |  |  |  |  |  |  |  |  |  |  |  |  |  |  |  |  |  |  |  |  |
| AREA_SD Native forest | 20 | -0.09 | -0.09 | 0.23 | 0.30 | -0.15 | 0.26 | -0.26 | 0.14 | -0.01 | 0.02 | -0.01 | 0.02 | 0.02 | 0.02 | -0.05 | -0.05 | 1.00 | 0.73 | -0.59 | 1.00 |  |  |  |  |  |  |  |  |  |  |  |  |  |  |  |  |  |  |  |  |  |  |  |  |  |  |  |  |  |  |  |  |  |  |  |  |  |  |  |  |  |
| GYRATE_MN Native forest | 21 | -0.10 | -0.10 | 0.26 | 0.11 | -0.31 | -0.19 | -0.01 | -0.12 | -0.02 | 0.02 | 0.07 | 0.02 | -0.44 | -0.22 | 0.79 | 0.79 | -0.59 | -0.36 | 0.98 | -0.59 | 1.00 |  |  |  |  |  |  |  |  |  |  |  |  |  |  |  |  |  |  |  |  |  |  |  |  |  |  |  |  |  |  |  |  |  |  |  |  |  |  |  |  |
| GYRATE_SD Native forest | 22 | -0.09 | -0.09 | 0.23 | 0.30 | -0.15 | 0.26 | -0.26 | 0.14 | -0.01 | 0.02 | -0.01 | 0.02 | 0.02 | 0.02 | -0.05 | -0.05 | 1.00 | 0.73 | -0.59 | 1.00 | -0.59 | 1.00 |  |  |  |  |  |  |  |  |  |  |  |  |  |  |  |  |  |  |  |  |  |  |  |  |  |  |  |  |  |  |  |  |  |  |  |  |  |  |  |
| PARA_MN Native forest | 23 | 0.06 | 0.06 | 0.07 | 0.17 | 0.11 | 0.34 | -0.30 | 0.41 | 0.41 | 0.30 | -0.45 | 0.30 | 0.26 | 0.19 | -0.66 | -0.66 | 0.59 | 0.48 | -0.87 | 0.59 | -0.85 | 0.59 | 1.00 |  |  |  |  |  |  |  |  |  |  |  |  |  |  |  |  |  |  |  |  |  |  |  |  |  |  |  |  |  |  |  |  |  |  |  |  |  |  |
| PARA_SD Native forest | 24 | -0.09 | -0.09 | 0.23 | 0.30 | -0.15 | 0.26 | -0.26 | 0.14 | -0.01 | 0.02 | -0.01 | 0.02 | 0.02 | 0.02 | -0.05 | -0.05 | 1.00 | 0.73 | -0.59 | 1.00 | -0.59 | 1.00 | 0.59 | 1.00 |  |  |  |  |  |  |  |  |  |  |  |  |  |  |  |  |  |  |  |  |  |  |  |  |  |  |  |  |  |  |  |  |  |  |  |  |  |
| CONTIG_MN Native forest | 25 | -0.03 | -0.03 | 0.06 | -0.04 | -0.15 | -0.27 | 0.20 | -0.34 | -0.32 | -0.22 | 0.36 | -0.22 | -0.29 | -0.17 | 0.74 | 0.74 | -0.59 | -0.39 | 0.93 | -0.59 | 0.92 | -0.59 | -0.98 | -0.59 | 1.00 |  |  |  |  |  |  |  |  |  |  |  |  |  |  |  |  |  |  |  |  |  |  |  |  |  |  |  |  |  |  |  |  |  |  |  |  |
| CONTIG_SD Native forest | 26 | -0.09 | -0.09 | 0.23 | 0.30 | -0.15 | 0.26 | -0.26 | 0.14 | -0.01 | 0.02 | -0.01 | 0.02 | 0.02 | 0.02 | -0.05 | -0.05 | 1.00 | 0.73 | -0.59 | 1.00 | -0.59 | 1.00 | 0.59 | 1.00 | -0.59 | 1.00 |  |  |  |  |  |  |  |  |  |  |  |  |  |  |  |  |  |  |  |  |  |  |  |  |  |  |  |  |  |  |  |  |  |  |  |
| COHESION Native forest | 27 | -0.13 | -0.13 | 0.26 | 0.16 | -0.33 | -0.19 | 0.09 | -0.19 | -0.15 | -0.08 | 0.17 | -0.08 | -0.42 | -0.22 | 0.89 | 0.89 | -0.38 | -0.22 | 0.96 | -0.38 | 0.95 | -0.38 | -0.83 | -0.38 | 0.90 | -0.38 | 1.00 |  |  |  |  |  |  |  |  |  |  |  |  |  |  |  |  |  |  |  |  |  |  |  |  |  |  |  |  |  |  |  |  |  |  |
| MESH Native forest | 28 | -0.13 | -0.13 | 0.37 | 0.26 | -0.35 | -0.10 | 0.02 | -0.12 | -0.10 | -0.02 | 0.13 | -0.02 | -0.42 | -0.21 | 0.95 | 0.95 | -0.23 | -0.06 | 0.91 | -0.23 | 0.89 | -0.23 | -0.73 | -0.23 | 0.81 | -0.23 | 0.98 | 1.00 |  |  |  |  |  |  |  |  |  |  |  |  |  |  |  |  |  |  |  |  |  |  |  |  |  |  |  |  |  |  |  |  |  |
| CA Urban infrastructure | 29 | -1.00 | -1.00 | 0.00 | -1.00 | -1.00 | -1.00 | -1.00 | -1.00 | 1.00 | -1.00 | 1.00 | -1.00 | -1.00 | -1.00 | 1.00 | 1.00 | -1.00 | -1.00 | 1.00 | -1.00 | 1.00 | -1.00 | -1.00 | -1.00 | 1.00 | -1.00 | 1.00 | 1.00 | 1.00 |  |  |  |  |  |  |  |  |  |  |  |  |  |  |  |  |  |  |  |  |  |  |  |  |  |  |  |  |  |  |  |  |
| PLAND Urban infrastructure | 30 | -1.00 | -1.00 | 0.00 | -1.00 | -1.00 | -1.00 | -1.00 | -1.00 | 1.00 | -1.00 | 1.00 | -1.00 | -1.00 | -1.00 | 1.00 | 1.00 | -1.00 | -1.00 | 1.00 | -1.00 | 1.00 | -1.00 | -1.00 | -1.00 | 1.00 | -1.00 | 1.00 | 1.00 | 1.00 | 1.00 |  |  |  |  |  |  |  |  |  |  |  |  |  |  |  |  |  |  |  |  |  |  |  |  |  |  |  |  |  |  |  |
| NP Urban infrastructure | 31 | 0.00 | 0.00 | 0.00 | 0.00 | 0.00 | 0.00 | 0.00 | 0.00 | 0.00 | 0.00 | 0.00 | 0.00 | 0.00 | 0.00 | 0.00 | 0.00 | 0.00 | 0.00 | 0.00 | 0.00 | 0.00 | 0.00 | 0.00 | 0.00 | 0.00 | 0.00 | 0.00 | 0.00 | 0.00 | 0.00 | 1.00 |  |  |  |  |  |  |  |  |  |  |  |  |  |  |  |  |  |  |  |  |  |  |  |  |  |  |  |  |  |  |
| PD Urban infrastructure | 32 | 1.00 | 1.00 | 0.00 | 1.00 | 1.00 | 1.00 | 1.00 | 1.00 | -1.00 | 1.00 | -1.00 | 1.00 | 1.00 | 1.00 | -1.00 | -1.00 | 1.00 | 1.00 | -1.00 | 1.00 | -1.00 | 1.00 | 1.00 | 1.00 | -1.00 | 1.00 | -1.00 | -1.00 | -1.00 | -1.00 | 0.00 | 1.00 |  |  |  |  |  |  |  |  |  |  |  |  |  |  |  |  |  |  |  |  |  |  |  |  |  |  |  |  |  |
| AREA_MN Urban infrastructure | 33 | -1.00 | -1.00 | 0.00 | -1.00 | -1.00 | -1.00 | -1.00 | -1.00 | 1.00 | -1.00 | 1.00 | -1.00 | -1.00 | -1.00 | 1.00 | 1.00 | -1.00 | -1.00 | 1.00 | -1.00 | 1.00 | -1.00 | -1.00 | -1.00 | 1.00 | -1.00 | 1.00 | 1.00 | 1.00 | 1.00 | 0.00 | -1.00 | 1.00 |  |  |  |  |  |  |  |  |  |  |  |  |  |  |  |  |  |  |  |  |  |  |  |  |  |  |  |  |
| AREA_SD Urban infrastructure | 34 | 0.00 | 0.00 | 0.00 | 0.00 | 0.00 | 0.00 | 0.00 | 0.00 | 0.00 | 0.00 | 0.00 | 0.00 | 0.00 | 0.00 | 0.00 | 0.00 | 0.00 | 0.00 | 0.00 | 0.00 | 0.00 | 0.00 | 0.00 | 0.00 | 0.00 | 0.00 | 0.00 | 0.00 | 0.00 | 0.00 | 0.00 | 0.00 | 0.00 | 1.00 |  |  |  |  |  |  |  |  |  |  |  |  |  |  |  |  |  |  |  |  |  |  |  |  |  |  |  |
| GYRATE_MN Urban infrastructure | 35 | -1.00 | -1.00 | 0.00 | -1.00 | -1.00 | -1.00 | -1.00 | -1.00 | 1.00 | -1.00 | 1.00 | -1.00 | -1.00 | -1.00 | 1.00 | 1.00 | -1.00 | -1.00 | 1.00 | -1.00 | 1.00 | -1.00 | -1.00 | -1.00 | 1.00 | -1.00 | 1.00 | 1.00 | 1.00 | 1.00 | 0.00 | -1.00 | 1.00 | 0.00 | 1.00 |  |  |  |  |  |  |  |  |  |  |  |  |  |  |  |  |  |  |  |  |  |  |  |  |  |  |
| GYRATE_SD Urban infrastructure | 36 | 0.00 | 0.00 | 0.00 | 0.00 | 0.00 | 0.00 | 0.00 | 0.00 | 0.00 | 0.00 | 0.00 | 0.00 | 0.00 | 0.00 | 0.00 | 0.00 | 0.00 | 0.00 | 0.00 | 0.00 | 0.00 | 0.00 | 0.00 | 0.00 | 0.00 | 0.00 | 0.00 | 0.00 | 0.00 | 0.00 | 0.00 | 0.00 | 0.00 | 0.00 | 0.00 | 1.00 |  |  |  |  |  |  |  |  |  |  |  |  |  |  |  |  |  |  |  |  |  |  |  |  |  |
| PARA_MN Urban infrastructure | 37 | 1.00 | 1.00 | 0.00 | 1.00 | 1.00 | 1.00 | 1.00 | 1.00 | -1.00 | 1.00 | -1.00 | 1.00 | 1.00 | 1.00 | -1.00 | -1.00 | 1.00 | 1.00 | -1.00 | 1.00 | -1.00 | 1.00 | 1.00 | 1.00 | -1.00 | 1.00 | -1.00 | -1.00 | -1.00 | -1.00 | 0.00 | 1.00 | -1.00 | 0.00 | -1.00 | 0.00 | 1.00 |  |  |  |  |  |  |  |  |  |  |  |  |  |  |  |  |  |  |  |  |  |  |  |  |
| PARA_SD Urban infrastructure | 38 | 0.00 | 0.00 | 0.00 | 0.00 | 0.00 | 0.00 | 0.00 | 0.00 | 0.00 | 0.00 | 0.00 | 0.00 | 0.00 | 0.00 | 0.00 | 0.00 | 0.00 | 0.00 | 0.00 | 0.00 | 0.00 | 0.00 | 0.00 | 0.00 | 0.00 | 0.00 | 0.00 | 0.00 | 0.00 | 0.00 | 0.00 | 0.00 | 0.00 | 0.00 | 0.00 | 0.00 | 0.00 | 1.00 |  |  |  |  |  |  |  |  |  |  |  |  |  |  |  |  |  |  |  |  |  |  |  |
| CONTIG_MN Urban infrastructure | 39 | 1.00 | 1.00 | 0.00 | 1.00 | 1.00 | 1.00 | 1.00 | 1.00 | -1.00 | 1.00 | -1.00 | 1.00 | 1.00 | 1.00 | -1.00 | -1.00 | 1.00 | 1.00 | -1.00 | 1.00 | -1.00 | 1.00 | 1.00 | 1.00 | -1.00 | 1.00 | -1.00 | -1.00 | -1.00 | -1.00 | 0.00 | 1.00 | -1.00 | 0.00 | -1.00 | 0.00 | 1.00 | 0.00 | 1.00 |  |  |  |  |  |  |  |  |  |  |  |  |  |  |  |  |  |  |  |  |  |  |
| CONTIG_SD Urban infrastructure | 40 | 0.00 | 0.00 | 0.00 | 0.00 | 0.00 | 0.00 | 0.00 | 0.00 | 0.00 | 0.00 | 0.00 | 0.00 | 0.00 | 0.00 | 0.00 | 0.00 | 0.00 | 0.00 | 0.00 | 0.00 | 0.00 | 0.00 | 0.00 | 0.00 | 0.00 | 0.00 | 0.00 | 0.00 | 0.00 | 0.00 | 0.00 | 0.00 | 0.00 | 0.00 | 0.00 | 0.00 | 0.00 | 0.00 | 0.00 | 1.00 |  |  |  |  |  |  |  |  |  |  |  |  |  |  |  |  |  |  |  |  |  |
| COHESION Urban infrastructure | 41 | -1.00 | -1.00 | 0.00 | -1.00 | -1.00 | -1.00 | -1.00 | -1.00 | 1.00 | -1.00 | 1.00 | -1.00 | -1.00 | -1.00 | 1.00 | 1.00 | -1.00 | -1.00 | 1.00 | -1.00 | 1.00 | -1.00 | -1.00 | -1.00 | 1.00 | -1.00 | 1.00 | 1.00 | 1.00 | 1.00 | 0.00 | -1.00 | 1.00 | 0.00 | 1.00 | 0.00 | -1.00 | 0.00 | -1.00 | 0.00 | 1.00 |  |  |  |  |  |  |  |  |  |  |  |  |  |  |  |  |  |  |  |  |
| MESH Urban infrastructure | 42 | -1.00 | -1.00 | 0.00 | -1.00 | -1.00 | -1.00 | -1.00 | -1.00 | 1.00 | -1.00 | 1.00 | -1.00 | -1.00 | -1.00 | 1.00 | 1.00 | -1.00 | -1.00 | 1.00 | -1.00 | 1.00 | -1.00 | -1.00 | -1.00 | 1.00 | -1.00 | 1.00 | 1.00 | 1.00 | 1.00 | 0.00 | -1.00 | 1.00 | 0.00 | 1.00 | 0.00 | -1.00 | 0.00 | -1.00 | 0.00 | 1.00 | 1.00 |  |  |  |  |  |  |  |  |  |  |  |  |  |  |  |  |  |  |  |
| CA Bodies of water | 43 | 0.00 | 0.00 | 0.00 | 0.00 | 0.00 | 0.00 | 0.00 | 0.00 | 0.00 | 0.00 | 0.00 | 0.00 | 0.00 | 0.00 | sd | sd | sd | sd | sd | sd | sd | sd | sd | sd | sd | sd | sd | sd | sd | sd | sd | sd | sd | sd | sd | sd | sd | sd | sd | sd | sd | sd | 1.00 |  |  |  |  |  |  |  |  |  |  |  |  |  |  |  |  |  |  |
| PLAND Bodies of water | 44 | 0.00 | 0.00 | 0.00 | 0.00 | 0.00 | 0.00 | 0.00 | 0.00 | 0.00 | 0.00 | 0.00 | 0.00 | 0.00 | 0.00 | sd | sd | sd | sd | sd | sd | sd | sd | sd | sd | sd | sd | sd | sd | sd | sd | sd | sd | sd | sd | sd | sd | sd | sd | sd | sd | sd | sd | 0.00 | 1.00 |  |  |  |  |  |  |  |  |  |  |  |  |  |  |  |  |  |
| NP Bodies of water | 45 | 0.00 | 0.00 | 0.00 | 0.00 | 0.00 | 0.00 | 0.00 | 0.00 | 0.00 | 0.00 | 0.00 | 0.00 | 0.00 | 0.00 | sd | sd | sd | sd | sd | sd | sd | sd | sd | sd | sd | sd | sd | sd | sd | sd | sd | sd | sd | sd | sd | sd | sd | sd | sd | sd | sd | sd | 0.00 | 0.00 | 1.00 |  |  |  |  |  |  |  |  |  |  |  |  |  |  |  |  |
| PD Bodies of water | 46 | 0.00 | 0.00 | 0.00 | 0.00 | 0.00 | 0.00 | 0.00 | 0.00 | 0.00 | 0.00 | 0.00 | 0.00 | 0.00 | 0.00 | sd | sd | sd | sd | sd | sd | sd | sd | sd | sd | sd | sd | sd | sd | sd | sd | sd | sd | sd | sd | sd | sd | sd | sd | sd | sd | sd | sd | 0.00 | 0.00 | 0.00 | 1.00 |  |  |  |  |  |  |  |  |  |  |  |  |  |  |  |
| AREA_MN Bodies of water | 47 | 0.00 | 0.00 | 0.00 | 0.00 | 0.00 | 0.00 | 0.00 | 0.00 | 0.00 | 0.00 | 0.00 | 0.00 | 0.00 | 0.00 | sd | sd | sd | sd | sd | sd | sd | sd | sd | sd | sd | sd | sd | sd | sd | sd | sd | sd | sd | sd | sd | sd | sd | sd | sd | sd | sd | sd | 0.00 | 0.00 | 0.00 | 0.00 | 1.00 |  |  |  |  |  |  |  |  |  |  |  |  |  |  |
| AREA_SD Bodies of water | 48 | 0.00 | 0.00 | 0.00 | 0.00 | 0.00 | 0.00 | 0.00 | 0.00 | 0.00 | 0.00 | 0.00 | 0.00 | 0.00 | 0.00 | sd | sd | sd | sd | sd | sd | sd | sd | sd | sd | sd | sd | sd | sd | sd | sd | sd | sd | sd | sd | sd | sd | sd | sd | sd | sd | sd | sd | 0.00 | 0.00 | 0.00 | 0.00 | 0.00 | 1.00 |  |  |  |  |  |  |  |  |  |  |  |  |  |
| GYRATE_MN Bodies of water | 49 | 0.00 | 0.00 | 0.00 | 0.00 | 0.00 | 0.00 | 0.00 | 0.00 | 0.00 | 0.00 | 0.00 | 0.00 | 0.00 | 0.00 | sd | sd | sd | sd | sd | sd | sd | sd | sd | sd | sd | sd | sd | sd | sd | sd | sd | sd | sd | sd | sd | sd | sd | sd | sd | sd | sd | sd | 0.00 | 0.00 | 0.00 | 0.00 | 0.00 | 0.00 | 1.00 |  |  |  |  |  |  |  |  |  |  |  |  |
| GYRATE_SD Bodies of water | 50 | 0.00 | 0.00 | 0.00 | 0.00 | 0.00 | 0.00 | 0.00 | 0.00 | 0.00 | 0.00 | 0.00 | 0.00 | 0.00 | 0.00 | sd | sd | sd | sd | sd | sd | sd | sd | sd | sd | sd | sd | sd | sd | sd | sd | sd | sd | sd | sd | sd | sd | sd | sd | sd | sd | sd | sd | 0.00 | 0.00 | 0.00 | 0.00 | 0.00 | 0.00 | 0.00 | 1.00 |  |  |  |  |  |  |  |  |  |  |  |
| PARA_MN Bodies of water | 51 | 0.00 | 0.00 | 0.00 | 0.00 | 0.00 | 0.00 | 0.00 | 0.00 | 0.00 | 0.00 | 0.00 | 0.00 | 0.00 | 0.00 | sd | sd | sd | sd | sd | sd | sd | sd | sd | sd | sd | sd | sd | sd | sd | sd | sd | sd | sd | sd | sd | sd | sd | sd | sd | sd | sd | sd | 0.00 | 0.00 | 0.00 | 0.00 | 0.00 | 0.00 | 0.00 | 0.00 | 1.00 |  |  |  |  |  |  |  |  |  |  |
| PARA_SD Bodies of water | 52 | 0.00 | 0.00 | 0.00 | 0.00 | 0.00 | 0.00 | 0.00 | 0.00 | 0.00 | 0.00 | 0.00 | 0.00 | 0.00 | 0.00 | sd | sd | sd | sd | sd | sd | sd | sd | sd | sd | sd | sd | sd | sd | sd | sd | sd | sd | sd | sd | sd | sd | sd | sd | sd | sd | sd | sd | 0.00 | 0.00 | 0.00 | 0.00 | 0.00 | 0.00 | 0.00 | 0.00 | 0.00 | 1.00 |  |  |  |  |  |  |  |  |  |
| CONTIG_MN Bodies of water | 53 | 0.00 | 0.00 | 0.00 | 0.00 | 0.00 | 0.00 | 0.00 | 0.00 | 0.00 | 0.00 | 0.00 | 0.00 | 0.00 | 0.00 | sd | sd | sd | sd | sd | sd | sd | sd | sd | sd | sd | sd | sd | sd | sd | sd | sd | sd | sd | sd | sd | sd | sd | sd | sd | sd | sd | sd | 0.00 | 0.00 | 0.00 | 0.00 | 0.00 | 0.00 | 0.00 | 0.00 | 0.00 | 0.00 | 1.00 |  |  |  |  |  |  |  |  |
| CONTIG_SD Bodies of water. | 54 | 0.00 | 0.00 | 0.00 | 0.00 | 0.00 | 0.00 | 0.00 | 0.00 | 0.00 | 0.00 | 0.00 | 0.00 | 0.00 | 0.00 | sd | sd | sd | sd | sd | sd | sd | sd | sd | sd | sd | sd | sd | sd | sd | sd | sd | sd | sd | sd | sd | sd | sd | sd | sd | sd | sd | sd | 0.00 | 0.00 | 0.00 | 0.00 | 0.00 | 0.00 | 0.00 | 0.00 | 0.00 | 0.00 | 0.00 | 1.00 |  |  |  |  |  |  |  |
| COHESION Bodies of water.. | 55 | 0.00 | 0.00 | 0.00 | 0.00 | 0.00 | 0.00 | 0.00 | 0.00 | 0.00 | 0.00 | 0.00 | 0.00 | 0.00 | 0.00 | sd | sd | sd | sd | sd | sd | sd | sd | sd | sd | sd | sd | sd | sd | sd | sd | sd | sd | sd | sd | sd | sd | sd | sd | sd | sd | sd | sd | 0.00 | 0.00 | 0.00 | 0.00 | 0.00 | 0.00 | 0.00 | 0.00 | 0.00 | 0.00 | 0.00 | 0.00 | 1.00 |  |  |  |  |  |  |
| MESH Bodies of water | 56 | 0.00 | 0.00 | 0.00 | 0.00 | 0.00 | 0.00 | 0.00 | 0.00 | 0.00 | 0.00 | 0.00 | 0.00 | 0.00 | 0.00 | sd | sd | sd | sd | sd | sd | sd | sd | sd | sd | sd | sd | sd | sd | sd | sd | sd | sd | sd | sd | sd | sd | sd | sd | sd | sd | sd | sd | 0.00 | 0.00 | 0.00 | 0.00 | 0.00 | 0.00 | 0.00 | 0.00 | 0.00 | 0.00 | 0.00 | 0.00 | 0.00 | 1.00 |  |  |  |  |  |
| CA Transitory crops | 57 | -1.00 | -1.00 | 0.53 | 0.15 | -1.00 | 0.53 | -0.83 | 0.53 | 0.97 | 0.53 | -0.96 | 0.53 | -1.00 | -1.00 | 0.39 | 0.39 | 0.00 | 0.09 | 0.39 | 0.00 | 0.39 | 0.00 | 0.09 | 0.00 | -0.09 | 0.00 | 0.39 | 0.39 | sd | sd | sd | sd | sd | sd | sd | sd | sd | sd | sd | sd | sd | sd | 0.00 | 0.00 | 0.00 | 0.00 | 0.00 | 0.00 | 0.00 | 0.00 | 0.00 | 0.00 | 0.00 | 0.00 | 0.00 | 0.00 | 1.00 |  |  |  |  |
| PLAND Transitory crops | 58 | -1.00 | -1.00 | 0.53 | 0.15 | -1.00 | 0.53 | -0.83 | 0.53 | 0.97 | 0.53 | -0.96 | 0.53 | -1.00 | -1.00 | 0.39 | 0.39 | 0.00 | 0.09 | 0.39 | 0.00 | 0.39 | 0.00 | 0.09 | 0.00 | -0.09 | 0.00 | 0.39 | 0.39 | sd | sd | sd | sd | sd | sd | sd | sd | sd | sd | sd | sd | sd | sd | 0.00 | 0.00 | 0.00 | 0.00 | 0.00 | 0.00 | 0.00 | 0.00 | 0.00 | 0.00 | 0.00 | 0.00 | 0.00 | 0.00 | 1.00 | 1.00 |  |  |  |
| NP Transitory crops | 59 | -0.60 | -0.60 | 0.62 | 0.24 | -0.60 | 0.62 | -0.24 | 0.62 | 0.72 | 0.62 | -0.72 | 0.62 | -0.60 | -0.60 | 0.85 | 0.85 | 0.00 | 0.00 | 0.85 | 0.00 | 0.85 | 0.00 | 0.00 | 0.00 | 0.00 | 0.00 | 0.85 | 0.85 | sd | sd | sd | sd | sd | sd | sd | sd | sd | sd | sd | sd | sd | sd | 0.00 | 0.00 | 0.00 | 0.00 | 0.00 | 0.00 | 0.00 | 0.00 | 0.00 | 0.00 | 0.00 | 0.00 | 0.00 | 0.00 | 0.60 | 0.60 | 1.00 |  |  |
| PD Transitory crops | 60 | -0.46 | -0.46 | 0.68 | 0.67 | -0.46 | 0.68 | -0.28 | 0.68 | 0.58 | 0.68 | -0.57 | 0.68 | -0.46 | -0.46 | 0.45 | 0.45 | 0.00 | 0.52 | 0.45 | 0.00 | 0.45 | 0.00 | 0.52 | 0.00 | -0.52 | 0.00 | 0.45 | 0.45 | sd | sd | sd | sd | sd | sd | sd | sd | sd | sd | sd | sd | sd | sd | 0.00 | 0.00 | 0.00 | 0.00 | 0.00 | 0.00 | 0.00 | 0.00 | 0.00 | 0.00 | 0.00 | 0.00 | 0.00 | 0.00 | 0.46 | 0.46 | 0.85 | 1.00 |  |
| AREA_MN Transitory crops | 61 | 0.11 | 0.11 | -0.38 | -0.22 | 0.11 | -0.38 | -0.28 | -0.38 | -0.25 | -0.38 | 0.26 | -0.38 | 0.11 | 0.11 | -0.64 | -0.64 | 0.00 | -0.09 | -0.64 | 0.00 | -0.64 | 0.00 | -0.09 | 0.00 | 0.09 | 0.00 | -0.64 | -0.64 | sd | sd | sd | sd | sd | sd | sd | sd | sd | sd | sd | sd | sd | sd | 0.00 | 0.00 | 0.00 | 0.00 | 0.00 | 0.00 | 0.00 | 0.00 | 0.00 | 0.00 | 0.00 | 0.00 | 0.00 | 0.00 | -0.11 | -0.11 | -0.84 | -0.77 | 1.00 |
| AREA_SD Transitory crops | 62 | -0.63 | -0.63 | 0.78 | 0.39 | -0.63 | 0.78 | -0.34 | 0.78 | 0.77 | 0.78 | -0.77 | 0.78 | -0.63 | -0.63 | 0.85 | 0.85 | 0.00 | 0.00 | 0.85 | 0.00 | 0.85 | 0.00 | 0.00 | 0.00 | 0.00 | 0.00 | 0.85 | 0.85 | sd | sd | sd | sd | sd | sd | sd | sd | sd | sd | sd | sd | sd | sd | 0.00 | 0.00 | 0.00 | 0.00 | 0.00 | 0.00 | 0.00 | 0.00 | 0.00 | 0.00 | 0.00 | 0.00 | 0.00 | 0.00 | 0.63 | 0.63 | 0.97 | 0.87 | -0.781.00 |
| GYRATE_MN Transitory crops | 63 | 0.11 | 0.11 | -0.38 | -0.22 | 0.11 | -0.38 | -0.28 | -0.38 | -0.25 | -0.38 | 0.26 | -0.38 | 0.11 | 0.11 | -0.64 | -0.64 | 0.00 | -0.09 | -0.64 | 0.00 | -0.64 | 0.00 | -0.09 | 0.00 | 0.09 | 0.00 | -0.64 | -0.64 | sd | sd | sd | sd | sd | sd | sd | sd | sd | sd | sd | sd | sd | sd | 0.00 | 0.00 | 0.00 | 0.00 | 0.00 | 0.00 | 0.00 | 0.00 | 0.00 | 0.00 | 0.00 | 0.00 | 0.00 | 0.00 | -0.11 | -0.11 | -0.84 | -0.77 | 1.00-0.78 1.00 |
| GYRATE_SD Transitory crops | 64 | -0.63 | -0.63 | 0.78 | 0.39 | -0.63 | 0.78 | -0.34 | 0.78 | 0.77 | 0.78 | -0.77 | 0.78 | -0.63 | -0.63 | 0.85 | 0.85 | 0.00 | 0.00 | 0.85 | 0.00 | 0.85 | 0.00 | 0.00 | 0.00 | 0.00 | 0.00 | 0.85 | 0.85 | sd | sd | sd | sd | sd | sd | sd | sd | sd | sd | sd | sd | sd | sd | 0.00 | 0.00 | 0.00 | 0.00 | 0.00 | 0.00 | 0.00 | 0.00 | 0.00 | 0.00 | 0.00 | 0.00 | 0.00 | 0.00 | 0.63 | 0.63 | 0.97 | 0.87 | -0.781.00 -0.78 1.00 |
| PARA_MN Transitory crops | 65 | -0.12 | -0.12 | 0.38 | 0.23 | -0.12 | 0.38 | 0.23 | 0.38 | 0.26 | 0.38 | -0.25 | 0.38 | -0.12 | -0.12 | 0.64 | 0.64 | 0.00 | 0.09 | 0.64 | 0.00 | 0.64 | 0.00 | 0.09 | 0.00 | -0.09 | 0.00 | 0.64 | 0.64 | sd | sd | sd | sd | sd | sd | sd | sd | sd | sd | sd | sd | sd | sd | 0.00 | 0.00 | 0.00 | 0.00 | 0.00 | 0.00 | 0.00 | 0.00 | 0.00 | 0.00 | 0.00 | 0.00 | 0.00 | 0.00 | 0.12 | 0.12 | 0.84 | 0.78 | -0.990.78 -0.99 0.78 1.00 |
| PARA_SD Transitory crops | 66 | -0.55 | -0.55 | 0.43 | 0.09 | -0.55 | 0.43 | -0.13 | 0.43 | 0.64 | 0.43 | -0.64 | 0.43 | -0.55 | -0.55 | 0.85 | 0.85 | 0.00 | 0.00 | 0.85 | 0.00 | 0.85 | 0.00 | 0.00 | 0.00 | 0.00 | 0.00 | 0.85 | 0.85 | sd | sd | sd | sd | sd | sd | sd | sd | sd | sd | sd | sd | sd | sd | 0.00 | 0.00 | 0.00 | 0.00 | 0.00 | 0.00 | 0.00 | 0.00 | 0.00 | 0.00 | 0.00 | 0.00 | 0.00 | 0.00 | 0.55 | 0.55 | 0.97 | 0.79 | -0.870.90 -0.87 0.90 0.87 1.00 |
| CONTIG_MN Transitory crops | 67 | 0.12 | 0.12 | -0.38 | -0.23 | 0.12 | -0.38 | -0.23 | -0.38 | -0.26 | -0.38 | 0.25 | -0.38 | 0.12 | 0.12 | -0.64 | -0.64 | 0.00 | -0.09 | -0.64 | 0.00 | -0.64 | 0.00 | -0.09 | 0.00 | 0.09 | 0.00 | -0.64 | -0.64 | sd | sd | sd | sd | sd | sd | sd | sd | sd | sd | sd | sd | sd | sd | 0.00 | 0.00 | 0.00 | 0.00 | 0.00 | 0.00 | 0.00 | 0.00 | 0.00 | 0.00 | 0.00 | 0.00 | 0.00 | 0.00 | -0.12 | -0.12 | -0.84 | -0.78 | 0.99-0.78 0.99 -0.78 -1.00 -0.87 1.00 |
| CONTIG_SD Transitory crops | 68 | -0.55 | -0.55 | 0.43 | 0.09 | -0.55 | 0.43 | -0.13 | 0.43 | 0.64 | 0.43 | -0.64 | 0.43 | -0.55 | -0.55 | 0.85 | 0.85 | 0.00 | 0.00 | 0.85 | 0.00 | 0.85 | 0.00 | 0.00 | 0.00 | 0.00 | 0.00 | 0.85 | 0.85 | sd | sd | sd | sd | sd | sd | sd | sd | sd | sd | sd | sd | sd | sd | 0.00 | 0.00 | 0.00 | 0.00 | 0.00 | 0.00 | 0.00 | 0.00 | 0.00 | 0.00 | 0.00 | 0.00 | 0.00 | 0.00 | 0.55 | 0.55 | 0.97 | 0.79 | -0.870.90 -0.87 0.90 0.87 1.00 -0.87 1.00 |
| COHESION Transitory crops | 69 | -0.19 | -0.19 | -0.08 | -0.05 | -0.19 | -0.08 | -0.57 | -0.08 | 0.08 | -0.08 | -0.07 | -0.08 | -0.19 | -0.19 | -0.64 | -0.64 | 0.00 | -0.09 | -0.64 | 0.00 | -0.64 | 0.00 | -0.09 | 0.00 | 0.09 | 0.00 | -0.64 | -0.64 | sd | sd | sd | sd | sd | sd | sd | sd | sd | sd | sd | sd | sd | sd | 0.00 | 0.00 | 0.00 | 0.00 | 0.00 | 0.00 | 0.00 | 0.00 | 0.00 | 0.00 | 0.00 | 0.00 | 0.00 | 0.00 | 0.19 | 0.19 | -0.60 | -0.53 | 0.93-0.50 0.93 -0.50 -0.92 -0.67 0.92 -0.67 1.00 |
| MESH Transitory crops | 70 | -0.86 | -0.86 | 0.53 | 0.18 | -0.86 | 0.53 | -0.97 | 0.53 | 0.83 | 0.53 | -0.81 | 0.53 | -0.86 | -0.86 | -0.64 | -0.64 | 0.00 | -0.09 | -0.64 | 0.00 | -0.64 | 0.00 | -0.09 | 0.00 | 0.09 | 0.00 | -0.64 | -0.64 | sd | sd | sd | sd | sd | sd | sd | sd | sd | sd | sd | sd | sd | sd | 0.00 | 0.00 | 0.00 | 0.00 | 0.00 | 0.00 | 0.00 | 0.00 | 0.00 | 0.00 | 0.00 | 0.00 | 0.00 | 0.00 | 0.86 | 0.86 | 0.24 | 0.17 | 0.310.34 0.31 0.34 -0.28 0.13 0.28 0.13 0.60 1.00 |
| CA Permanent crops | 71 | 1.00 | 1.00 | 0.00 | 0.00 | 1.00 | 1.00 | 1.00 | 1.00 | 1.00 | 1.00 | -1.00 | 1.00 | 1.00 | 1.00 | -1.00 | -1.00 | -1.00 | -1.00 | 1.00 | -1.00 | 1.00 | -1.00 | -1.00 | -1.00 | 1.00 | -1.00 | 1.00 | 1.00 | 0.00 | 0.00 | 0.00 | 0.00 | 0.00 | 0.00 | 0.00 | 0.00 | 0.00 | 0.00 | 0.00 | 0.00 | 0.00 | 0.00 | sd | sd | sd | sd | sd | sd | sd | sd | sd | sd | sd | sd | sd | sd | sd | sd | sd | sd | sd sd sd sd sd sd sd sd sd sd 1.00 |
| PLAND Permanent crops | 72 | 1.00 | 1.00 | 0.00 | 0.00 | 1.00 | 1.00 | 1.00 | 1.00 | 1.00 | 1.00 | -1.00 | 1.00 | 1.00 | 1.00 | -1.00 | -1.00 | -1.00 | -1.00 | 1.00 | -1.00 | 1.00 | -1.00 | -1.00 | -1.00 | 1.00 | -1.00 | 1.00 | 1.00 | 0.00 | 0.00 | 0.00 | 0.00 | 0.00 | 0.00 | 0.00 | 0.00 | 0.00 | 0.00 | 0.00 | 0.00 | 0.00 | 0.00 | sd | sd | sd | sd | sd | sd | sd | sd | sd | sd | sd | sd | sd | sd | sd | sd | sd | sd | sd sd sd sd sd sd sd sd sd sd 1.00 1.00 |
| NP Permanent crops | 73 | 1.00 | 1.00 | 0.00 | 0.00 | 1.00 | 1.00 | 1.00 | 1.00 | 1.00 | 1.00 | -1.00 | 1.00 | 1.00 | 1.00 | -1.00 | -1.00 | -1.00 | -1.00 | 1.00 | -1.00 | 1.00 | -1.00 | -1.00 | -1.00 | 1.00 | -1.00 | 1.00 | 1.00 | 0.00 | 0.00 | 0.00 | 0.00 | 0.00 | 0.00 | 0.00 | 0.00 | 0.00 | 0.00 | 0.00 | 0.00 | 0.00 | 0.00 | sd | sd | sd | sd | sd | sd | sd | sd | sd | sd | sd | sd | sd | sd | sd | sd | sd | sd | sd sd sd sd sd sd sd sd sd sd 1.00 1.00 1.00 |
| PD Permanent crops | 74 | 1.00 | 1.00 | 0.00 | 0.00 | 1.00 | 1.00 | 1.00 | 1.00 | 1.00 | 1.00 | -1.00 | 1.00 | 1.00 | 1.00 | -1.00 | -1.00 | -1.00 | -1.00 | 1.00 | -1.00 | 1.00 | -1.00 | -1.00 | -1.00 | 1.00 | -1.00 | 1.00 | 1.00 | 0.00 | 0.00 | 0.00 | 0.00 | 0.00 | 0.00 | 0.00 | 0.00 | 0.00 | 0.00 | 0.00 | 0.00 | 0.00 | 0.00 | sd | sd | sd | sd | sd | sd | sd | sd | sd | sd | sd | sd | sd | sd | sd | sd | sd | sd | sd sd sd sd sd sd sd sd sd sd 1.00 1.00 1.00 1.00 |
| AREA_MN Permanent crops | 75 | 1.00 | 1.00 | 0.00 | 0.00 | 1.00 | 1.00 | 1.00 | 1.00 | 1.00 | 1.00 | -1.00 | 1.00 | 1.00 | 1.00 | -1.00 | -1.00 | -1.00 | -1.00 | 1.00 | -1.00 | 1.00 | -1.00 | -1.00 | -1.00 | 1.00 | -1.00 | 1.00 | 1.00 | 0.00 | 0.00 | 0.00 | 0.00 | 0.00 | 0.00 | 0.00 | 0.00 | 0.00 | 0.00 | 0.00 | 0.00 | 0.00 | 0.00 | sd | sd | sd | sd | sd | sd | sd | sd | sd | sd | sd | sd | sd | sd | sd | sd | sd | sd | sd sd sd sd sd sd sd sd sd sd 1.00 1.00 1.00 1.00 1.00 |
| AREA_SD Permanent crops | 76 | 1.00 | 1.00 | 0.00 | 0.00 | 1.00 | 1.00 | 1.00 | 1.00 | 1.00 | 1.00 | -1.00 | 1.00 | 1.00 | 1.00 | -1.00 | -1.00 | -1.00 | -1.00 | 1.00 | -1.00 | 1.00 | -1.00 | -1.00 | -1.00 | 1.00 | -1.00 | 1.00 | 1.00 | 0.00 | 0.00 | 0.00 | 0.00 | 0.00 | 0.00 | 0.00 | 0.00 | 0.00 | 0.00 | 0.00 | 0.00 | 0.00 | 0.00 | sd | sd | sd | sd | sd | sd | sd | sd | sd | sd | sd | sd | sd | sd | sd | sd | sd | sd | sd sd sd sd sd sd sd sd sd sd 1.00 1.00 1.00 1.00 1.00 1.00 |
| GYRATE_MN Permanent crops | 77 | 1.00 | 1.00 | 0.00 | 0.00 | 1.00 | 1.00 | 1.00 | 1.00 | 1.00 | 1.00 | -1.00 | 1.00 | 1.00 | 1.00 | -1.00 | -1.00 | -1.00 | -1.00 | 1.00 | -1.00 | 1.00 | -1.00 | -1.00 | -1.00 | 1.00 | -1.00 | 1.00 | 1.00 | 0.00 | 0.00 | 0.00 | 0.00 | 0.00 | 0.00 | 0.00 | 0.00 | 0.00 | 0.00 | 0.00 | 0.00 | 0.00 | 0.00 | sd | sd | sd | sd | sd | sd | sd | sd | sd | sd | sd | sd | sd | sd | sd | sd | sd | sd | sd sd sd sd sd sd sd sd sd sd 1.00 1.00 1.00 1.00 1.00 1.00 1.00 |
| GYRATE_SD Permanent crops | 78 | 1.00 | 1.00 | 0.00 | 0.00 | 1.00 | 1.00 | 1.00 | 1.00 | 1.00 | 1.00 | -1.00 | 1.00 | 1.00 | 1.00 | -1.00 | -1.00 | -1.00 | -1.00 | 1.00 | -1.00 | 1.00 | -1.00 | -1.00 | -1.00 | 1.00 | -1.00 | 1.00 | 1.00 | 0.00 | 0.00 | 0.00 | 0.00 | 0.00 | 0.00 | 0.00 | 0.00 | 0.00 | 0.00 | 0.00 | 0.00 | 0.00 | 0.00 | sd | sd | sd | sd | sd | sd | sd | sd | sd | sd | sd | sd | sd | sd | sd | sd | sd | sd | sd sd sd sd sd sd sd sd sd sd 1.00 1.00 1.00 1.00 1.00 1.00 1.00 1.00 |
| PARA_MN Permanent crops | 79 | -1.00 | -1.00 | 0.00 | 0.00 | -1.00 | -1.00 | -1.00 | -1.00 | -1.00 | -1.00 | 1.00 | -1.00 | -1.00 | -1.00 | 1.00 | 1.00 | 1.00 | 1.00 | -1.00 | 1.00 | -1.00 | 1.00 | 1.00 | 1.00 | -1.00 | 1.00 | -1.00 | -1.00 | 0.00 | 0.00 | 0.00 | 0.00 | 0.00 | 0.00 | 0.00 | 0.00 | 0.00 | 0.00 | 0.00 | 0.00 | 0.00 | 0.00 | sd | sd | sd | sd | sd | sd | sd | sd | sd | sd | sd | sd | sd | sd | sd | sd | sd | sd | sd sd sd sd sd sd sd sd sd sd -1.00 -1.00 -1.00 -1.00 -1.00 -1.00 -1.00 -1.00 1.00 |
| PARA_SD Permanent crops. | 80 | 1.00 | 1.00 | 0.00 | 0.00 | 1.00 | 1.00 | 1.00 | 1.00 | 1.00 | 1.00 | -1.00 | 1.00 | 1.00 | 1.00 | -1.00 | -1.00 | -1.00 | -1.00 | 1.00 | -1.00 | 1.00 | -1.00 | -1.00 | -1.00 | 1.00 | -1.00 | 1.00 | 1.00 | 0.00 | 0.00 | 0.00 | 0.00 | 0.00 | 0.00 | 0.00 | 0.00 | 0.00 | 0.00 | 0.00 | 0.00 | 0.00 | 0.00 | sd | sd | sd | sd | sd | sd | sd | sd | sd | sd | sd | sd | sd | sd | sd | sd | sd | sd | sd sd sd sd sd sd sd sd sd sd 1.00 1.00 1.00 1.00 1.00 1.00 1.00 1.00 -1.00 1.00 |
| CONTIG_MN Permanent crops | 81 | 1.00 | 1.00 | 0.00 | 0.00 | 1.00 | 1.00 | 1.00 | 1.00 | 1.00 | 1.00 | -1.00 | 1.00 | 1.00 | 1.00 | -1.00 | -1.00 | -1.00 | -1.00 | 1.00 | -1.00 | 1.00 | -1.00 | -1.00 | -1.00 | 1.00 | -1.00 | 1.00 | 1.00 | 0.00 | 0.00 | 0.00 | 0.00 | 0.00 | 0.00 | 0.00 | 0.00 | 0.00 | 0.00 | 0.00 | 0.00 | 0.00 | 0.00 | sd | sd | sd | sd | sd | sd | sd | sd | sd | sd | sd | sd | sd | sd | sd | sd | sd | sd | sd sd sd sd sd sd sd sd sd sd 1.00 1.00 1.00 1.00 1.00 1.00 1.00 1.00 -1.00 1.00 1.00 |
| CONTIG_SD Permanent crops | 82 | 1.00 | 1.00 | 0.00 | 0.00 | 1.00 | 1.00 | 1.00 | 1.00 | 1.00 | 1.00 | -1.00 | 1.00 | 1.00 | 1.00 | -1.00 | -1.00 | -1.00 | -1.00 | 1.00 | -1.00 | 1.00 | -1.00 | -1.00 | -1.00 | 1.00 | -1.00 | 1.00 | 1.00 | 0.00 | 0.00 | 0.00 | 0.00 | 0.00 | 0.00 | 0.00 | 0.00 | 0.00 | 0.00 | 0.00 | 0.00 | 0.00 | 0.00 | sd | sd | sd | sd | sd | sd | sd | sd | sd | sd | sd | sd | sd | sd | sd | sd | sd | sd | sd sd sd sd sd sd sd sd sd sd 1.00 1.00 1.00 1.00 1.00 1.00 1.00 1.00 -1.00 1.00 1.00 1.00 |
| COHESION Permanent crops | 83 | 1.00 | 1.00 | 0.00 | 0.00 | 1.00 | 1.00 | 1.00 | 1.00 | 1.00 | 1.00 | -1.00 | 1.00 | 1.00 | 1.00 | -1.00 | -1.00 | -1.00 | -1.00 | 1.00 | -1.00 | 1.00 | -1.00 | -1.00 | -1.00 | 1.00 | -1.00 | 1.00 | 1.00 | 0.00 | 0.00 | 0.00 | 0.00 | 0.00 | 0.00 | 0.00 | 0.00 | 0.00 | 0.00 | 0.00 | 0.00 | 0.00 | 0.00 | sd | sd | sd | sd | sd | sd | sd | sd | sd | sd | sd | sd | sd | sd | sd | sd | sd | sd | sd sd sd sd sd sd sd sd sd sd 1.00 1.00 1.00 1.00 1.00 1.00 1.00 1.00 -1.00 1.00 1.00 1.00 1.00 |
| MESH Permanent crops.. | 84 | 1.00 | 1.00 | 0.00 | 0.00 | 1.00 | 1.00 | 1.00 | 1.00 | 1.00 | 1.00 | -1.00 | 1.00 | 1.00 | 1.00 | -1.00 | -1.00 | -1.00 | -1.00 | 1.00 | -1.00 | 1.00 | -1.00 | -1.00 | -1.00 | 1.00 | -1.00 | 1.00 | 1.00 | 0.00 | 0.00 | 0.00 | 0.00 | 0.00 | 0.00 | 0.00 | 0.00 | 0.00 | 0.00 | 0.00 | 0.00 | 0.00 | 0.00 | sd | sd | sd | sd | sd | sd | sd | sd | sd | sd | sd | sd | sd | sd | sd | sd | sd | sd | sd sd sd sd sd sd sd sd sd sd 1.00 1.00 1.00 1.00 1.00 1.00 1.00 1.00 -1.00 1.00 1.00 1.00 1.00 1.00 |

**Appendix S4:**

**Table S3**. Spearman correlation between 16 landscape metrics for each land class at 1000 m area of influence. We found 7 independent metrics at three classes: Pastures (CA, AREA_SD and CONTIG_MN) Bodies of water (AREA_MN and PARA_MN) and Urban buildings (CONTIG_SD and COHESION). The link for downloading the spreadsheet online is: https://docs.google.com/spreadsheets/d/1N3pfuOvuHY8I6MCeoah7X7CsHO6J4bsD6dAkwXfmwFI/edit?usp=sharing

| 1000 m buffer | Metric ID | 1 | 2 | 3 | 4 | 5 | 6 | 7 | 8 | 9 | 10 | 11 | 12 | 13 | 14 | 15 | 16 | 17 | 18 | 19 | 20 | 21 | 22 | 23 | 24 | 25 | 26 | 27 | 28 | 29 | 30 | 31 | 32 | 33 | 34 | 35 | 36 | 37 | 38 | 39 | 40 | 41 | 42 | 43 | 44 | 45 | 46 | 47 | 48 | 49 | 50 | 51 | 52 | 53 | 54 | 55 | 56 | 57 | 58 | 59 | 60 | 6162 63 64 65 66 67 68 69 70 71 72 73 74 75 76 77 78 79 80 81 82 83 84 |
| --- | --- | --- | --- | --- | --- | --- | --- | --- | --- | --- | --- | --- | --- | --- | --- | --- | --- | --- | --- | --- | --- | --- | --- | --- | --- | --- | --- | --- | --- | --- | --- | --- | --- | --- | --- | --- | --- | --- | --- | --- | --- | --- | --- | --- | --- | --- | --- | --- | --- | --- | --- | --- | --- | --- | --- | --- | --- | --- | --- | --- | --- | --- |
| CA Pastures | 1 | 1.00 |  |  |  |  |  |  |  |  |  |  |  |  |  |  |  |  |  |  |  |  |  |  |  |  |  |  |  |  |  |  |  |  |  |  |  |  |  |  |  |  |  |  |  |  |  |  |  |  |  |  |  |  |  |  |  |  |  |  |  |  |
| PLAND Pastures | 2 | 1.00 | 1.00 |  |  |  |  |  |  |  |  |  |  |  |  |  |  |  |  |  |  |  |  |  |  |  |  |  |  |  |  |  |  |  |  |  |  |  |  |  |  |  |  |  |  |  |  |  |  |  |  |  |  |  |  |  |  |  |  |  |  |  |
| NP Pastures | 3 | -0.39 | -0.39 | 1.00 |  |  |  |  |  |  |  |  |  |  |  |  |  |  |  |  |  |  |  |  |  |  |  |  |  |  |  |  |  |  |  |  |  |  |  |  |  |  |  |  |  |  |  |  |  |  |  |  |  |  |  |  |  |  |  |  |  |  |
| PD Pastures | 4 | -0.39 | -0.39 | 0.97 | 1.00 |  |  |  |  |  |  |  |  |  |  |  |  |  |  |  |  |  |  |  |  |  |  |  |  |  |  |  |  |  |  |  |  |  |  |  |  |  |  |  |  |  |  |  |  |  |  |  |  |  |  |  |  |  |  |  |  |  |
| AREA_MN Pastures | 5 | 0.70 | 0.70 | -0.91 | -0.90 | 1.00 |  |  |  |  |  |  |  |  |  |  |  |  |  |  |  |  |  |  |  |  |  |  |  |  |  |  |  |  |  |  |  |  |  |  |  |  |  |  |  |  |  |  |  |  |  |  |  |  |  |  |  |  |  |  |  |  |
| AREA_SD Pastures | 6 | 0.15 | 0.15 | 0.58 | 0.58 | -0.37 | 1.00 |  |  |  |  |  |  |  |  |  |  |  |  |  |  |  |  |  |  |  |  |  |  |  |  |  |  |  |  |  |  |  |  |  |  |  |  |  |  |  |  |  |  |  |  |  |  |  |  |  |  |  |  |  |  |  |
| GYRATE_MN Pastures | 7 | 0.57 | 0.57 | -0.96 | -0.93 | 0.96 | -0.50 | 1.00 |  |  |  |  |  |  |  |  |  |  |  |  |  |  |  |  |  |  |  |  |  |  |  |  |  |  |  |  |  |  |  |  |  |  |  |  |  |  |  |  |  |  |  |  |  |  |  |  |  |  |  |  |  |  |
| GYRATE_SD Pastures | 8 | -0.12 | -0.12 | 0.75 | 0.68 | -0.62 | 0.88 | -0.70 | 1.00 |  |  |  |  |  |  |  |  |  |  |  |  |  |  |  |  |  |  |  |  |  |  |  |  |  |  |  |  |  |  |  |  |  |  |  |  |  |  |  |  |  |  |  |  |  |  |  |  |  |  |  |  |  |
| PARA_MN Pastures | 9 | -0.44 | -0.44 | 0.90 | 0.93 | -0.87 | 0.62 | -0.92 | 0.71 | 1.00 |  |  |  |  |  |  |  |  |  |  |  |  |  |  |  |  |  |  |  |  |  |  |  |  |  |  |  |  |  |  |  |  |  |  |  |  |  |  |  |  |  |  |  |  |  |  |  |  |  |  |  |  |
| PARA_SD Pastures | 10 | -0.32 | -0.32 | 0.92 | 0.93 | -0.82 | 0.64 | -0.91 | 0.73 | 0.98 | 1.00 |  |  |  |  |  |  |  |  |  |  |  |  |  |  |  |  |  |  |  |  |  |  |  |  |  |  |  |  |  |  |  |  |  |  |  |  |  |  |  |  |  |  |  |  |  |  |  |  |  |  |  |
| CONTIG_MN Pastures | 11 | 0.44 | 0.44 | -0.90 | -0.93 | 0.87 | -0.62 | 0.92 | -0.71 | -1.00 | -0.98 | 1.00 |  |  |  |  |  |  |  |  |  |  |  |  |  |  |  |  |  |  |  |  |  |  |  |  |  |  |  |  |  |  |  |  |  |  |  |  |  |  |  |  |  |  |  |  |  |  |  |  |  |  |
| CONTIG_SD Pastures | 12 | -0.32 | -0.32 | 0.92 | 0.93 | -0.82 | 0.64 | -0.91 | 0.73 | 0.98 | 1.00 | -0.98 | 1.00 |  |  |  |  |  |  |  |  |  |  |  |  |  |  |  |  |  |  |  |  |  |  |  |  |  |  |  |  |  |  |  |  |  |  |  |  |  |  |  |  |  |  |  |  |  |  |  |  |  |
| COHESION Pastures | 13 | 0.86 | 0.86 | -0.76 | -0.77 | 0.94 | -0.13 | 0.86 | -0.39 | -0.74 | -0.66 | 0.74 | -0.66 | 1.00 |  |  |  |  |  |  |  |  |  |  |  |  |  |  |  |  |  |  |  |  |  |  |  |  |  |  |  |  |  |  |  |  |  |  |  |  |  |  |  |  |  |  |  |  |  |  |  |  |
| MESH Pastures | 14 | 0.89 | 0.89 | -0.65 | -0.64 | 0.87 | 0.00 | 0.78 | -0.25 | -0.61 | -0.53 | 0.61 | -0.53 | 0.97 | 1.00 |  |  |  |  |  |  |  |  |  |  |  |  |  |  |  |  |  |  |  |  |  |  |  |  |  |  |  |  |  |  |  |  |  |  |  |  |  |  |  |  |  |  |  |  |  |  |  |
| CA Bodies of water | 15 | -0.21 | -0.21 | 0.63 | 0.09 | -1.00 | -0.21 | -1.00 | 0.09 | 0.70 | 0.70 | -0.70 | 0.70 | -0.89 | -0.62 | 1.00 |  |  |  |  |  |  |  |  |  |  |  |  |  |  |  |  |  |  |  |  |  |  |  |  |  |  |  |  |  |  |  |  |  |  |  |  |  |  |  |  |  |  |  |  |  |  |
| PLAND Bodies of water | 16 | -0.21 | -0.21 | 0.63 | 0.09 | -1.00 | -0.21 | -1.00 | 0.09 | 0.70 | 0.70 | -0.70 | 0.70 | -0.89 | -0.62 | 1.00 | 1.00 |  |  |  |  |  |  |  |  |  |  |  |  |  |  |  |  |  |  |  |  |  |  |  |  |  |  |  |  |  |  |  |  |  |  |  |  |  |  |  |  |  |  |  |  |  |
| NP Bodies of water | 17 | -0.49 | -0.49 | 0.26 | 0.16 | -0.81 | -0.49 | -0.81 | -0.49 | 0.81 | 0.81 | -0.81 | 0.81 | -0.81 | -0.81 | 0.81 | 0.81 | 1.00 |  |  |  |  |  |  |  |  |  |  |  |  |  |  |  |  |  |  |  |  |  |  |  |  |  |  |  |  |  |  |  |  |  |  |  |  |  |  |  |  |  |  |  |  |
| PD Bodies of water | 18 | -0.04 | -0.04 | 0.43 | 0.66 | -0.58 | -0.04 | -0.58 | -0.43 | 0.97 | 0.97 | -0.97 | 0.97 | -0.39 | -0.39 | 0.58 | 0.58 | 0.84 | 1.00 |  |  |  |  |  |  |  |  |  |  |  |  |  |  |  |  |  |  |  |  |  |  |  |  |  |  |  |  |  |  |  |  |  |  |  |  |  |  |  |  |  |  |  |
| AREA_MN Bodies of water | 19 | -0.21 | -0.21 | 0.63 | 0.09 | -1.00 | -0.21 | -1.00 | 0.09 | 0.70 | 0.70 | -0.70 | 0.70 | -0.89 | -0.62 | 1.00 | 1.00 | 0.81 | 0.58 | 1.00 |  |  |  |  |  |  |  |  |  |  |  |  |  |  |  |  |  |  |  |  |  |  |  |  |  |  |  |  |  |  |  |  |  |  |  |  |  |  |  |  |  |  |
| AREA_SD Bodies of water | 20 | -0.49 | -0.49 | 0.26 | 0.16 | -0.81 | -0.49 | -0.81 | -0.49 | 0.81 | 0.81 | -0.81 | 0.81 | -0.81 | -0.81 | 0.81 | 0.81 | 1.00 | 0.84 | 0.81 | 1.00 |  |  |  |  |  |  |  |  |  |  |  |  |  |  |  |  |  |  |  |  |  |  |  |  |  |  |  |  |  |  |  |  |  |  |  |  |  |  |  |  |  |
| GYRATE_MN Bodies of water | 21 | -0.21 | -0.21 | 0.63 | 0.09 | -1.00 | -0.21 | -1.00 | 0.09 | 0.70 | 0.70 | -0.70 | 0.70 | -0.89 | -0.62 | 1.00 | 1.00 | 0.81 | 0.58 | 1.00 | 0.81 | 1.00 |  |  |  |  |  |  |  |  |  |  |  |  |  |  |  |  |  |  |  |  |  |  |  |  |  |  |  |  |  |  |  |  |  |  |  |  |  |  |  |  |
| GYRATE_SD Bodies of water | 22 | -0.49 | -0.49 | 0.26 | 0.16 | -0.81 | -0.49 | -0.81 | -0.49 | 0.81 | 0.81 | -0.81 | 0.81 | -0.81 | -0.81 | 0.81 | 0.81 | 1.00 | 0.84 | 0.81 | 1.00 | 0.81 | 1.00 |  |  |  |  |  |  |  |  |  |  |  |  |  |  |  |  |  |  |  |  |  |  |  |  |  |  |  |  |  |  |  |  |  |  |  |  |  |  |  |
| PARA_MN Bodies of water | 23 | -0.70 | -0.70 | -0.63 | -1.00 | 0.09 | -0.70 | 0.09 | -0.09 | -0.70 | -0.70 | 0.70 | -0.70 | -0.32 | -0.43 | -0.09 | -0.09 | -0.16 | -0.66 | -0.09 | -0.16 | -0.09 | -0.16 | 1.00 |  |  |  |  |  |  |  |  |  |  |  |  |  |  |  |  |  |  |  |  |  |  |  |  |  |  |  |  |  |  |  |  |  |  |  |  |  |  |
| PARA_SD Bodies of water | 24 | -0.49 | -0.49 | 0.26 | 0.16 | -0.81 | -0.49 | -0.81 | -0.49 | 0.81 | 0.81 | -0.81 | 0.81 | -0.81 | -0.81 | 0.81 | 0.81 | 1.00 | 0.84 | 0.81 | 1.00 | 0.81 | 1.00 | -0.16 | 1.00 |  |  |  |  |  |  |  |  |  |  |  |  |  |  |  |  |  |  |  |  |  |  |  |  |  |  |  |  |  |  |  |  |  |  |  |  |  |
| CONTIG_MN Bodies of water | 25 | 0.70 | 0.70 | 0.63 | 1.00 | -0.09 | 0.70 | -0.09 | 0.09 | 0.70 | 0.70 | -0.70 | 0.70 | 0.32 | 0.43 | 0.09 | 0.09 | 0.16 | 0.66 | 0.09 | 0.16 | 0.09 | 0.16 | -1.00 | 0.16 | 1.00 |  |  |  |  |  |  |  |  |  |  |  |  |  |  |  |  |  |  |  |  |  |  |  |  |  |  |  |  |  |  |  |  |  |  |  |  |
| CONTIG_SD Bodies of water | 26 | -0.49 | -0.49 | 0.26 | 0.16 | -0.81 | -0.49 | -0.81 | -0.49 | 0.81 | 0.81 | -0.81 | 0.81 | -0.81 | -0.81 | 0.81 | 0.81 | 1.00 | 0.84 | 0.81 | 1.00 | 0.81 | 1.00 | -0.16 | 1.00 | 0.16 | 1.00 |  |  |  |  |  |  |  |  |  |  |  |  |  |  |  |  |  |  |  |  |  |  |  |  |  |  |  |  |  |  |  |  |  |  |  |
| COHESION Bodies of water | 27 | -0.21 | -0.21 | 0.63 | 0.09 | -1.00 | -0.21 | -1.00 | 0.09 | 0.70 | 0.70 | -0.70 | 0.70 | -0.89 | -0.62 | 1.00 | 1.00 | 0.81 | 0.58 | 1.00 | 0.81 | 1.00 | 0.81 | -0.09 | 0.81 | 0.09 | 0.81 | 1.00 |  |  |  |  |  |  |  |  |  |  |  |  |  |  |  |  |  |  |  |  |  |  |  |  |  |  |  |  |  |  |  |  |  |  |
| MESH Bodies of water | 28 | -0.21 | -0.21 | 0.63 | 0.09 | -1.00 | -0.21 | -1.00 | 0.09 | 0.70 | 0.70 | -0.70 | 0.70 | -0.89 | -0.62 | 1.00 | 1.00 | 0.81 | 0.58 | 1.00 | 0.81 | 1.00 | 0.81 | -0.09 | 0.81 | 0.09 | 0.81 | 1.00 | 1.00 |  |  |  |  |  |  |  |  |  |  |  |  |  |  |  |  |  |  |  |  |  |  |  |  |  |  |  |  |  |  |  |  |  |
| CA Native forest | 29 | -0.20 | -0.20 | 0.42 | 0.39 | -0.37 | 0.23 | -0.29 | 0.29 | 0.25 | 0.23 | -0.25 | 0.23 | -0.37 | -0.37 | -0.89 | -0.89 | -0.81 | -0.39 | -0.89 | -0.81 | -0.89 | -0.81 | -0.32 | -0.81 | 0.32 | -0.81 | -0.89 | -0.89 | 1.00 |  |  |  |  |  |  |  |  |  |  |  |  |  |  |  |  |  |  |  |  |  |  |  |  |  |  |  |  |  |  |  |  |
| PLAND Native forest | 30 | -0.20 | -0.20 | 0.42 | 0.39 | -0.37 | 0.23 | -0.29 | 0.29 | 0.25 | 0.23 | -0.25 | 0.23 | -0.37 | -0.37 | -0.89 | -0.89 | -0.81 | -0.39 | -0.89 | -0.81 | -0.89 | -0.81 | -0.32 | -0.81 | 0.32 | -0.81 | -0.89 | -0.89 | 1.00 | 1.00 |  |  |  |  |  |  |  |  |  |  |  |  |  |  |  |  |  |  |  |  |  |  |  |  |  |  |  |  |  |  |  |
| NP Native forest | 31 | 0.17 | 0.17 | 0.11 | 0.11 | -0.02 | 0.08 | 0.02 | -0.05 | 0.00 | 0.01 | 0.00 | 0.01 | 0.00 | -0.06 | -0.49 | -0.49 | -0.40 | 0.17 | -0.49 | -0.40 | -0.49 | -0.40 | -0.81 | -0.40 | 0.81 | -0.40 | -0.49 | -0.49 | 0.73 | 0.73 | 1.00 |  |  |  |  |  |  |  |  |  |  |  |  |  |  |  |  |  |  |  |  |  |  |  |  |  |  |  |  |  |  |
| PD Native forest | 32 | 0.00 | 0.00 | 0.16 | 0.22 | -0.15 | -0.02 | -0.07 | -0.16 | 0.10 | 0.09 | -0.10 | 0.09 | -0.17 | -0.22 | -0.04 | -0.04 | 0.17 | 0.68 | -0.04 | 0.17 | -0.04 | 0.17 | -0.97 | 0.17 | 0.97 | 0.17 | -0.04 | -0.04 | 0.61 | 0.61 | 0.93 | 1.00 |  |  |  |  |  |  |  |  |  |  |  |  |  |  |  |  |  |  |  |  |  |  |  |  |  |  |  |  |  |
| AREA_MN Native forest | 33 | -0.43 | -0.43 | 0.49 | 0.44 | -0.50 | 0.22 | -0.43 | 0.48 | 0.36 | 0.32 | -0.36 | 0.32 | -0.50 | -0.42 | -0.70 | -0.70 | -0.81 | -0.97 | -0.70 | -0.81 | -0.70 | -0.81 | 0.70 | -0.81 | -0.70 | -0.81 | -0.70 | -0.70 | 0.59 | 0.59 | -0.10 | -0.20 | 1.00 |  |  |  |  |  |  |  |  |  |  |  |  |  |  |  |  |  |  |  |  |  |  |  |  |  |  |  |  |
| AREA_SD Native forest | 34 | 0.17 | 0.17 | 0.36 | 0.42 | -0.21 | 0.30 | -0.18 | 0.18 | 0.31 | 0.32 | -0.31 | 0.32 | -0.12 | -0.08 | -0.49 | -0.49 | -0.40 | 0.17 | -0.49 | -0.40 | -0.49 | -0.40 | -0.81 | -0.40 | 0.81 | -0.40 | -0.49 | -0.49 | 0.80 | 0.80 | 0.83 | 0.78 | 0.21 | 1.00 |  |  |  |  |  |  |  |  |  |  |  |  |  |  |  |  |  |  |  |  |  |  |  |  |  |  |  |
| GYRATE_MN Native forest | 35 | -0.05 | -0.05 | -0.02 | -0.08 | 0.03 | 0.05 | 0.04 | 0.26 | -0.15 | -0.13 | 0.15 | -0.13 | 0.03 | 0.12 | -0.43 | -0.43 | -0.81 | -0.97 | -0.43 | -0.81 | -0.43 | -0.81 | 0.58 | -0.81 | -0.58 | -0.81 | -0.43 | -0.43 | 0.07 | 0.07 | -0.47 | -0.65 | 0.67 | -0.24 | 1.00 |  |  |  |  |  |  |  |  |  |  |  |  |  |  |  |  |  |  |  |  |  |  |  |  |  |  |
| GYRATE_SD Native forest | 36 | 0.36 | 0.36 | 0.13 | 0.20 | 0.03 | 0.22 | 0.06 | 0.00 | 0.07 | 0.08 | -0.07 | 0.08 | 0.11 | 0.11 | -0.49 | -0.49 | -0.40 | 0.17 | -0.49 | -0.40 | -0.49 | -0.40 | -0.81 | -0.40 | 0.81 | -0.40 | -0.49 | -0.49 | 0.67 | 0.67 | 0.91 | 0.86 | -0.04 | 0.92 | -0.43 | 1.00 |  |  |  |  |  |  |  |  |  |  |  |  |  |  |  |  |  |  |  |  |  |  |  |  |  |
| PARA_MN Native forest | 37 | 0.63 | 0.63 | -0.24 | -0.10 | 0.36 | 0.07 | 0.32 | -0.24 | -0.18 | -0.15 | 0.18 | -0.15 | 0.46 | 0.49 | 0.09 | 0.09 | 0.16 | 0.66 | 0.09 | 0.16 | 0.09 | 0.16 | -1.00 | 0.16 | 1.00 | 0.16 | 0.09 | 0.09 | -0.36 | -0.36 | 0.14 | 0.26 | -0.67 | 0.16 | -0.49 | 0.37 | 1.00 |  |  |  |  |  |  |  |  |  |  |  |  |  |  |  |  |  |  |  |  |  |  |  |  |
| PARA_SD Native forest | 38 | 0.36 | 0.36 | 0.13 | 0.20 | 0.03 | 0.18 | 0.08 | -0.01 | 0.09 | 0.10 | -0.09 | 0.10 | 0.12 | 0.15 | -0.49 | -0.49 | -0.40 | 0.17 | -0.49 | -0.40 | -0.49 | -0.40 | -0.81 | -0.40 | 0.81 | -0.40 | -0.49 | -0.49 | 0.64 | 0.64 | 0.86 | 0.81 | -0.01 | 0.92 | -0.39 | 0.98 | 0.37 | 1.00 |  |  |  |  |  |  |  |  |  |  |  |  |  |  |  |  |  |  |  |  |  |  |  |
| CONTIG_MN Native forest | 39 | -0.63 | -0.63 | 0.24 | 0.10 | -0.36 | -0.07 | -0.32 | 0.24 | 0.18 | 0.15 | -0.18 | 0.15 | -0.46 | -0.49 | -0.09 | -0.09 | -0.16 | -0.66 | -0.09 | -0.16 | -0.09 | -0.16 | 1.00 | -0.16 | -1.00 | -0.16 | -0.09 | -0.09 | 0.36 | 0.36 | -0.14 | -0.26 | 0.67 | -0.16 | 0.49 | -0.37 | -1.00 | -0.37 | 1.00 |  |  |  |  |  |  |  |  |  |  |  |  |  |  |  |  |  |  |  |  |  |  |
| CONTIG_SD Native forest | 40 | 0.36 | 0.36 | 0.13 | 0.20 | 0.03 | 0.18 | 0.08 | -0.01 | 0.09 | 0.10 | -0.09 | 0.10 | 0.12 | 0.15 | -0.49 | -0.49 | -0.40 | 0.17 | -0.49 | -0.40 | -0.49 | -0.40 | -0.81 | -0.40 | 0.81 | -0.40 | -0.49 | -0.49 | 0.64 | 0.64 | 0.86 | 0.81 | -0.01 | 0.92 | -0.39 | 0.98 | 0.37 | 1.00 | -0.37 | 1.00 |  |  |  |  |  |  |  |  |  |  |  |  |  |  |  |  |  |  |  |  |  |
| COHESION Native forest | 41 | -0.21 | -0.21 | 0.50 | 0.52 | -0.43 | 0.31 | -0.35 | 0.40 | 0.35 | 0.31 | -0.35 | 0.31 | -0.41 | -0.34 | -0.89 | -0.89 | -0.81 | -0.39 | -0.89 | -0.81 | -0.89 | -0.81 | -0.32 | -0.81 | 0.32 | -0.81 | -0.89 | -0.89 | 0.81 | 0.81 | 0.31 | 0.25 | 0.85 | 0.60 | 0.33 | 0.45 | -0.31 | 0.46 | 0.31 | 0.46 | 1.00 |  |  |  |  |  |  |  |  |  |  |  |  |  |  |  |  |  |  |  |  |
| MESH Native forest | 42 | -0.18 | -0.18 | 0.48 | 0.48 | -0.41 | 0.27 | -0.33 | 0.34 | 0.33 | 0.31 | -0.33 | 0.31 | -0.39 | -0.36 | -0.89 | -0.89 | -0.81 | -0.39 | -0.89 | -0.81 | -0.89 | -0.81 | -0.32 | -0.81 | 0.32 | -0.81 | -0.89 | -0.89 | 0.96 | 0.96 | 0.61 | 0.51 | 0.71 | 0.80 | 0.15 | 0.65 | -0.32 | 0.64 | 0.32 | 0.64 | 0.92 | 1.00 |  |  |  |  |  |  |  |  |  |  |  |  |  |  |  |  |  |  |  |
| CA Transitory crops | 43 | -0.72 | -0.72 | -0.20 | -0.11 | -0.23 | -0.78 | 0.10 | -0.78 | -0.20 | -0.31 | 0.20 | -0.31 | -0.31 | -0.38 | 1.00 | 1.00 | 1.00 | 1.00 | 1.00 | 1.00 | 1.00 | 1.00 | -1.00 | 1.00 | 1.00 | 1.00 | 1.00 | 1.00 | -0.07 | -0.07 | -0.35 | 0.27 | -0.07 | -0.35 | -0.19 | -0.35 | 0.43 | -0.35 | -0.43 | -0.35 | -0.07 | -0.07 | 1.00 |  |  |  |  |  |  |  |  |  |  |  |  |  |  |  |  |  |  |
| PLAND Transitory crops | 44 | -0.72 | -0.72 | -0.20 | -0.11 | -0.23 | -0.78 | 0.10 | -0.78 | -0.20 | -0.31 | 0.20 | -0.31 | -0.31 | -0.38 | 1.00 | 1.00 | 1.00 | 1.00 | 1.00 | 1.00 | 1.00 | 1.00 | -1.00 | 1.00 | 1.00 | 1.00 | 1.00 | 1.00 | -0.07 | -0.07 | -0.35 | 0.27 | -0.07 | -0.35 | -0.19 | -0.35 | 0.43 | -0.35 | -0.43 | -0.35 | -0.07 | -0.07 | 1.00 | 1.00 |  |  |  |  |  |  |  |  |  |  |  |  |  |  |  |  |  |
| NP Transitory crops | 45 | -0.11 | -0.11 | -0.27 | -0.22 | 0.02 | -0.42 | 0.20 | -0.42 | -0.20 | -0.27 | 0.20 | -0.27 | -0.08 | -0.08 | 1.00 | 1.00 | 1.00 | 1.00 | 1.00 | 1.00 | 1.00 | 1.00 | -1.00 | 1.00 | 1.00 | 1.00 | 1.00 | 1.00 | -0.56 | -0.56 | -0.47 | -0.05 | -0.56 | -0.47 | -0.45 | -0.47 | 0.67 | -0.47 | -0.67 | -0.47 | -0.56 | -0.56 | 0.62 | 0.62 | 1.00 |  |  |  |  |  |  |  |  |  |  |  |  |  |  |  |  |
| PD Transitory crops | 46 | -0.27 | -0.27 | -0.15 | -0.10 | -0.12 | -0.48 | 0.07 | -0.48 | -0.13 | -0.21 | 0.13 | -0.21 | -0.22 | -0.22 | 1.00 | 1.00 | 1.00 | 1.00 | 1.00 | 1.00 | 1.00 | 1.00 | -1.00 | 1.00 | 1.00 | 1.00 | 1.00 | 1.00 | -0.53 | -0.53 | -0.55 | -0.04 | -0.53 | -0.55 | -0.54 | -0.55 | 0.64 | -0.55 | -0.64 | -0.55 | -0.53 | -0.53 | 0.75 | 0.75 | 0.96 | 1.00 |  |  |  |  |  |  |  |  |  |  |  |  |  |  |  |
| AREA_MN Transitory crops | 47 | -0.47 | -0.47 | 0.22 | 0.22 | -0.26 | -0.17 | -0.19 | -0.17 | 0.14 | 0.17 | -0.14 | 0.17 | -0.23 | -0.33 | -1.00 | -1.00 | -1.00 | -1.00 | -1.00 | -1.00 | -1.00 | -1.00 | 1.00 | -1.00 | -1.00 | -1.00 | -1.00 | -1.00 | 0.75 | 0.75 | 0.53 | 0.52 | 0.75 | 0.53 | 0.53 | 0.53 | -0.52 | 0.53 | 0.52 | 0.53 | 0.75 | 0.75 | 0.03 | 0.03 | -0.73 | -0.61 | 1.00 |  |  |  |  |  |  |  |  |  |  |  |  |  |  |
| AREA_SD Transitory crops | 48 | -0.30 | -0.30 | -0.58 | -0.52 | 0.24 | -0.66 | 0.48 | -0.66 | -0.48 | -0.58 | 0.48 | -0.58 | 0.09 | 0.09 | -1.00 | -1.00 | -1.00 | -1.00 | -1.00 | -1.00 | -1.00 | -1.00 | 1.00 | -1.00 | -1.00 | -1.00 | -1.00 | -1.00 | -0.14 | -0.14 | -0.47 | -0.22 | -0.14 | -0.47 | 0.01 | -0.47 | 0.24 | -0.47 | -0.24 | -0.47 | -0.14 | -0.14 | 0.66 | 0.66 | 0.83 | 0.79 | -0.53 | 1.00 |  |  |  |  |  |  |  |  |  |  |  |  |  |
| GYRATE_MN Transitory crops | 49 | -0.56 | -0.56 | 0.35 | 0.37 | -0.42 | -0.05 | -0.34 | -0.05 | 0.31 | 0.33 | -0.31 | 0.33 | -0.39 | -0.45 | -1.00 | -1.00 | -1.00 | -1.00 | -1.00 | -1.00 | -1.00 | -1.00 | 1.00 | -1.00 | -1.00 | -1.00 | -1.00 | -1.00 | 0.75 | 0.75 | 0.53 | 0.52 | 0.75 | 0.53 | 0.53 | 0.53 | -0.52 | 0.53 | 0.52 | 0.53 | 0.75 | 0.75 | 0.00 | 0.00 | -0.73 | -0.61 | 0.97 | -0.53 | 1.00 |  |  |  |  |  |  |  |  |  |  |  |  |
| GYRATE_SD Transitory crops | 50 | -0.10 | -0.10 | -0.37 | -0.36 | 0.16 | -0.50 | 0.32 | -0.50 | -0.32 | -0.37 | 0.32 | -0.37 | 0.01 | 0.01 | -1.00 | -1.00 | -1.00 | -1.00 | -1.00 | -1.00 | -1.00 | -1.00 | 1.00 | -1.00 | -1.00 | -1.00 | -1.00 | -1.00 | -0.35 | -0.35 | -0.47 | -0.22 | -0.35 | -0.47 | -0.26 | -0.47 | 0.35 | -0.47 | -0.35 | -0.47 | -0.35 | -0.35 | 0.54 | 0.54 | 0.94 | 0.90 | -0.73 | 0.91 | -0.73 | 1.00 |  |  |  |  |  |  |  |  |  |  |  |
| PARA_MN Transitory crops | 51 | -0.36 | -0.36 | 0.03 | 0.07 | -0.30 | -0.36 | -0.11 | -0.36 | 0.08 | 0.00 | -0.08 | 0.00 | -0.42 | -0.39 | 1.00 | 1.00 | 1.00 | 1.00 | 1.00 | 1.00 | 1.00 | 1.00 | -1.00 | 1.00 | 1.00 | 1.00 | 1.00 | 1.00 | -0.33 | -0.33 | -0.35 | 0.11 | -0.33 | -0.35 | -0.38 | -0.35 | 0.47 | -0.35 | -0.47 | -0.35 | -0.33 | -0.33 | 0.66 | 0.66 | 0.91 | 0.95 | -0.60 | 0.77 | -0.54 | 0.89 | 1.00 |  |  |  |  |  |  |  |  |  |  |
| PARA_SD Transitory crops | 52 | -0.07 | -0.07 | -0.27 | -0.24 | 0.05 | -0.41 | 0.21 | -0.41 | -0.21 | -0.27 | 0.21 | -0.27 | -0.06 | -0.06 | 1.00 | 1.00 | 1.00 | 1.00 | 1.00 | 1.00 | 1.00 | 1.00 | -1.00 | 1.00 | 1.00 | 1.00 | 1.00 | 1.00 | -0.52 | -0.52 | -0.47 | -0.11 | -0.52 | -0.47 | -0.43 | -0.47 | 0.57 | -0.47 | -0.57 | -0.47 | -0.52 | -0.52 | 0.57 | 0.57 | 0.99 | 0.95 | -0.76 | 0.84 | -0.76 | 0.97 | 0.92 | 1.00 |  |  |  |  |  |  |  |  |  |
| CONTIG_MN Transitory crops | 53 | 0.33 | 0.33 | -0.01 | -0.06 | 0.28 | 0.33 | 0.10 | 0.33 | -0.10 | -0.01 | 0.10 | -0.01 | 0.41 | 0.38 | -1.00 | -1.00 | -1.00 | -1.00 | -1.00 | -1.00 | -1.00 | -1.00 | 1.00 | -1.00 | -1.00 | -1.00 | -1.00 | -1.00 | 0.30 | 0.30 | 0.27 | -0.15 | 0.30 | 0.27 | 0.30 | 0.27 | -0.45 | 0.27 | 0.45 | 0.27 | 0.30 | 0.30 | -0.61 | -0.61 | -0.92 | -0.93 | 0.63 | -0.77 | 0.55 | -0.90 | -0.99 | -0.93 | 1.00 |  |  |  |  |  |  |  |  |
| CONTIG_SD Transitory crops | 54 | -0.07 | -0.07 | -0.27 | -0.24 | 0.05 | -0.41 | 0.21 | -0.41 | -0.21 | -0.27 | 0.21 | -0.27 | -0.06 | -0.06 | 1.00 | 1.00 | 1.00 | 1.00 | 1.00 | 1.00 | 1.00 | 1.00 | -1.00 | 1.00 | 1.00 | 1.00 | 1.00 | 1.00 | -0.52 | -0.52 | -0.47 | -0.11 | -0.52 | -0.47 | -0.43 | -0.47 | 0.57 | -0.47 | -0.57 | -0.47 | -0.52 | -0.52 | 0.57 | 0.57 | 0.99 | 0.95 | -0.76 | 0.84 | -0.76 | 0.97 | 0.92 | 1.00 | -0.93 | 1.00 |  |  |  |  |  |  |  |
| COHESION Transitory crops | 55 | -0.56 | -0.56 | 0.17 | 0.14 | -0.23 | -0.32 | -0.12 | -0.32 | 0.07 | 0.12 | -0.07 | 0.12 | -0.26 | -0.38 | -1.00 | -1.00 | -1.00 | -1.00 | -1.00 | -1.00 | -1.00 | -1.00 | 1.00 | -1.00 | -1.00 | -1.00 | -1.00 | -1.00 | 0.86 | 0.86 | 0.53 | 0.52 | 0.86 | 0.53 | 0.61 | 0.53 | -0.63 | 0.53 | 0.63 | 0.53 | 0.86 | 0.86 | 0.11 | 0.11 | -0.64 | -0.51 | 0.97 | -0.38 | 0.94 | -0.58 | -0.48 | -0.65 | 0.51 | -0.65 | 1.00 |  |  |  |  |  |  |
| MESH Transitory crops | 56 | -0.78 | -0.78 | -0.14 | -0.08 | -0.23 | -0.81 | 0.06 | -0.81 | -0.20 | -0.28 | 0.20 | -0.28 | -0.35 | -0.42 | -1.00 | -1.00 | -1.00 | -1.00 | -1.00 | -1.00 | -1.00 | -1.00 | 1.00 | -1.00 | -1.00 | -1.00 | -1.00 | -1.00 | 0.13 | 0.13 | -0.35 | 0.16 | 0.13 | -0.35 | -0.12 | -0.35 | 0.13 | -0.35 | -0.13 | -0.35 | 0.13 | 0.13 | 0.94 | 0.94 | 0.48 | 0.66 | 0.14 | 0.61 | 0.11 | 0.49 | 0.60 | 0.46 | -0.54 | 0.46 | 0.26 | 1.00 |  |  |  |  |  |
| CA Urban infrastructure | 57 | -0.25 | -0.25 | 0.03 | 0.03 | -0.27 | -0.30 | -0.09 | -0.14 | 0.34 | 0.21 | -0.34 | 0.21 | -0.27 | -0.27 | 0.00 | 0.00 | 0.00 | 0.00 | 0.00 | 0.00 | 0.00 | 0.00 | 0.00 | 0.00 | 0.00 | 0.00 | 0.00 | 0.00 | 0.50 | 0.50 | 0.67 | 0.67 | -0.40 | 0.67 | -0.70 | 0.56 | 0.00 | 0.67 | 0.00 | 0.67 | -0.10 | 0.50 | 1.00 | 1.00 | 1.00 | 1.00 | 1.00 | 1.00 | 1.00 | 1.00 | 1.00 | 1.00 | 1.00 | 1.00 | 1.00 | 1.00 | 1.00 |  |  |  |  |
| PLAND Urban infrastructure | 58 | -0.25 | -0.25 | 0.03 | 0.03 | -0.27 | -0.30 | -0.09 | -0.14 | 0.34 | 0.21 | -0.34 | 0.21 | -0.27 | -0.27 | 0.00 | 0.00 | 0.00 | 0.00 | 0.00 | 0.00 | 0.00 | 0.00 | 0.00 | 0.00 | 0.00 | 0.00 | 0.00 | 0.00 | 0.50 | 0.50 | 0.67 | 0.67 | -0.40 | 0.67 | -0.70 | 0.56 | 0.00 | 0.67 | 0.00 | 0.67 | -0.10 | 0.50 | 1.00 | 1.00 | 1.00 | 1.00 | 1.00 | 1.00 | 1.00 | 1.00 | 1.00 | 1.00 | 1.00 | 1.00 | 1.00 | 1.00 | 1.00 | 1.00 |  |  |  |
| NP Urban infrastructure | 59 | 0.38 | 0.38 | -0.48 | -0.48 | 0.38 | -0.47 | 0.68 | -0.47 | -0.23 | -0.47 | 0.23 | -0.47 | 0.38 | 0.38 | 0.00 | 0.00 | 0.00 | 0.00 | 0.00 | 0.00 | 0.00 | 0.00 | 0.00 | 0.00 | 0.00 | 0.00 | 0.00 | 0.00 | -0.35 | -0.35 | 0.00 | 0.00 | -0.71 | 0.00 | -0.71 | 0.36 | 0.71 | 0.73 | -0.71 | 0.73 | -0.35 | -0.35 | 1.00 | 1.00 | 1.00 | 1.00 | 1.00 | 1.00 | 1.00 | 1.00 | 1.00 | 1.00 | 1.00 | 1.00 | 1.00 | 1.00 | 0.38 | 0.38 | 1.00 |  |  |
| PD Urban infrastructure | 60 | 0.38 | 0.38 | -0.48 | -0.48 | 0.38 | -0.47 | 0.68 | -0.47 | -0.23 | -0.47 | 0.23 | -0.47 | 0.38 | 0.38 | 0.00 | 0.00 | 0.00 | 0.00 | 0.00 | 0.00 | 0.00 | 0.00 | 0.00 | 0.00 | 0.00 | 0.00 | 0.00 | 0.00 | -0.35 | -0.35 | 0.00 | 0.00 | -0.71 | 0.00 | -0.71 | 0.36 | 0.71 | 0.73 | -0.71 | 0.73 | -0.35 | -0.35 | 1.00 | 1.00 | 1.00 | 1.00 | 1.00 | 1.00 | 1.00 | 1.00 | 1.00 | 1.00 | 1.00 | 1.00 | 1.00 | 1.00 | 0.38 | 0.38 | 1.00 | 1.00 |  |
| AREA_MN Urban infrastructure | 61 | -0.31 | -0.31 | 0.17 | 0.17 | -0.34 | -0.08 | -0.41 | 0.00 | 0.27 | 0.34 | -0.27 | 0.34 | -0.34 | -0.34 | 0.00 | 0.00 | 0.00 | 0.00 | 0.00 | 0.00 | 0.00 | 0.00 | 0.00 | 0.00 | 0.00 | 0.00 | 0.00 | 0.00 | 0.60 | 0.60 | 0.72 | 0.72 | -0.10 | 0.72 | -0.30 | 0.41 | -0.30 | 0.21 | 0.30 | 0.21 | -0.10 | 0.60 | 1.00 | 1.00 | 1.00 | 1.00 | 1.00 | 1.00 | 1.00 | 1.00 | 1.00 | 1.00 | 1.00 | 1.00 | 1.00 | 1.00 | 0.67 | 0.67 | -0.38 | -0.38 | 1.00 |
| AREA_SD Urban infrastructure | 62 | 0.38 | 0.38 | -0.48 | -0.48 | 0.38 | -0.47 | 0.68 | -0.47 | -0.23 | -0.47 | 0.23 | -0.47 | 0.38 | 0.38 | 0.00 | 0.00 | 0.00 | 0.00 | 0.00 | 0.00 | 0.00 | 0.00 | 0.00 | 0.00 | 0.00 | 0.00 | 0.00 | 0.00 | -0.35 | -0.35 | 0.00 | 0.00 | -0.71 | 0.00 | -0.71 | 0.36 | 0.71 | 0.73 | -0.71 | 0.73 | -0.35 | -0.35 | 1.00 | 1.00 | 1.00 | 1.00 | 1.00 | 1.00 | 1.00 | 1.00 | 1.00 | 1.00 | 1.00 | 1.00 | 1.00 | 1.00 | 0.38 | 0.38 | 1.00 | 1.00 | -0.381.00 |
| GYRATE_MN Urban infrastructure | 63 | -0.21 | -0.21 | 0.17 | 0.17 | -0.27 | -0.08 | -0.39 | 0.00 | 0.25 | 0.34 | -0.25 | 0.34 | -0.27 | -0.27 | 0.00 | 0.00 | 0.00 | 0.00 | 0.00 | 0.00 | 0.00 | 0.00 | 0.00 | 0.00 | 0.00 | 0.00 | 0.00 | 0.00 | 0.60 | 0.60 | 0.72 | 0.72 | -0.10 | 0.72 | -0.30 | 0.41 | -0.30 | 0.21 | 0.30 | 0.21 | -0.10 | 0.60 | 1.00 | 1.00 | 1.00 | 1.00 | 1.00 | 1.00 | 1.00 | 1.00 | 1.00 | 1.00 | 1.00 | 1.00 | 1.00 | 1.00 | 0.65 | 0.65 | -0.38 | -0.38 | 0.97-0.38 1.00 |
| GYRATE_SD Urban infrastructure | 64 | 0.38 | 0.38 | -0.48 | -0.48 | 0.38 | -0.47 | 0.68 | -0.47 | -0.23 | -0.47 | 0.23 | -0.47 | 0.38 | 0.38 | 0.00 | 0.00 | 0.00 | 0.00 | 0.00 | 0.00 | 0.00 | 0.00 | 0.00 | 0.00 | 0.00 | 0.00 | 0.00 | 0.00 | -0.35 | -0.35 | 0.00 | 0.00 | -0.71 | 0.00 | -0.71 | 0.36 | 0.71 | 0.73 | -0.71 | 0.73 | -0.35 | -0.35 | 1.00 | 1.00 | 1.00 | 1.00 | 1.00 | 1.00 | 1.00 | 1.00 | 1.00 | 1.00 | 1.00 | 1.00 | 1.00 | 1.00 | 0.38 | 0.38 | 1.00 | 1.00 | -0.381.00 -0.38 1.00 |
| PARA_MN Urban infrastructure | 65 | 0.06 | 0.06 | 0.07 | 0.07 | 0.09 | 0.07 | 0.16 | 0.19 | 0.01 | -0.04 | -0.01 | -0.04 | 0.09 | 0.09 | 0.00 | 0.00 | 0.00 | 0.00 | 0.00 | 0.00 | 0.00 | 0.00 | 0.00 | 0.00 | 0.00 | 0.00 | 0.00 | 0.00 | -0.50 | -0.50 | -0.82 | -0.82 | 0.30 | -0.82 | 0.40 | -0.62 | 0.00 | -0.31 | 0.00 | -0.31 | 0.20 | -0.50 | 1.00 | 1.00 | 1.00 | 1.00 | 1.00 | 1.00 | 1.00 | 1.00 | 1.00 | 1.00 | 1.00 | 1.00 | 1.00 | 1.00 | -0.42 | -0.42 | 0.38 | 0.38 | -0.860.38 -0.80 0.38 1.00 |
| PARA_SD Urban infrastructure | 66 | 0.38 | 0.38 | -0.48 | -0.48 | 0.38 | -0.47 | 0.68 | -0.47 | -0.23 | -0.47 | 0.23 | -0.47 | 0.38 | 0.38 | 0.00 | 0.00 | 0.00 | 0.00 | 0.00 | 0.00 | 0.00 | 0.00 | 0.00 | 0.00 | 0.00 | 0.00 | 0.00 | 0.00 | -0.35 | -0.35 | 0.00 | 0.00 | -0.71 | 0.00 | -0.71 | 0.36 | 0.71 | 0.73 | -0.71 | 0.73 | -0.35 | -0.35 | 1.00 | 1.00 | 1.00 | 1.00 | 1.00 | 1.00 | 1.00 | 1.00 | 1.00 | 1.00 | 1.00 | 1.00 | 1.00 | 1.00 | 0.38 | 0.38 | 1.00 | 1.00 | -0.381.00 -0.38 1.00 0.38 1.00 |
| CONTIG_MN Urban infrastructure | 67 | -0.06 | -0.06 | -0.07 | -0.07 | -0.09 | -0.07 | -0.16 | -0.19 | -0.01 | 0.04 | 0.01 | 0.04 | -0.09 | -0.09 | 0.00 | 0.00 | 0.00 | 0.00 | 0.00 | 0.00 | 0.00 | 0.00 | 0.00 | 0.00 | 0.00 | 0.00 | 0.00 | 0.00 | 0.50 | 0.50 | 0.82 | 0.82 | -0.30 | 0.82 | -0.40 | 0.62 | 0.00 | 0.31 | 0.00 | 0.31 | -0.20 | 0.50 | 1.00 | 1.00 | 1.00 | 1.00 | 1.00 | 1.00 | 1.00 | 1.00 | 1.00 | 1.00 | 1.00 | 1.00 | 1.00 | 1.00 | 0.42 | 0.42 | -0.38 | -0.38 | 0.86-0.38 0.80 -0.38 -1.00 -0.38 1.00 |
| CONTIG_SD Urban infrastructure | 68 | 0.38 | 0.38 | -0.48 | -0.48 | 0.38 | -0.47 | 0.68 | -0.47 | -0.23 | -0.47 | 0.23 | -0.47 | 0.38 | 0.38 | 0.00 | 0.00 | 0.00 | 0.00 | 0.00 | 0.00 | 0.00 | 0.00 | 0.00 | 0.00 | 0.00 | 0.00 | 0.00 | 0.00 | -0.35 | -0.35 | 0.00 | 0.00 | -0.71 | 0.00 | -0.71 | 0.36 | 0.71 | 0.73 | -0.71 | 0.73 | -0.35 | -0.35 | 1.00 | 1.00 | 1.00 | 1.00 | 1.00 | 1.00 | 1.00 | 1.00 | 1.00 | 1.00 | 1.00 | 1.00 | 1.00 | 1.00 | 0.38 | 0.38 | 1.00 | 1.00 | -0.381.00 -0.38 1.00 0.38 1.00 -0.38 1.00 |
| COHESION Urban infrastructure | 69 | -0.31 | -0.31 | 0.17 | 0.17 | -0.34 | -0.08 | -0.41 | 0.00 | 0.27 | 0.34 | -0.27 | 0.34 | -0.34 | -0.34 | 0.00 | 0.00 | 0.00 | 0.00 | 0.00 | 0.00 | 0.00 | 0.00 | 0.00 | 0.00 | 0.00 | 0.00 | 0.00 | 0.00 | 0.60 | 0.60 | 0.72 | 0.72 | -0.10 | 0.72 | -0.30 | 0.41 | -0.30 | 0.21 | 0.30 | 0.21 | -0.10 | 0.60 | 1.00 | 1.00 | 1.00 | 1.00 | 1.00 | 1.00 | 1.00 | 1.00 | 1.00 | 1.00 | 1.00 | 1.00 | 1.00 | 1.00 | 0.67 | 0.67 | -0.38 | -0.38 | 1.00-0.38 0.97 -0.38 -0.86 -0.38 0.86 -0.38 1.00 |
| MESH Urban infrastructure | 70 | -0.39 | -0.39 | 0.17 | 0.17 | -0.41 | -0.08 | -0.34 | 0.00 | 0.39 | 0.34 | -0.39 | 0.34 | -0.41 | -0.41 | 0.00 | 0.00 | 0.00 | 0.00 | 0.00 | 0.00 | 0.00 | 0.00 | 0.00 | 0.00 | 0.00 | 0.00 | 0.00 | 0.00 | 0.70 | 0.70 | 0.87 | 0.87 | -0.30 | 0.87 | -0.60 | 0.67 | -0.10 | 0.56 | 0.10 | 0.56 | 0.00 | 0.70 | 1.00 | 1.00 | 1.00 | 1.00 | 1.00 | 1.00 | 1.00 | 1.00 | 1.00 | 1.00 | 1.00 | 1.00 | 1.00 | 1.00 | 0.86 | 0.86 | -0.08 | -0.08 | 0.93-0.08 0.86 -0.08 -0.79 -0.08 0.79 -0.08 0.93 1.00 |
| CA Permanent crops | 71 | 1.00 | 1.00 | 0.00 | 0.00 | 1.00 | 1.00 | 1.00 | 1.00 | -1.00 | -1.00 | 1.00 | -1.00 | 1.00 | 1.00 | 0.00 | 0.00 | 0.00 | 0.00 | 0.00 | 0.00 | 0.00 | 0.00 | 0.00 | 0.00 | 0.00 | 0.00 | 0.00 | 0.00 | -1.00 | -1.00 | -1.00 | -1.00 | 1.00 | -1.00 | 1.00 | -1.00 | 1.00 | -1.00 | -1.00 | -1.00 | 1.00 | -1.00 | 1.00 | 1.00 | 1.00 | 1.00 | 1.00 | 1.00 | 1.00 | 1.00 | 1.00 | 1.00 | 1.00 | 1.00 | 1.00 | 1.00 | -1.00 | -1.00 | 0.00 | 0.00 | -1.000.00 -1.00 0.00 1.00 0.00 -1.00 0.00 -1.00 -1.00 1.00 |
| PLAND Permanent crops | 72 | 1.00 | 1.00 | 0.00 | 0.00 | 1.00 | 1.00 | 1.00 | 1.00 | -1.00 | -1.00 | 1.00 | -1.00 | 1.00 | 1.00 | 0.00 | 0.00 | 0.00 | 0.00 | 0.00 | 0.00 | 0.00 | 0.00 | 0.00 | 0.00 | 0.00 | 0.00 | 0.00 | 0.00 | -1.00 | -1.00 | -1.00 | -1.00 | 1.00 | -1.00 | 1.00 | -1.00 | 1.00 | -1.00 | -1.00 | -1.00 | 1.00 | -1.00 | 1.00 | 1.00 | 1.00 | 1.00 | 1.00 | 1.00 | 1.00 | 1.00 | 1.00 | 1.00 | 1.00 | 1.00 | 1.00 | 1.00 | -1.00 | -1.00 | 0.00 | 0.00 | -1.000.00 -1.00 0.00 1.00 0.00 -1.00 0.00 -1.00 -1.00 1.00 1.00 |
| NP Permanent crops | 73 | 1.00 | 1.00 | 0.00 | 0.00 | 1.00 | 1.00 | 1.00 | 1.00 | -1.00 | -1.00 | 1.00 | -1.00 | 1.00 | 1.00 | 0.00 | 0.00 | 0.00 | 0.00 | 0.00 | 0.00 | 0.00 | 0.00 | 0.00 | 0.00 | 0.00 | 0.00 | 0.00 | 0.00 | -1.00 | -1.00 | -1.00 | -1.00 | 1.00 | -1.00 | 1.00 | -1.00 | 1.00 | -1.00 | -1.00 | -1.00 | 1.00 | -1.00 | 1.00 | 1.00 | 1.00 | 1.00 | 1.00 | 1.00 | 1.00 | 1.00 | 1.00 | 1.00 | 1.00 | 1.00 | 1.00 | 1.00 | -1.00 | -1.00 | 0.00 | 0.00 | -1.000.00 -1.00 0.00 1.00 0.00 -1.00 0.00 -1.00 -1.00 1.00 1.00 1.00 |
| PD Permanent crops | 74 | 1.00 | 1.00 | 0.00 | 0.00 | 1.00 | 1.00 | 1.00 | 1.00 | -1.00 | -1.00 | 1.00 | -1.00 | 1.00 | 1.00 | 0.00 | 0.00 | 0.00 | 0.00 | 0.00 | 0.00 | 0.00 | 0.00 | 0.00 | 0.00 | 0.00 | 0.00 | 0.00 | 0.00 | -1.00 | -1.00 | -1.00 | -1.00 | 1.00 | -1.00 | 1.00 | -1.00 | 1.00 | -1.00 | -1.00 | -1.00 | 1.00 | -1.00 | 1.00 | 1.00 | 1.00 | 1.00 | 1.00 | 1.00 | 1.00 | 1.00 | 1.00 | 1.00 | 1.00 | 1.00 | 1.00 | 1.00 | -1.00 | -1.00 | 0.00 | 0.00 | -1.000.00 -1.00 0.00 1.00 0.00 -1.00 0.00 -1.00 -1.00 1.00 1.00 1.00 1.00 |
| AREA_MN Permanent crops | 75 | -1.00 | -1.00 | 0.00 | 0.00 | -1.00 | -1.00 | -1.00 | -1.00 | 1.00 | 1.00 | -1.00 | 1.00 | -1.00 | -1.00 | 0.00 | 0.00 | 0.00 | 0.00 | 0.00 | 0.00 | 0.00 | 0.00 | 0.00 | 0.00 | 0.00 | 0.00 | 0.00 | 0.00 | 1.00 | 1.00 | 1.00 | 1.00 | -1.00 | 1.00 | -1.00 | 1.00 | -1.00 | 1.00 | 1.00 | 1.00 | -1.00 | 1.00 | 1.00 | 1.00 | 1.00 | 1.00 | 1.00 | 1.00 | 1.00 | 1.00 | 1.00 | 1.00 | 1.00 | 1.00 | 1.00 | 1.00 | 1.00 | 1.00 | 0.00 | 0.00 | 1.000.00 1.00 0.00 -1.00 0.00 1.00 0.00 1.00 1.00 -1.00 -1.00 -1.00 -1.00 1.00 |
| AREA_SD Permanent crops | 76 | 1.00 | 1.00 | 0.00 | 0.00 | 1.00 | 1.00 | 1.00 | 1.00 | -1.00 | -1.00 | 1.00 | -1.00 | 1.00 | 1.00 | 0.00 | 0.00 | 0.00 | 0.00 | 0.00 | 0.00 | 0.00 | 0.00 | 0.00 | 0.00 | 0.00 | 0.00 | 0.00 | 0.00 | -1.00 | -1.00 | -1.00 | -1.00 | 1.00 | -1.00 | 1.00 | -1.00 | 1.00 | -1.00 | -1.00 | -1.00 | 1.00 | -1.00 | 1.00 | 1.00 | 1.00 | 1.00 | 1.00 | 1.00 | 1.00 | 1.00 | 1.00 | 1.00 | 1.00 | 1.00 | 1.00 | 1.00 | -1.00 | -1.00 | 0.00 | 0.00 | -1.000.00 -1.00 0.00 1.00 0.00 -1.00 0.00 -1.00 -1.00 1.00 1.00 1.00 1.00 -1.00 1.00 |
| GYRATE_MN Permanent crops | 77 | -1.00 | -1.00 | 0.00 | 0.00 | -1.00 | -1.00 | -1.00 | -1.00 | 1.00 | 1.00 | -1.00 | 1.00 | -1.00 | -1.00 | 0.00 | 0.00 | 0.00 | 0.00 | 0.00 | 0.00 | 0.00 | 0.00 | 0.00 | 0.00 | 0.00 | 0.00 | 0.00 | 0.00 | 1.00 | 1.00 | 1.00 | 1.00 | -1.00 | 1.00 | -1.00 | 1.00 | -1.00 | 1.00 | 1.00 | 1.00 | -1.00 | 1.00 | 1.00 | 1.00 | 1.00 | 1.00 | 1.00 | 1.00 | 1.00 | 1.00 | 1.00 | 1.00 | 1.00 | 1.00 | 1.00 | 1.00 | 1.00 | 1.00 | 0.00 | 0.00 | 1.000.00 1.00 0.00 -1.00 0.00 1.00 0.00 1.00 1.00 -1.00 -1.00 -1.00 -1.00 1.00 -1.00 1.00 |
| GYRATE_SD Permanent crops | 78 | 1.00 | 1.00 | 0.00 | 0.00 | 1.00 | 1.00 | 1.00 | 1.00 | -1.00 | -1.00 | 1.00 | -1.00 | 1.00 | 1.00 | 0.00 | 0.00 | 0.00 | 0.00 | 0.00 | 0.00 | 0.00 | 0.00 | 0.00 | 0.00 | 0.00 | 0.00 | 0.00 | 0.00 | -1.00 | -1.00 | -1.00 | -1.00 | 1.00 | -1.00 | 1.00 | -1.00 | 1.00 | -1.00 | -1.00 | -1.00 | 1.00 | -1.00 | 1.00 | 1.00 | 1.00 | 1.00 | 1.00 | 1.00 | 1.00 | 1.00 | 1.00 | 1.00 | 1.00 | 1.00 | 1.00 | 1.00 | -1.00 | -1.00 | 0.00 | 0.00 | -1.000.00 -1.00 0.00 1.00 0.00 -1.00 0.00 -1.00 -1.00 1.00 1.00 1.00 1.00 -1.00 1.00 -1.00 1.00 |
| PARA_MN Permanent crops | 79 | 1.00 | 1.00 | 0.00 | 0.00 | 1.00 | 1.00 | 1.00 | 1.00 | -1.00 | -1.00 | 1.00 | -1.00 | 1.00 | 1.00 | 0.00 | 0.00 | 0.00 | 0.00 | 0.00 | 0.00 | 0.00 | 0.00 | 0.00 | 0.00 | 0.00 | 0.00 | 0.00 | 0.00 | -1.00 | -1.00 | -1.00 | -1.00 | 1.00 | -1.00 | 1.00 | -1.00 | 1.00 | -1.00 | -1.00 | -1.00 | 1.00 | -1.00 | 1.00 | 1.00 | 1.00 | 1.00 | 1.00 | 1.00 | 1.00 | 1.00 | 1.00 | 1.00 | 1.00 | 1.00 | 1.00 | 1.00 | -1.00 | -1.00 | 0.00 | 0.00 | -1.000.00 -1.00 0.00 1.00 0.00 -1.00 0.00 -1.00 -1.00 1.00 1.00 1.00 1.00 -1.00 1.00 -1.00 1.00 1.00 |
| PARA_SD Permanent crops | 80 | 1.00 | 1.00 | 0.00 | 0.00 | 1.00 | 1.00 | 1.00 | 1.00 | -1.00 | -1.00 | 1.00 | -1.00 | 1.00 | 1.00 | 0.00 | 0.00 | 0.00 | 0.00 | 0.00 | 0.00 | 0.00 | 0.00 | 0.00 | 0.00 | 0.00 | 0.00 | 0.00 | 0.00 | -1.00 | -1.00 | -1.00 | -1.00 | 1.00 | -1.00 | 1.00 | -1.00 | 1.00 | -1.00 | -1.00 | -1.00 | 1.00 | -1.00 | 1.00 | 1.00 | 1.00 | 1.00 | 1.00 | 1.00 | 1.00 | 1.00 | 1.00 | 1.00 | 1.00 | 1.00 | 1.00 | 1.00 | -1.00 | -1.00 | 0.00 | 0.00 | -1.000.00 -1.00 0.00 1.00 0.00 -1.00 0.00 -1.00 -1.00 1.00 1.00 1.00 1.00 -1.00 1.00 -1.00 1.00 1.00 1.00 |
| CONTIG_MN Permanent crops | 81 | -1.00 | -1.00 | 0.00 | 0.00 | -1.00 | -1.00 | -1.00 | -1.00 | 1.00 | 1.00 | -1.00 | 1.00 | -1.00 | -1.00 | 0.00 | 0.00 | 0.00 | 0.00 | 0.00 | 0.00 | 0.00 | 0.00 | 0.00 | 0.00 | 0.00 | 0.00 | 0.00 | 0.00 | 1.00 | 1.00 | 1.00 | 1.00 | -1.00 | 1.00 | -1.00 | 1.00 | -1.00 | 1.00 | 1.00 | 1.00 | -1.00 | 1.00 | 1.00 | 1.00 | 1.00 | 1.00 | 1.00 | 1.00 | 1.00 | 1.00 | 1.00 | 1.00 | 1.00 | 1.00 | 1.00 | 1.00 | 1.00 | 1.00 | 0.00 | 0.00 | 1.000.00 1.00 0.00 -1.00 0.00 1.00 0.00 1.00 1.00 -1.00 -1.00 -1.00 -1.00 1.00 -1.00 1.00 -1.00 -1.00 -1.00 1.00 |
| CONTIG_SD Permanent crops | 82 | 1.00 | 1.00 | 0.00 | 0.00 | 1.00 | 1.00 | 1.00 | 1.00 | -1.00 | -1.00 | 1.00 | -1.00 | 1.00 | 1.00 | 0.00 | 0.00 | 0.00 | 0.00 | 0.00 | 0.00 | 0.00 | 0.00 | 0.00 | 0.00 | 0.00 | 0.00 | 0.00 | 0.00 | -1.00 | -1.00 | -1.00 | -1.00 | 1.00 | -1.00 | 1.00 | -1.00 | 1.00 | -1.00 | -1.00 | -1.00 | 1.00 | -1.00 | 1.00 | 1.00 | 1.00 | 1.00 | 1.00 | 1.00 | 1.00 | 1.00 | 1.00 | 1.00 | 1.00 | 1.00 | 1.00 | 1.00 | -1.00 | -1.00 | 0.00 | 0.00 | -1.000.00 -1.00 0.00 1.00 0.00 -1.00 0.00 -1.00 -1.00 1.00 1.00 1.00 1.00 -1.00 1.00 -1.00 1.00 1.00 1.00 -1.00 1.00 |
| COHESION Permanent crops | 83 | 1.00 | 1.00 | 0.00 | 0.00 | 1.00 | 1.00 | 1.00 | 1.00 | -1.00 | -1.00 | 1.00 | -1.00 | 1.00 | 1.00 | 0.00 | 0.00 | 0.00 | 0.00 | 0.00 | 0.00 | 0.00 | 0.00 | 0.00 | 0.00 | 0.00 | 0.00 | 0.00 | 0.00 | -1.00 | -1.00 | -1.00 | -1.00 | 1.00 | -1.00 | 1.00 | -1.00 | 1.00 | -1.00 | -1.00 | -1.00 | 1.00 | -1.00 | 1.00 | 1.00 | 1.00 | 1.00 | 1.00 | 1.00 | 1.00 | 1.00 | 1.00 | 1.00 | 1.00 | 1.00 | 1.00 | 1.00 | -1.00 | -1.00 | 0.00 | 0.00 | -1.000.00 -1.00 0.00 1.00 0.00 -1.00 0.00 -1.00 -1.00 1.00 1.00 1.00 1.00 -1.00 1.00 -1.00 1.00 1.00 1.00 -1.00 1.00 1.00 |
| MESH Permanent crops | 84 | 1.00 | 1.00 | 0.00 | 0.00 | 1.00 | 1.00 | 1.00 | 1.00 | -1.00 | -1.00 | 1.00 | -1.00 | 1.00 | 1.00 | 0.00 | 0.00 | 0.00 | 0.00 | 0.00 | 0.00 | 0.00 | 0.00 | 0.00 | 0.00 | 0.00 | 0.00 | 0.00 | 0.00 | -1.00 | -1.00 | -1.00 | -1.00 | 1.00 | -1.00 | 1.00 | -1.00 | 1.00 | -1.00 | -1.00 | -1.00 | 1.00 | -1.00 | 1.00 | 1.00 | 1.00 | 1.00 | 1.00 | 1.00 | 1.00 | 1.00 | 1.00 | 1.00 | 1.00 | 1.00 | 1.00 | 1.00 | -1.00 | -1.00 | 0.00 | 0.00 | -1.000.00 -1.00 0.00 1.00 0.00 -1.00 0.00 -1.00 -1.00 1.00 1.00 1.00 1.00 -1.00 1.00 -1.00 1.00 1.00 1.00 -1.00 1.00 1.00 1.00 |

**Appendix S5:**

**Table S4**. Spearman correlation between 16 landscape metrics for each land class at 2000 m area of influence. We found 20 independent metrics at five classes: Pastures (PD, AREA_MN, PARA_MN, ENN_SD, and COHESION), Urban buildings (PD and AREA_SD), Water of bodies (CA and GYRATE_SD), Forests (CA, PD, AREA_MN, AREA_SD, ENN_MN, and ENN_SD), and Transitory crops (CA, PD, AREA_MN, PARA_MN and COHESION). The link for downloading the spreadsheet online is: https://docs.google.com/spreadsheets/d/1N3pfuOvuHY8I6MCeoah7X7CsHO6J4bsD6dAkwXfmwFI/edit?usp=sharing

| 2000 m buffer | Metric ID | 1 | 2 | 3 | 4 | 5 | 6 | 7 | 8 | 9 | 10 | 11 | 12 | 13 | 14 | 15 | 16 | 17 | 18 | 19 | 20 | 21 | 22 | 23 | 24 | 25 | 26 | 27 | 28 | 29 | 30 | 31 | 32 | 33 | 34 | 35 | 36 | 37 | 38 | 39 | 40 | 41 | 42 | 43 | 44 | 45 | 46 | 47 | 48 | 49 | 50 | 51 | 52 | 53 | 54 | 55 | 56 | 57 | 58 | 59 | 60 | 6162 63 64 65 66 67 68 69 70 71 72 73 74 75 76 77 78 79 80 81 82 83 84 |
| --- | --- | --- | --- | --- | --- | --- | --- | --- | --- | --- | --- | --- | --- | --- | --- | --- | --- | --- | --- | --- | --- | --- | --- | --- | --- | --- | --- | --- | --- | --- | --- | --- | --- | --- | --- | --- | --- | --- | --- | --- | --- | --- | --- | --- | --- | --- | --- | --- | --- | --- | --- | --- | --- | --- | --- | --- | --- | --- | --- | --- | --- | --- |
| NP Urban infrastructure | 1 |  |  |  |  |  |  |  |  |  |  |  |  |  |  |  |  |  |  |  |  |  |  |  |  |  |  |  |  |  |  |  |  |  |  |  |  |  |  |  |  |  |  |  |  |  |  |  |  |  |  |  |  |  |  |  |  |  |  |  |  |  |
| PD Urban infrastructure | 2 | 1.00 |  |  |  |  |  |  |  |  |  |  |  |  |  |  |  |  |  |  |  |  |  |  |  |  |  |  |  |  |  |  |  |  |  |  |  |  |  |  |  |  |  |  |  |  |  |  |  |  |  |  |  |  |  |  |  |  |  |  |  |  |
| AREA_SD Urban infrastructure | 3 | 0.55 | 0.55 |  |  |  |  |  |  |  |  |  |  |  |  |  |  |  |  |  |  |  |  |  |  |  |  |  |  |  |  |  |  |  |  |  |  |  |  |  |  |  |  |  |  |  |  |  |  |  |  |  |  |  |  |  |  |  |  |  |  |  |
| PARA_MN Urban infrastructure | 4 | 0.64 | 0.64 | 0.33 |  |  |  |  |  |  |  |  |  |  |  |  |  |  |  |  |  |  |  |  |  |  |  |  |  |  |  |  |  |  |  |  |  |  |  |  |  |  |  |  |  |  |  |  |  |  |  |  |  |  |  |  |  |  |  |  |  |  |
| MESH Urban infrastructure | 5 | 0.47 | 0.47 | 0.94 | 0.23 |  |  |  |  |  |  |  |  |  |  |  |  |  |  |  |  |  |  |  |  |  |  |  |  |  |  |  |  |  |  |  |  |  |  |  |  |  |  |  |  |  |  |  |  |  |  |  |  |  |  |  |  |  |  |  |  |  |
| NP Pastures | 6 | 0.68 | 0.68 | 0.46 | 0.20 | 0.47 |  |  |  |  |  |  |  |  |  |  |  |  |  |  |  |  |  |  |  |  |  |  |  |  |  |  |  |  |  |  |  |  |  |  |  |  |  |  |  |  |  |  |  |  |  |  |  |  |  |  |  |  |  |  |  |  |
| PD Pastures | 7 | 0.68 | 0.68 | 0.46 | 0.19 | 0.47 | 1.00 |  |  |  |  |  |  |  |  |  |  |  |  |  |  |  |  |  |  |  |  |  |  |  |  |  |  |  |  |  |  |  |  |  |  |  |  |  |  |  |  |  |  |  |  |  |  |  |  |  |  |  |  |  |  |  |
| AREA_MN Pastures | 8 | -0.38 | -0.38 | -0.34 | -0.21 | -0.30 | -0.73 | -0.73 |  |  |  |  |  |  |  |  |  |  |  |  |  |  |  |  |  |  |  |  |  |  |  |  |  |  |  |  |  |  |  |  |  |  |  |  |  |  |  |  |  |  |  |  |  |  |  |  |  |  |  |  |  |  |
| GYRATE_MN Pastures | 9 | -0.52 | -0.52 | -0.49 | -0.19 | -0.49 | -0.87 | -0.87 | 0.73 |  |  |  |  |  |  |  |  |  |  |  |  |  |  |  |  |  |  |  |  |  |  |  |  |  |  |  |  |  |  |  |  |  |  |  |  |  |  |  |  |  |  |  |  |  |  |  |  |  |  |  |  |  |
| GYRATE_SD Pastures | 10 | -0.43 | -0.43 | -0.25 | -0.26 | -0.22 | -0.61 | -0.61 | 0.82 | 0.47 |  |  |  |  |  |  |  |  |  |  |  |  |  |  |  |  |  |  |  |  |  |  |  |  |  |  |  |  |  |  |  |  |  |  |  |  |  |  |  |  |  |  |  |  |  |  |  |  |  |  |  |  |
| PARA_MN Pastures | 11 | 0.10 | 0.10 | -0.07 | 0.40 | -0.08 | 0.30 | 0.30 | -0.09 | -0.32 | 0.07 |  |  |  |  |  |  |  |  |  |  |  |  |  |  |  |  |  |  |  |  |  |  |  |  |  |  |  |  |  |  |  |  |  |  |  |  |  |  |  |  |  |  |  |  |  |  |  |  |  |  |  |
| CONTIG_MN Pastures | 12 | -0.10 | -0.10 | 0.08 | -0.41 | 0.08 | -0.28 | -0.28 | 0.05 | 0.32 | -0.10 | -1.00 |  |  |  |  |  |  |  |  |  |  |  |  |  |  |  |  |  |  |  |  |  |  |  |  |  |  |  |  |  |  |  |  |  |  |  |  |  |  |  |  |  |  |  |  |  |  |  |  |  |  |
| ENN_SD Pastures | 13 | -0.03 | -0.03 | -0.15 | 0.00 | -0.15 | 0.15 | 0.15 | -0.52 | -0.43 | -0.41 | -0.05 | 0.03 |  |  |  |  |  |  |  |  |  |  |  |  |  |  |  |  |  |  |  |  |  |  |  |  |  |  |  |  |  |  |  |  |  |  |  |  |  |  |  |  |  |  |  |  |  |  |  |  |  |
| COHESION Pastures | 14 | -0.18 | -0.18 | 0.06 | -0.32 | 0.14 | -0.32 | -0.32 | 0.76 | 0.35 | 0.82 | -0.05 | 0.03 | -0.71 |  |  |  |  |  |  |  |  |  |  |  |  |  |  |  |  |  |  |  |  |  |  |  |  |  |  |  |  |  |  |  |  |  |  |  |  |  |  |  |  |  |  |  |  |  |  |  |  |
| CA Bodies of water | 15 | -0.19 | -0.19 | -0.41 | -0.29 | -0.42 | -0.30 | -0.30 | 0.18 | 0.55 | -0.13 | -0.48 | 0.52 | -0.08 | -0.10 |  |  |  |  |  |  |  |  |  |  |  |  |  |  |  |  |  |  |  |  |  |  |  |  |  |  |  |  |  |  |  |  |  |  |  |  |  |  |  |  |  |  |  |  |  |  |  |
| AREA_MN Bodies of water | 16 | -0.07 | -0.07 | -0.45 | -0.29 | -0.40 | -0.26 | -0.26 | 0.14 | 0.54 | -0.19 | -0.62 | 0.64 | -0.01 | -0.10 | 0.84 |  |  |  |  |  |  |  |  |  |  |  |  |  |  |  |  |  |  |  |  |  |  |  |  |  |  |  |  |  |  |  |  |  |  |  |  |  |  |  |  |  |  |  |  |  |  |
| GYRATE_SD Bodies of water | 17 | -0.16 | -0.16 | 0.16 | -0.08 | -0.06 | -0.19 | -0.19 | 0.02 | 0.15 | 0.04 | -0.22 | 0.24 | -0.14 | -0.07 | 0.36 | -0.08 |  |  |  |  |  |  |  |  |  |  |  |  |  |  |  |  |  |  |  |  |  |  |  |  |  |  |  |  |  |  |  |  |  |  |  |  |  |  |  |  |  |  |  |  |  |
| CA Native forests | 18 | 0.52 | 0.52 | 0.16 | 0.40 | 0.10 | 0.60 | 0.60 | -0.32 | -0.41 | -0.10 | 0.66 | -0.63 | -0.25 | 0.01 | -0.34 | -0.31 | -0.20 |  |  |  |  |  |  |  |  |  |  |  |  |  |  |  |  |  |  |  |  |  |  |  |  |  |  |  |  |  |  |  |  |  |  |  |  |  |  |  |  |  |  |  |  |
| PLAND Native forests | 19 | 0.52 | 0.52 | 0.16 | 0.40 | 0.10 | 0.60 | 0.60 | -0.32 | -0.41 | -0.10 | 0.66 | -0.63 | -0.25 | 0.01 | -0.34 | -0.31 | -0.20 | 1.00 |  |  |  |  |  |  |  |  |  |  |  |  |  |  |  |  |  |  |  |  |  |  |  |  |  |  |  |  |  |  |  |  |  |  |  |  |  |  |  |  |  |  |  |
| PD Native forests | 20 | 0.56 | 0.56 | 0.23 | 0.83 | 0.16 | 0.29 | 0.29 | -0.17 | -0.26 | -0.08 | 0.52 | -0.52 | -0.10 | -0.14 | -0.37 | -0.33 | -0.20 | 0.72 | 0.72 |  |  |  |  |  |  |  |  |  |  |  |  |  |  |  |  |  |  |  |  |  |  |  |  |  |  |  |  |  |  |  |  |  |  |  |  |  |  |  |  |  |  |
| AREA_MN Native forests | 21 | 0.04 | 0.04 | 0.01 | -0.37 | 0.02 | 0.45 | 0.45 | -0.23 | -0.29 | -0.01 | 0.24 | -0.19 | -0.24 | 0.26 | -0.04 | -0.12 | 0.02 | 0.38 | 0.38 | -0.28 |  |  |  |  |  |  |  |  |  |  |  |  |  |  |  |  |  |  |  |  |  |  |  |  |  |  |  |  |  |  |  |  |  |  |  |  |  |  |  |  |  |
| AREA_SD Native forests | 22 | 0.30 | 0.30 | -0.04 | -0.03 | 0.02 | 0.43 | 0.43 | 0.00 | -0.16 | 0.15 | 0.40 | -0.35 | -0.35 | 0.37 | 0.15 | 0.05 | -0.13 | 0.68 | 0.68 | 0.23 | 0.64 |  |  |  |  |  |  |  |  |  |  |  |  |  |  |  |  |  |  |  |  |  |  |  |  |  |  |  |  |  |  |  |  |  |  |  |  |  |  |  |  |
| ENN_MN Native forests | 23 | -0.23 | -0.23 | 0.04 | -0.25 | 0.05 | -0.03 | -0.03 | 0.05 | -0.03 | 0.27 | -0.11 | 0.12 | -0.33 | 0.37 | -0.08 | -0.24 | 0.32 | -0.13 | -0.13 | -0.31 | 0.55 | 0.08 |  |  |  |  |  |  |  |  |  |  |  |  |  |  |  |  |  |  |  |  |  |  |  |  |  |  |  |  |  |  |  |  |  |  |  |  |  |  |  |
| ENN_SD Native forests | 24 | -0.07 | -0.07 | 0.01 | -0.09 | -0.06 | -0.30 | -0.30 | 0.20 | 0.33 | 0.41 | -0.32 | 0.35 | -0.20 | 0.26 | 0.40 | 0.20 | 0.54 | -0.02 | -0.02 | -0.04 | 0.06 | 0.31 | 0.26 |  |  |  |  |  |  |  |  |  |  |  |  |  |  |  |  |  |  |  |  |  |  |  |  |  |  |  |  |  |  |  |  |  |  |  |  |  |  |
| COHESION Native forests | 25 | 0.31 | 0.31 | 0.11 | -0.02 | 0.17 | 0.47 | 0.47 | -0.06 | -0.30 | 0.20 | 0.36 | -0.32 | -0.31 | 0.41 | 0.00 | -0.13 | -0.05 | 0.63 | 0.63 | 0.22 | 0.69 | 0.95 | 0.29 | 0.39 |  |  |  |  |  |  |  |  |  |  |  |  |  |  |  |  |  |  |  |  |  |  |  |  |  |  |  |  |  |  |  |  |  |  |  |  |  |
| CA Transitory crops | 26 | -0.39 | -0.39 | -0.32 | -0.09 | -0.38 | -0.30 | -0.30 | -0.36 | 0.09 | -0.40 | -0.31 | 0.31 | 0.71 | -0.74 | 0.17 | 0.23 | 0.11 | -0.51 | -0.51 | -0.28 | -0.38 | -0.71 | -0.16 | -0.17 | -0.72 |  |  |  |  |  |  |  |  |  |  |  |  |  |  |  |  |  |  |  |  |  |  |  |  |  |  |  |  |  |  |  |  |  |  |  |  |
| PLAND Transitory crops | 27 | -0.39 | -0.39 | -0.32 | -0.09 | -0.38 | -0.30 | -0.30 | -0.36 | 0.09 | -0.40 | -0.31 | 0.31 | 0.71 | -0.74 | 0.17 | 0.23 | 0.11 | -0.51 | -0.51 | -0.28 | -0.38 | -0.71 | -0.16 | -0.17 | -0.72 | 1.00 |  |  |  |  |  |  |  |  |  |  |  |  |  |  |  |  |  |  |  |  |  |  |  |  |  |  |  |  |  |  |  |  |  |  |  |
| NP Transitory crops | 28 | -0.41 | -0.41 | -0.34 | -0.12 | -0.30 | -0.60 | -0.60 | 0.08 | 0.54 | 0.15 | -0.46 | 0.48 | 0.20 | -0.17 | 0.36 | 0.43 | 0.15 | -0.47 | -0.47 | -0.30 | -0.26 | -0.31 | 0.09 | 0.55 | -0.28 | 0.51 | 0.51 |  |  |  |  |  |  |  |  |  |  |  |  |  |  |  |  |  |  |  |  |  |  |  |  |  |  |  |  |  |  |  |  |  |  |
| PD Transitory crops | 29 | -0.41 | -0.41 | -0.34 | -0.12 | -0.30 | -0.60 | -0.60 | 0.08 | 0.54 | 0.15 | -0.46 | 0.48 | 0.20 | -0.17 | 0.36 | 0.43 | 0.15 | -0.47 | -0.47 | -0.30 | -0.25 | -0.31 | 0.09 | 0.55 | -0.28 | 0.51 | 0.51 | 1.00 |  |  |  |  |  |  |  |  |  |  |  |  |  |  |  |  |  |  |  |  |  |  |  |  |  |  |  |  |  |  |  |  |  |
| AREA_MN Transitory crops | 30 | -0.37 | -0.37 | -0.33 | 0.03 | -0.39 | -0.05 | -0.05 | -0.46 | -0.10 | -0.46 | 0.06 | -0.05 | 0.47 | -0.74 | 0.10 | -0.04 | 0.26 | -0.19 | -0.19 | -0.07 | -0.14 | -0.48 | 0.13 | -0.26 | -0.47 | 0.78 | 0.78 | 0.20 | 0.20 |  |  |  |  |  |  |  |  |  |  |  |  |  |  |  |  |  |  |  |  |  |  |  |  |  |  |  |  |  |  |  |  |
| AREA_SD Transitory crops | 31 | -0.28 | -0.28 | -0.18 | -0.11 | -0.27 | -0.34 | -0.34 | -0.24 | 0.12 | -0.30 | -0.40 | 0.39 | 0.73 | -0.61 | 0.13 | 0.24 | 0.09 | -0.59 | -0.59 | -0.38 | -0.40 | -0.72 | -0.29 | -0.09 | -0.73 | 0.91 | 0.91 | 0.53 | 0.53 | 0.48 |  |  |  |  |  |  |  |  |  |  |  |  |  |  |  |  |  |  |  |  |  |  |  |  |  |  |  |  |  |  |  |
| GYRATE_MN Transitory crops | 32 | -0.35 | -0.35 | -0.36 | 0.16 | -0.41 | -0.07 | -0.07 | -0.42 | 0.02 | -0.40 | 0.26 | -0.23 | 0.26 | -0.67 | 0.05 | -0.05 | 0.16 | 0.03 | 0.03 | 0.11 | -0.08 | -0.33 | 0.10 | -0.25 | -0.36 | 0.65 | 0.65 | 0.22 | 0.22 | 0.93 | 0.32 |  |  |  |  |  |  |  |  |  |  |  |  |  |  |  |  |  |  |  |  |  |  |  |  |  |  |  |  |  |  |
| GYRATE_SD Transitory crops | 33 | -0.32 | -0.32 | -0.16 | -0.13 | -0.28 | -0.46 | -0.46 | -0.14 | 0.26 | -0.18 | -0.47 | 0.47 | 0.61 | -0.50 | 0.23 | 0.29 | 0.23 | -0.62 | -0.62 | -0.41 | -0.37 | -0.66 | -0.22 | 0.11 | -0.66 | 0.85 | 0.85 | 0.64 | 0.64 | 0.39 | 0.97 | 0.25 |  |  |  |  |  |  |  |  |  |  |  |  |  |  |  |  |  |  |  |  |  |  |  |  |  |  |  |  |  |
| PARA_MN Transitory crops | 34 | -0.27 | -0.27 | -0.08 | 0.01 | -0.24 | -0.55 | -0.55 | 0.17 | 0.52 | 0.22 | -0.21 | 0.24 | 0.00 | -0.05 | 0.39 | 0.17 | 0.58 | -0.32 | -0.32 | -0.26 | -0.04 | -0.11 | 0.11 | 0.68 | -0.08 | 0.27 | 0.27 | 0.69 | 0.69 | 0.04 | 0.40 | 0.07 | 0.59 |  |  |  |  |  |  |  |  |  |  |  |  |  |  |  |  |  |  |  |  |  |  |  |  |  |  |  |  |
| PARA_SD Transitory crops | 35 | -0.25 | -0.25 | 0.01 | -0.11 | -0.18 | -0.51 | -0.51 | 0.12 | 0.40 | 0.17 | -0.38 | 0.38 | 0.18 | -0.09 | 0.32 | 0.15 | 0.59 | -0.46 | -0.46 | -0.40 | -0.09 | -0.28 | 0.06 | 0.59 | -0.23 | 0.38 | 0.38 | 0.63 | 0.63 | 0.04 | 0.59 | -0.04 | 0.75 | 0.94 |  |  |  |  |  |  |  |  |  |  |  |  |  |  |  |  |  |  |  |  |  |  |  |  |  |  |  |
| CONTIG_MN Transitory crops | 36 | -0.41 | -0.41 | -0.39 | 0.20 | -0.45 | -0.41 | -0.41 | -0.17 | 0.36 | -0.07 | 0.13 | -0.08 | 0.18 | -0.47 | 0.23 | 0.10 | 0.26 | -0.08 | -0.08 | 0.05 | -0.09 | -0.18 | 0.14 | 0.29 | -0.19 | 0.56 | 0.56 | 0.68 | 0.68 | 0.62 | 0.38 | 0.75 | 0.45 | 0.63 | 0.44 |  |  |  |  |  |  |  |  |  |  |  |  |  |  |  |  |  |  |  |  |  |  |  |  |  |  |
| CONTIG_SD Transitory crops | 37 | -0.25 | -0.25 | -0.01 | -0.11 | -0.19 | -0.53 | -0.53 | 0.16 | 0.43 | 0.20 | -0.36 | 0.38 | 0.14 | -0.06 | 0.36 | 0.18 | 0.59 | -0.45 | -0.45 | -0.40 | -0.07 | -0.23 | 0.07 | 0.61 | -0.19 | 0.34 | 0.34 | 0.64 | 0.64 | 0.00 | 0.55 | -0.06 | 0.72 | 0.95 | 1.00 | 0.45 |  |  |  |  |  |  |  |  |  |  |  |  |  |  |  |  |  |  |  |  |  |  |  |  |  |
| COHESION Transitory crops | 38 | -0.41 | -0.41 | -0.38 | 0.20 | -0.45 | -0.42 | -0.42 | -0.16 | 0.37 | -0.07 | 0.12 | -0.08 | 0.17 | -0.46 | 0.23 | 0.10 | 0.27 | -0.09 | -0.09 | 0.04 | -0.09 | -0.19 | 0.14 | 0.30 | -0.19 | 0.56 | 0.56 | 0.69 | 0.69 | 0.61 | 0.39 | 0.74 | 0.46 | 0.65 | 0.46 | 1.00 | 0.47 |  |  |  |  |  |  |  |  |  |  |  |  |  |  |  |  |  |  |  |  |  |  |  |  |
| MESH Transitory crops | 39 | -0.31 | -0.31 | -0.27 | -0.11 | -0.31 | -0.16 | -0.16 | -0.38 | -0.09 | -0.42 | -0.22 | 0.20 | 0.85 | -0.75 | 0.08 | 0.13 | 0.04 | -0.54 | -0.54 | -0.34 | -0.35 | -0.68 | -0.25 | -0.27 | -0.68 | 0.94 | 0.94 | 0.38 | 0.38 | 0.69 | 0.92 | 0.49 | 0.82 | 0.18 | 0.35 | 0.38 | 0.31 | 0.38 |  |  |  |  |  |  |  |  |  |  |  |  |  |  |  |  |  |  |  |  |  |  |  |
| CONTIG_MN Native forest | 40 | -0.63 | -0.63 | 0.24 | 0.10 | -0.36 | -0.07 | -0.32 | 0.24 | 0.18 | 0.15 | -0.18 | 0.15 | -0.46 | -0.49 | -0.09 | -0.09 | -0.16 | -0.66 | -0.09 | -0.16 | -0.09 | -0.16 | 1.00 | -0.16 | -1.00 | -0.16 | -0.09 | -0.09 | 0.36 | 0.36 | -0.14 | -0.26 | 0.67 | -0.16 | 0.49 | -0.37 | -1.00 | -0.37 | 1.00 |  |  |  |  |  |  |  |  |  |  |  |  |  |  |  |  |  |  |  |  |  |  |
| CONTIG_SD Native forest | 41 | 0.36 | 0.36 | 0.13 | 0.20 | 0.03 | 0.18 | 0.08 | -0.01 | 0.09 | 0.10 | -0.09 | 0.10 | 0.12 | 0.15 | -0.49 | -0.49 | -0.40 | 0.17 | -0.49 | -0.40 | -0.49 | -0.40 | -0.81 | -0.40 | 0.81 | -0.40 | -0.49 | -0.49 | 0.64 | 0.64 | 0.86 | 0.81 | -0.01 | 0.92 | -0.39 | 0.98 | 0.37 | 1.00 | -0.37 | 1.00 |  |  |  |  |  |  |  |  |  |  |  |  |  |  |  |  |  |  |  |  |  |
| COHESION Native forest | 42 | -0.21 | -0.21 | 0.50 | 0.52 | -0.43 | 0.31 | -0.35 | 0.40 | 0.35 | 0.31 | -0.35 | 0.31 | -0.41 | -0.34 | -0.89 | -0.89 | -0.81 | -0.39 | -0.89 | -0.81 | -0.89 | -0.81 | -0.32 | -0.81 | 0.32 | -0.81 | -0.89 | -0.89 | 0.81 | 0.81 | 0.31 | 0.25 | 0.85 | 0.60 | 0.33 | 0.45 | -0.31 | 0.46 | 0.31 | 0.46 | 1.00 |  |  |  |  |  |  |  |  |  |  |  |  |  |  |  |  |  |  |  |  |
| MESH Native forest | 43 | -0.18 | -0.18 | 0.48 | 0.48 | -0.41 | 0.27 | -0.33 | 0.34 | 0.33 | 0.31 | -0.33 | 0.31 | -0.39 | -0.36 | -0.89 | -0.89 | -0.81 | -0.39 | -0.89 | -0.81 | -0.89 | -0.81 | -0.32 | -0.81 | 0.32 | -0.81 | -0.89 | -0.89 | 0.96 | 0.96 | 0.61 | 0.51 | 0.71 | 0.80 | 0.15 | 0.65 | -0.32 | 0.64 | 0.32 | 0.64 | 0.92 | 1.00 |  |  |  |  |  |  |  |  |  |  |  |  |  |  |  |  |  |  |  |
| CA Transitory crops | 44 | -0.72 | -0.72 | -0.20 | -0.11 | -0.23 | -0.78 | 0.10 | -0.78 | -0.20 | -0.31 | 0.20 | -0.31 | -0.31 | -0.38 | 1.00 | 1.00 | 1.00 | 1.00 | 1.00 | 1.00 | 1.00 | 1.00 | -1.00 | 1.00 | 1.00 | 1.00 | 1.00 | 1.00 | -0.07 | -0.07 | -0.35 | 0.27 | -0.07 | -0.35 | -0.19 | -0.35 | 0.43 | -0.35 | -0.43 | -0.35 | -0.07 | -0.07 | 1.00 |  |  |  |  |  |  |  |  |  |  |  |  |  |  |  |  |  |  |
| PLAND Transitory crops | 45 | -0.72 | -0.72 | -0.20 | -0.11 | -0.23 | -0.78 | 0.10 | -0.78 | -0.20 | -0.31 | 0.20 | -0.31 | -0.31 | -0.38 | 1.00 | 1.00 | 1.00 | 1.00 | 1.00 | 1.00 | 1.00 | 1.00 | -1.00 | 1.00 | 1.00 | 1.00 | 1.00 | 1.00 | -0.07 | -0.07 | -0.35 | 0.27 | -0.07 | -0.35 | -0.19 | -0.35 | 0.43 | -0.35 | -0.43 | -0.35 | -0.07 | -0.07 | 1.00 | 1.00 |  |  |  |  |  |  |  |  |  |  |  |  |  |  |  |  |  |
| NP Transitory crops | 46 | -0.11 | -0.11 | -0.27 | -0.22 | 0.02 | -0.42 | 0.20 | -0.42 | -0.20 | -0.27 | 0.20 | -0.27 | -0.08 | -0.08 | 1.00 | 1.00 | 1.00 | 1.00 | 1.00 | 1.00 | 1.00 | 1.00 | -1.00 | 1.00 | 1.00 | 1.00 | 1.00 | 1.00 | -0.56 | -0.56 | -0.47 | -0.05 | -0.56 | -0.47 | -0.45 | -0.47 | 0.67 | -0.47 | -0.67 | -0.47 | -0.56 | -0.56 | 0.62 | 0.62 | 1.00 |  |  |  |  |  |  |  |  |  |  |  |  |  |  |  |  |
| PD Transitory crops | 47 | -0.27 | -0.27 | -0.15 | -0.10 | -0.12 | -0.48 | 0.07 | -0.48 | -0.13 | -0.21 | 0.13 | -0.21 | -0.22 | -0.22 | 1.00 | 1.00 | 1.00 | 1.00 | 1.00 | 1.00 | 1.00 | 1.00 | -1.00 | 1.00 | 1.00 | 1.00 | 1.00 | 1.00 | -0.53 | -0.53 | -0.55 | -0.04 | -0.53 | -0.55 | -0.54 | -0.55 | 0.64 | -0.55 | -0.64 | -0.55 | -0.53 | -0.53 | 0.75 | 0.75 | 0.96 | 1.00 |  |  |  |  |  |  |  |  |  |  |  |  |  |  |  |
| AREA_MN Transitory crops | 48 | -0.47 | -0.47 | 0.22 | 0.22 | -0.26 | -0.17 | -0.19 | -0.17 | 0.14 | 0.17 | -0.14 | 0.17 | -0.23 | -0.33 | -1.00 | -1.00 | -1.00 | -1.00 | -1.00 | -1.00 | -1.00 | -1.00 | 1.00 | -1.00 | -1.00 | -1.00 | -1.00 | -1.00 | 0.75 | 0.75 | 0.53 | 0.52 | 0.75 | 0.53 | 0.53 | 0.53 | -0.52 | 0.53 | 0.52 | 0.53 | 0.75 | 0.75 | 0.03 | 0.03 | -0.73 | -0.61 | 1.00 |  |  |  |  |  |  |  |  |  |  |  |  |  |  |
| AREA_SD Transitory crops | 49 | -0.30 | -0.30 | -0.58 | -0.52 | 0.24 | -0.66 | 0.48 | -0.66 | -0.48 | -0.58 | 0.48 | -0.58 | 0.09 | 0.09 | -1.00 | -1.00 | -1.00 | -1.00 | -1.00 | -1.00 | -1.00 | -1.00 | 1.00 | -1.00 | -1.00 | -1.00 | -1.00 | -1.00 | -0.14 | -0.14 | -0.47 | -0.22 | -0.14 | -0.47 | 0.01 | -0.47 | 0.24 | -0.47 | -0.24 | -0.47 | -0.14 | -0.14 | 0.66 | 0.66 | 0.83 | 0.79 | -0.53 | 1.00 |  |  |  |  |  |  |  |  |  |  |  |  |  |
| GYRATE_MN Transitory crops | 50 | -0.56 | -0.56 | 0.35 | 0.37 | -0.42 | -0.05 | -0.34 | -0.05 | 0.31 | 0.33 | -0.31 | 0.33 | -0.39 | -0.45 | -1.00 | -1.00 | -1.00 | -1.00 | -1.00 | -1.00 | -1.00 | -1.00 | 1.00 | -1.00 | -1.00 | -1.00 | -1.00 | -1.00 | 0.75 | 0.75 | 0.53 | 0.52 | 0.75 | 0.53 | 0.53 | 0.53 | -0.52 | 0.53 | 0.52 | 0.53 | 0.75 | 0.75 | 0.00 | 0.00 | -0.73 | -0.61 | 0.97 | -0.53 | 1.00 |  |  |  |  |  |  |  |  |  |  |  |  |
| GYRATE_SD Transitory crops | 51 | -0.10 | -0.10 | -0.37 | -0.36 | 0.16 | -0.50 | 0.32 | -0.50 | -0.32 | -0.37 | 0.32 | -0.37 | 0.01 | 0.01 | -1.00 | -1.00 | -1.00 | -1.00 | -1.00 | -1.00 | -1.00 | -1.00 | 1.00 | -1.00 | -1.00 | -1.00 | -1.00 | -1.00 | -0.35 | -0.35 | -0.47 | -0.22 | -0.35 | -0.47 | -0.26 | -0.47 | 0.35 | -0.47 | -0.35 | -0.47 | -0.35 | -0.35 | 0.54 | 0.54 | 0.94 | 0.90 | -0.73 | 0.91 | -0.73 | 1.00 |  |  |  |  |  |  |  |  |  |  |  |
| PARA_MN Transitory crops | 52 | -0.36 | -0.36 | 0.03 | 0.07 | -0.30 | -0.36 | -0.11 | -0.36 | 0.08 | 0.00 | -0.08 | 0.00 | -0.42 | -0.39 | 1.00 | 1.00 | 1.00 | 1.00 | 1.00 | 1.00 | 1.00 | 1.00 | -1.00 | 1.00 | 1.00 | 1.00 | 1.00 | 1.00 | -0.33 | -0.33 | -0.35 | 0.11 | -0.33 | -0.35 | -0.38 | -0.35 | 0.47 | -0.35 | -0.47 | -0.35 | -0.33 | -0.33 | 0.66 | 0.66 | 0.91 | 0.95 | -0.60 | 0.77 | -0.54 | 0.89 | 1.00 |  |  |  |  |  |  |  |  |  |  |
| PARA_SD Transitory crops | 53 | -0.07 | -0.07 | -0.27 | -0.24 | 0.05 | -0.41 | 0.21 | -0.41 | -0.21 | -0.27 | 0.21 | -0.27 | -0.06 | -0.06 | 1.00 | 1.00 | 1.00 | 1.00 | 1.00 | 1.00 | 1.00 | 1.00 | -1.00 | 1.00 | 1.00 | 1.00 | 1.00 | 1.00 | -0.52 | -0.52 | -0.47 | -0.11 | -0.52 | -0.47 | -0.43 | -0.47 | 0.57 | -0.47 | -0.57 | -0.47 | -0.52 | -0.52 | 0.57 | 0.57 | 0.99 | 0.95 | -0.76 | 0.84 | -0.76 | 0.97 | 0.92 | 1.00 |  |  |  |  |  |  |  |  |  |
| CONTIG_MN Transitory crops | 54 | 0.33 | 0.33 | -0.01 | -0.06 | 0.28 | 0.33 | 0.10 | 0.33 | -0.10 | -0.01 | 0.10 | -0.01 | 0.41 | 0.38 | -1.00 | -1.00 | -1.00 | -1.00 | -1.00 | -1.00 | -1.00 | -1.00 | 1.00 | -1.00 | -1.00 | -1.00 | -1.00 | -1.00 | 0.30 | 0.30 | 0.27 | -0.15 | 0.30 | 0.27 | 0.30 | 0.27 | -0.45 | 0.27 | 0.45 | 0.27 | 0.30 | 0.30 | -0.61 | -0.61 | -0.92 | -0.93 | 0.63 | -0.77 | 0.55 | -0.90 | -0.99 | -0.93 | 1.00 |  |  |  |  |  |  |  |  |
| CONTIG_SD Transitory crops | 55 | -0.07 | -0.07 | -0.27 | -0.24 | 0.05 | -0.41 | 0.21 | -0.41 | -0.21 | -0.27 | 0.21 | -0.27 | -0.06 | -0.06 | 1.00 | 1.00 | 1.00 | 1.00 | 1.00 | 1.00 | 1.00 | 1.00 | -1.00 | 1.00 | 1.00 | 1.00 | 1.00 | 1.00 | -0.52 | -0.52 | -0.47 | -0.11 | -0.52 | -0.47 | -0.43 | -0.47 | 0.57 | -0.47 | -0.57 | -0.47 | -0.52 | -0.52 | 0.57 | 0.57 | 0.99 | 0.95 | -0.76 | 0.84 | -0.76 | 0.97 | 0.92 | 1.00 | -0.93 | 1.00 |  |  |  |  |  |  |  |
| COHESION Transitory crops | 56 | -0.56 | -0.56 | 0.17 | 0.14 | -0.23 | -0.32 | -0.12 | -0.32 | 0.07 | 0.12 | -0.07 | 0.12 | -0.26 | -0.38 | -1.00 | -1.00 | -1.00 | -1.00 | -1.00 | -1.00 | -1.00 | -1.00 | 1.00 | -1.00 | -1.00 | -1.00 | -1.00 | -1.00 | 0.86 | 0.86 | 0.53 | 0.52 | 0.86 | 0.53 | 0.61 | 0.53 | -0.63 | 0.53 | 0.63 | 0.53 | 0.86 | 0.86 | 0.11 | 0.11 | -0.64 | -0.51 | 0.97 | -0.38 | 0.94 | -0.58 | -0.48 | -0.65 | 0.51 | -0.65 | 1.00 |  |  |  |  |  |  |
| MESH Transitory crops | 57 | -0.78 | -0.78 | -0.14 | -0.08 | -0.23 | -0.81 | 0.06 | -0.81 | -0.20 | -0.28 | 0.20 | -0.28 | -0.35 | -0.42 | -1.00 | -1.00 | -1.00 | -1.00 | -1.00 | -1.00 | -1.00 | -1.00 | 1.00 | -1.00 | -1.00 | -1.00 | -1.00 | -1.00 | 0.13 | 0.13 | -0.35 | 0.16 | 0.13 | -0.35 | -0.12 | -0.35 | 0.13 | -0.35 | -0.13 | -0.35 | 0.13 | 0.13 | 0.94 | 0.94 | 0.48 | 0.66 | 0.14 | 0.61 | 0.11 | 0.49 | 0.60 | 0.46 | -0.54 | 0.46 | 0.26 | 1.00 |  |  |  |  |  |
| CA Urban infrastructure | 58 | -0.25 | -0.25 | 0.03 | 0.03 | -0.27 | -0.30 | -0.09 | -0.14 | 0.34 | 0.21 | -0.34 | 0.21 | -0.27 | -0.27 | 0.00 | 0.00 | 0.00 | 0.00 | 0.00 | 0.00 | 0.00 | 0.00 | 0.00 | 0.00 | 0.00 | 0.00 | 0.00 | 0.00 | 0.50 | 0.50 | 0.67 | 0.67 | -0.40 | 0.67 | -0.70 | 0.56 | 0.00 | 0.67 | 0.00 | 0.67 | -0.10 | 0.50 | 1.00 | 1.00 | 1.00 | 1.00 | 1.00 | 1.00 | 1.00 | 1.00 | 1.00 | 1.00 | 1.00 | 1.00 | 1.00 | 1.00 | 1.00 |  |  |  |  |
| PLAND Urban infrastructure | 59 | -0.25 | -0.25 | 0.03 | 0.03 | -0.27 | -0.30 | -0.09 | -0.14 | 0.34 | 0.21 | -0.34 | 0.21 | -0.27 | -0.27 | 0.00 | 0.00 | 0.00 | 0.00 | 0.00 | 0.00 | 0.00 | 0.00 | 0.00 | 0.00 | 0.00 | 0.00 | 0.00 | 0.00 | 0.50 | 0.50 | 0.67 | 0.67 | -0.40 | 0.67 | -0.70 | 0.56 | 0.00 | 0.67 | 0.00 | 0.67 | -0.10 | 0.50 | 1.00 | 1.00 | 1.00 | 1.00 | 1.00 | 1.00 | 1.00 | 1.00 | 1.00 | 1.00 | 1.00 | 1.00 | 1.00 | 1.00 | 1.00 | 1.00 |  |  |  |
| NP Urban infrastructure | 60 | 0.38 | 0.38 | -0.48 | -0.48 | 0.38 | -0.47 | 0.68 | -0.47 | -0.23 | -0.47 | 0.23 | -0.47 | 0.38 | 0.38 | 0.00 | 0.00 | 0.00 | 0.00 | 0.00 | 0.00 | 0.00 | 0.00 | 0.00 | 0.00 | 0.00 | 0.00 | 0.00 | 0.00 | -0.35 | -0.35 | 0.00 | 0.00 | -0.71 | 0.00 | -0.71 | 0.36 | 0.71 | 0.73 | -0.71 | 0.73 | -0.35 | -0.35 | 1.00 | 1.00 | 1.00 | 1.00 | 1.00 | 1.00 | 1.00 | 1.00 | 1.00 | 1.00 | 1.00 | 1.00 | 1.00 | 1.00 | 0.38 | 0.38 | 1.00 |  |  |
| PD Urban infrastructure | 61 | 0.38 | 0.38 | -0.48 | -0.48 | 0.38 | -0.47 | 0.68 | -0.47 | -0.23 | -0.47 | 0.23 | -0.47 | 0.38 | 0.38 | 0.00 | 0.00 | 0.00 | 0.00 | 0.00 | 0.00 | 0.00 | 0.00 | 0.00 | 0.00 | 0.00 | 0.00 | 0.00 | 0.00 | -0.35 | -0.35 | 0.00 | 0.00 | -0.71 | 0.00 | -0.71 | 0.36 | 0.71 | 0.73 | -0.71 | 0.73 | -0.35 | -0.35 | 1.00 | 1.00 | 1.00 | 1.00 | 1.00 | 1.00 | 1.00 | 1.00 | 1.00 | 1.00 | 1.00 | 1.00 | 1.00 | 1.00 | 0.38 | 0.38 | 1.00 | 1.00 |  |
| AREA_MN Urban infrastructure | 62 | -0.31 | -0.31 | 0.17 | 0.17 | -0.34 | -0.08 | -0.41 | 0.00 | 0.27 | 0.34 | -0.27 | 0.34 | -0.34 | -0.34 | 0.00 | 0.00 | 0.00 | 0.00 | 0.00 | 0.00 | 0.00 | 0.00 | 0.00 | 0.00 | 0.00 | 0.00 | 0.00 | 0.00 | 0.60 | 0.60 | 0.72 | 0.72 | -0.10 | 0.72 | -0.30 | 0.41 | -0.30 | 0.21 | 0.30 | 0.21 | -0.10 | 0.60 | 1.00 | 1.00 | 1.00 | 1.00 | 1.00 | 1.00 | 1.00 | 1.00 | 1.00 | 1.00 | 1.00 | 1.00 | 1.00 | 1.00 | 0.67 | 0.67 | -0.38 | -0.38 | 1.00 |
| AREA_SD Urban infrastructure | 63 | 0.38 | 0.38 | -0.48 | -0.48 | 0.38 | -0.47 | 0.68 | -0.47 | -0.23 | -0.47 | 0.23 | -0.47 | 0.38 | 0.38 | 0.00 | 0.00 | 0.00 | 0.00 | 0.00 | 0.00 | 0.00 | 0.00 | 0.00 | 0.00 | 0.00 | 0.00 | 0.00 | 0.00 | -0.35 | -0.35 | 0.00 | 0.00 | -0.71 | 0.00 | -0.71 | 0.36 | 0.71 | 0.73 | -0.71 | 0.73 | -0.35 | -0.35 | 1.00 | 1.00 | 1.00 | 1.00 | 1.00 | 1.00 | 1.00 | 1.00 | 1.00 | 1.00 | 1.00 | 1.00 | 1.00 | 1.00 | 0.38 | 0.38 | 1.00 | 1.00 | -0.381.00 |
| GYRATE_MN Urban infrastructure | 64 | -0.21 | -0.21 | 0.17 | 0.17 | -0.27 | -0.08 | -0.39 | 0.00 | 0.25 | 0.34 | -0.25 | 0.34 | -0.27 | -0.27 | 0.00 | 0.00 | 0.00 | 0.00 | 0.00 | 0.00 | 0.00 | 0.00 | 0.00 | 0.00 | 0.00 | 0.00 | 0.00 | 0.00 | 0.60 | 0.60 | 0.72 | 0.72 | -0.10 | 0.72 | -0.30 | 0.41 | -0.30 | 0.21 | 0.30 | 0.21 | -0.10 | 0.60 | 1.00 | 1.00 | 1.00 | 1.00 | 1.00 | 1.00 | 1.00 | 1.00 | 1.00 | 1.00 | 1.00 | 1.00 | 1.00 | 1.00 | 0.65 | 0.65 | -0.38 | -0.38 | 0.97-0.38 1.00 |
| GYRATE_SD Urban infrastructure | 65 | 0.38 | 0.38 | -0.48 | -0.48 | 0.38 | -0.47 | 0.68 | -0.47 | -0.23 | -0.47 | 0.23 | -0.47 | 0.38 | 0.38 | 0.00 | 0.00 | 0.00 | 0.00 | 0.00 | 0.00 | 0.00 | 0.00 | 0.00 | 0.00 | 0.00 | 0.00 | 0.00 | 0.00 | -0.35 | -0.35 | 0.00 | 0.00 | -0.71 | 0.00 | -0.71 | 0.36 | 0.71 | 0.73 | -0.71 | 0.73 | -0.35 | -0.35 | 1.00 | 1.00 | 1.00 | 1.00 | 1.00 | 1.00 | 1.00 | 1.00 | 1.00 | 1.00 | 1.00 | 1.00 | 1.00 | 1.00 | 0.38 | 0.38 | 1.00 | 1.00 | -0.381.00 -0.38 1.00 |
| PARA_MN Urban infrastructure | 66 | 0.06 | 0.06 | 0.07 | 0.07 | 0.09 | 0.07 | 0.16 | 0.19 | 0.01 | -0.04 | -0.01 | -0.04 | 0.09 | 0.09 | 0.00 | 0.00 | 0.00 | 0.00 | 0.00 | 0.00 | 0.00 | 0.00 | 0.00 | 0.00 | 0.00 | 0.00 | 0.00 | 0.00 | -0.50 | -0.50 | -0.82 | -0.82 | 0.30 | -0.82 | 0.40 | -0.62 | 0.00 | -0.31 | 0.00 | -0.31 | 0.20 | -0.50 | 1.00 | 1.00 | 1.00 | 1.00 | 1.00 | 1.00 | 1.00 | 1.00 | 1.00 | 1.00 | 1.00 | 1.00 | 1.00 | 1.00 | -0.42 | -0.42 | 0.38 | 0.38 | -0.860.38 -0.80 0.38 1.00 |
| PARA_SD Urban infrastructure | 67 | 0.38 | 0.38 | -0.48 | -0.48 | 0.38 | -0.47 | 0.68 | -0.47 | -0.23 | -0.47 | 0.23 | -0.47 | 0.38 | 0.38 | 0.00 | 0.00 | 0.00 | 0.00 | 0.00 | 0.00 | 0.00 | 0.00 | 0.00 | 0.00 | 0.00 | 0.00 | 0.00 | 0.00 | -0.35 | -0.35 | 0.00 | 0.00 | -0.71 | 0.00 | -0.71 | 0.36 | 0.71 | 0.73 | -0.71 | 0.73 | -0.35 | -0.35 | 1.00 | 1.00 | 1.00 | 1.00 | 1.00 | 1.00 | 1.00 | 1.00 | 1.00 | 1.00 | 1.00 | 1.00 | 1.00 | 1.00 | 0.38 | 0.38 | 1.00 | 1.00 | -0.381.00 -0.38 1.00 0.38 1.00 |
| CONTIG_MN Urban infrastructure | 68 | -0.06 | -0.06 | -0.07 | -0.07 | -0.09 | -0.07 | -0.16 | -0.19 | -0.01 | 0.04 | 0.01 | 0.04 | -0.09 | -0.09 | 0.00 | 0.00 | 0.00 | 0.00 | 0.00 | 0.00 | 0.00 | 0.00 | 0.00 | 0.00 | 0.00 | 0.00 | 0.00 | 0.00 | 0.50 | 0.50 | 0.82 | 0.82 | -0.30 | 0.82 | -0.40 | 0.62 | 0.00 | 0.31 | 0.00 | 0.31 | -0.20 | 0.50 | 1.00 | 1.00 | 1.00 | 1.00 | 1.00 | 1.00 | 1.00 | 1.00 | 1.00 | 1.00 | 1.00 | 1.00 | 1.00 | 1.00 | 0.42 | 0.42 | -0.38 | -0.38 | 0.86-0.38 0.80 -0.38 -1.00 -0.38 1.00 |
| CONTIG_SD Urban infrastructure | 69 | 0.38 | 0.38 | -0.48 | -0.48 | 0.38 | -0.47 | 0.68 | -0.47 | -0.23 | -0.47 | 0.23 | -0.47 | 0.38 | 0.38 | 0.00 | 0.00 | 0.00 | 0.00 | 0.00 | 0.00 | 0.00 | 0.00 | 0.00 | 0.00 | 0.00 | 0.00 | 0.00 | 0.00 | -0.35 | -0.35 | 0.00 | 0.00 | -0.71 | 0.00 | -0.71 | 0.36 | 0.71 | 0.73 | -0.71 | 0.73 | -0.35 | -0.35 | 1.00 | 1.00 | 1.00 | 1.00 | 1.00 | 1.00 | 1.00 | 1.00 | 1.00 | 1.00 | 1.00 | 1.00 | 1.00 | 1.00 | 0.38 | 0.38 | 1.00 | 1.00 | -0.381.00 -0.38 1.00 0.38 1.00 -0.38 1.00 |
| COHESION Urban infrastructure | 70 | -0.31 | -0.31 | 0.17 | 0.17 | -0.34 | -0.08 | -0.41 | 0.00 | 0.27 | 0.34 | -0.27 | 0.34 | -0.34 | -0.34 | 0.00 | 0.00 | 0.00 | 0.00 | 0.00 | 0.00 | 0.00 | 0.00 | 0.00 | 0.00 | 0.00 | 0.00 | 0.00 | 0.00 | 0.60 | 0.60 | 0.72 | 0.72 | -0.10 | 0.72 | -0.30 | 0.41 | -0.30 | 0.21 | 0.30 | 0.21 | -0.10 | 0.60 | 1.00 | 1.00 | 1.00 | 1.00 | 1.00 | 1.00 | 1.00 | 1.00 | 1.00 | 1.00 | 1.00 | 1.00 | 1.00 | 1.00 | 0.67 | 0.67 | -0.38 | -0.38 | 1.00-0.38 0.97 -0.38 -0.86 -0.38 0.86 -0.38 1.00 |
| MESH Urban infrastructure | 71 | -0.39 | -0.39 | 0.17 | 0.17 | -0.41 | -0.08 | -0.34 | 0.00 | 0.39 | 0.34 | -0.39 | 0.34 | -0.41 | -0.41 | 0.00 | 0.00 | 0.00 | 0.00 | 0.00 | 0.00 | 0.00 | 0.00 | 0.00 | 0.00 | 0.00 | 0.00 | 0.00 | 0.00 | 0.70 | 0.70 | 0.87 | 0.87 | -0.30 | 0.87 | -0.60 | 0.67 | -0.10 | 0.56 | 0.10 | 0.56 | 0.00 | 0.70 | 1.00 | 1.00 | 1.00 | 1.00 | 1.00 | 1.00 | 1.00 | 1.00 | 1.00 | 1.00 | 1.00 | 1.00 | 1.00 | 1.00 | 0.86 | 0.86 | -0.08 | -0.08 | 0.93-0.08 0.86 -0.08 -0.79 -0.08 0.79 -0.08 0.93 1.00 |
| CA Permanent crops | 72 | 1.00 | 1.00 | 0.00 | 0.00 | 1.00 | 1.00 | 1.00 | 1.00 | -1.00 | -1.00 | 1.00 | -1.00 | 1.00 | 1.00 | 0.00 | 0.00 | 0.00 | 0.00 | 0.00 | 0.00 | 0.00 | 0.00 | 0.00 | 0.00 | 0.00 | 0.00 | 0.00 | 0.00 | -1.00 | -1.00 | -1.00 | -1.00 | 1.00 | -1.00 | 1.00 | -1.00 | 1.00 | -1.00 | -1.00 | -1.00 | 1.00 | -1.00 | 1.00 | 1.00 | 1.00 | 1.00 | 1.00 | 1.00 | 1.00 | 1.00 | 1.00 | 1.00 | 1.00 | 1.00 | 1.00 | 1.00 | -1.00 | -1.00 | 0.00 | 0.00 | -1.000.00 -1.00 0.00 1.00 0.00 -1.00 0.00 -1.00 -1.00 1.00 |
| PLAND Permanent crops | 73 | 1.00 | 1.00 | 0.00 | 0.00 | 1.00 | 1.00 | 1.00 | 1.00 | -1.00 | -1.00 | 1.00 | -1.00 | 1.00 | 1.00 | 0.00 | 0.00 | 0.00 | 0.00 | 0.00 | 0.00 | 0.00 | 0.00 | 0.00 | 0.00 | 0.00 | 0.00 | 0.00 | 0.00 | -1.00 | -1.00 | -1.00 | -1.00 | 1.00 | -1.00 | 1.00 | -1.00 | 1.00 | -1.00 | -1.00 | -1.00 | 1.00 | -1.00 | 1.00 | 1.00 | 1.00 | 1.00 | 1.00 | 1.00 | 1.00 | 1.00 | 1.00 | 1.00 | 1.00 | 1.00 | 1.00 | 1.00 | -1.00 | -1.00 | 0.00 | 0.00 | -1.000.00 -1.00 0.00 1.00 0.00 -1.00 0.00 -1.00 -1.00 1.00 1.00 |
| NP Permanent crops | 74 | 1.00 | 1.00 | 0.00 | 0.00 | 1.00 | 1.00 | 1.00 | 1.00 | -1.00 | -1.00 | 1.00 | -1.00 | 1.00 | 1.00 | 0.00 | 0.00 | 0.00 | 0.00 | 0.00 | 0.00 | 0.00 | 0.00 | 0.00 | 0.00 | 0.00 | 0.00 | 0.00 | 0.00 | -1.00 | -1.00 | -1.00 | -1.00 | 1.00 | -1.00 | 1.00 | -1.00 | 1.00 | -1.00 | -1.00 | -1.00 | 1.00 | -1.00 | 1.00 | 1.00 | 1.00 | 1.00 | 1.00 | 1.00 | 1.00 | 1.00 | 1.00 | 1.00 | 1.00 | 1.00 | 1.00 | 1.00 | -1.00 | -1.00 | 0.00 | 0.00 | -1.000.00 -1.00 0.00 1.00 0.00 -1.00 0.00 -1.00 -1.00 1.00 1.00 1.00 |
| PD Permanent crops | 75 | 1.00 | 1.00 | 0.00 | 0.00 | 1.00 | 1.00 | 1.00 | 1.00 | -1.00 | -1.00 | 1.00 | -1.00 | 1.00 | 1.00 | 0.00 | 0.00 | 0.00 | 0.00 | 0.00 | 0.00 | 0.00 | 0.00 | 0.00 | 0.00 | 0.00 | 0.00 | 0.00 | 0.00 | -1.00 | -1.00 | -1.00 | -1.00 | 1.00 | -1.00 | 1.00 | -1.00 | 1.00 | -1.00 | -1.00 | -1.00 | 1.00 | -1.00 | 1.00 | 1.00 | 1.00 | 1.00 | 1.00 | 1.00 | 1.00 | 1.00 | 1.00 | 1.00 | 1.00 | 1.00 | 1.00 | 1.00 | -1.00 | -1.00 | 0.00 | 0.00 | -1.000.00 -1.00 0.00 1.00 0.00 -1.00 0.00 -1.00 -1.00 1.00 1.00 1.00 1.00 |
| AREA_MN Permanent crops | 76 | -1.00 | -1.00 | 0.00 | 0.00 | -1.00 | -1.00 | -1.00 | -1.00 | 1.00 | 1.00 | -1.00 | 1.00 | -1.00 | -1.00 | 0.00 | 0.00 | 0.00 | 0.00 | 0.00 | 0.00 | 0.00 | 0.00 | 0.00 | 0.00 | 0.00 | 0.00 | 0.00 | 0.00 | 1.00 | 1.00 | 1.00 | 1.00 | -1.00 | 1.00 | -1.00 | 1.00 | -1.00 | 1.00 | 1.00 | 1.00 | -1.00 | 1.00 | 1.00 | 1.00 | 1.00 | 1.00 | 1.00 | 1.00 | 1.00 | 1.00 | 1.00 | 1.00 | 1.00 | 1.00 | 1.00 | 1.00 | 1.00 | 1.00 | 0.00 | 0.00 | 1.000.00 1.00 0.00 -1.00 0.00 1.00 0.00 1.00 1.00 -1.00 -1.00 -1.00 -1.00 1.00 |
| AREA_SD Permanent crops | 77 | 1.00 | 1.00 | 0.00 | 0.00 | 1.00 | 1.00 | 1.00 | 1.00 | -1.00 | -1.00 | 1.00 | -1.00 | 1.00 | 1.00 | 0.00 | 0.00 | 0.00 | 0.00 | 0.00 | 0.00 | 0.00 | 0.00 | 0.00 | 0.00 | 0.00 | 0.00 | 0.00 | 0.00 | -1.00 | -1.00 | -1.00 | -1.00 | 1.00 | -1.00 | 1.00 | -1.00 | 1.00 | -1.00 | -1.00 | -1.00 | 1.00 | -1.00 | 1.00 | 1.00 | 1.00 | 1.00 | 1.00 | 1.00 | 1.00 | 1.00 | 1.00 | 1.00 | 1.00 | 1.00 | 1.00 | 1.00 | -1.00 | -1.00 | 0.00 | 0.00 | -1.000.00 -1.00 0.00 1.00 0.00 -1.00 0.00 -1.00 -1.00 1.00 1.00 1.00 1.00 -1.00 1.00 |
| GYRATE_MN Permanent crops | 78 | -1.00 | -1.00 | 0.00 | 0.00 | -1.00 | -1.00 | -1.00 | -1.00 | 1.00 | 1.00 | -1.00 | 1.00 | -1.00 | -1.00 | 0.00 | 0.00 | 0.00 | 0.00 | 0.00 | 0.00 | 0.00 | 0.00 | 0.00 | 0.00 | 0.00 | 0.00 | 0.00 | 0.00 | 1.00 | 1.00 | 1.00 | 1.00 | -1.00 | 1.00 | -1.00 | 1.00 | -1.00 | 1.00 | 1.00 | 1.00 | -1.00 | 1.00 | 1.00 | 1.00 | 1.00 | 1.00 | 1.00 | 1.00 | 1.00 | 1.00 | 1.00 | 1.00 | 1.00 | 1.00 | 1.00 | 1.00 | 1.00 | 1.00 | 0.00 | 0.00 | 1.000.00 1.00 0.00 -1.00 0.00 1.00 0.00 1.00 1.00 -1.00 -1.00 -1.00 -1.00 1.00 -1.00 1.00 |
| GYRATE_SD Permanent crops | 79 | 1.00 | 1.00 | 0.00 | 0.00 | 1.00 | 1.00 | 1.00 | 1.00 | -1.00 | -1.00 | 1.00 | -1.00 | 1.00 | 1.00 | 0.00 | 0.00 | 0.00 | 0.00 | 0.00 | 0.00 | 0.00 | 0.00 | 0.00 | 0.00 | 0.00 | 0.00 | 0.00 | 0.00 | -1.00 | -1.00 | -1.00 | -1.00 | 1.00 | -1.00 | 1.00 | -1.00 | 1.00 | -1.00 | -1.00 | -1.00 | 1.00 | -1.00 | 1.00 | 1.00 | 1.00 | 1.00 | 1.00 | 1.00 | 1.00 | 1.00 | 1.00 | 1.00 | 1.00 | 1.00 | 1.00 | 1.00 | -1.00 | -1.00 | 0.00 | 0.00 | -1.000.00 -1.00 0.00 1.00 0.00 -1.00 0.00 -1.00 -1.00 1.00 1.00 1.00 1.00 -1.00 1.00 -1.00 1.00 |
| PARA_MN Permanent crops | 80 | 1.00 | 1.00 | 0.00 | 0.00 | 1.00 | 1.00 | 1.00 | 1.00 | -1.00 | -1.00 | 1.00 | -1.00 | 1.00 | 1.00 | 0.00 | 0.00 | 0.00 | 0.00 | 0.00 | 0.00 | 0.00 | 0.00 | 0.00 | 0.00 | 0.00 | 0.00 | 0.00 | 0.00 | -1.00 | -1.00 | -1.00 | -1.00 | 1.00 | -1.00 | 1.00 | -1.00 | 1.00 | -1.00 | -1.00 | -1.00 | 1.00 | -1.00 | 1.00 | 1.00 | 1.00 | 1.00 | 1.00 | 1.00 | 1.00 | 1.00 | 1.00 | 1.00 | 1.00 | 1.00 | 1.00 | 1.00 | -1.00 | -1.00 | 0.00 | 0.00 | -1.000.00 -1.00 0.00 1.00 0.00 -1.00 0.00 -1.00 -1.00 1.00 1.00 1.00 1.00 -1.00 1.00 -1.00 1.00 1.00 |
| PARA_SD Permanent crops | 81 | 1.00 | 1.00 | 0.00 | 0.00 | 1.00 | 1.00 | 1.00 | 1.00 | -1.00 | -1.00 | 1.00 | -1.00 | 1.00 | 1.00 | 0.00 | 0.00 | 0.00 | 0.00 | 0.00 | 0.00 | 0.00 | 0.00 | 0.00 | 0.00 | 0.00 | 0.00 | 0.00 | 0.00 | -1.00 | -1.00 | -1.00 | -1.00 | 1.00 | -1.00 | 1.00 | -1.00 | 1.00 | -1.00 | -1.00 | -1.00 | 1.00 | -1.00 | 1.00 | 1.00 | 1.00 | 1.00 | 1.00 | 1.00 | 1.00 | 1.00 | 1.00 | 1.00 | 1.00 | 1.00 | 1.00 | 1.00 | -1.00 | -1.00 | 0.00 | 0.00 | -1.000.00 -1.00 0.00 1.00 0.00 -1.00 0.00 -1.00 -1.00 1.00 1.00 1.00 1.00 -1.00 1.00 -1.00 1.00 1.00 1.00 |
| CONTIG_MN Permanent crops | 82 | -1.00 | -1.00 | 0.00 | 0.00 | -1.00 | -1.00 | -1.00 | -1.00 | 1.00 | 1.00 | -1.00 | 1.00 | -1.00 | -1.00 | 0.00 | 0.00 | 0.00 | 0.00 | 0.00 | 0.00 | 0.00 | 0.00 | 0.00 | 0.00 | 0.00 | 0.00 | 0.00 | 0.00 | 1.00 | 1.00 | 1.00 | 1.00 | -1.00 | 1.00 | -1.00 | 1.00 | -1.00 | 1.00 | 1.00 | 1.00 | -1.00 | 1.00 | 1.00 | 1.00 | 1.00 | 1.00 | 1.00 | 1.00 | 1.00 | 1.00 | 1.00 | 1.00 | 1.00 | 1.00 | 1.00 | 1.00 | 1.00 | 1.00 | 0.00 | 0.00 | 1.000.00 1.00 0.00 -1.00 0.00 1.00 0.00 1.00 1.00 -1.00 -1.00 -1.00 -1.00 1.00 -1.00 1.00 -1.00 -1.00 -1.00 1.00 |
| CONTIG_SD Permanent crops | 83 | 1.00 | 1.00 | 0.00 | 0.00 | 1.00 | 1.00 | 1.00 | 1.00 | -1.00 | -1.00 | 1.00 | -1.00 | 1.00 | 1.00 | 0.00 | 0.00 | 0.00 | 0.00 | 0.00 | 0.00 | 0.00 | 0.00 | 0.00 | 0.00 | 0.00 | 0.00 | 0.00 | 0.00 | -1.00 | -1.00 | -1.00 | -1.00 | 1.00 | -1.00 | 1.00 | -1.00 | 1.00 | -1.00 | -1.00 | -1.00 | 1.00 | -1.00 | 1.00 | 1.00 | 1.00 | 1.00 | 1.00 | 1.00 | 1.00 | 1.00 | 1.00 | 1.00 | 1.00 | 1.00 | 1.00 | 1.00 | -1.00 | -1.00 | 0.00 | 0.00 | -1.000.00 -1.00 0.00 1.00 0.00 -1.00 0.00 -1.00 -1.00 1.00 1.00 1.00 1.00 -1.00 1.00 -1.00 1.00 1.00 1.00 -1.00 1.00 |
| COHESION Permanent crops | 84 | 1.00 | 1.00 | 0.00 | 0.00 | 1.00 | 1.00 | 1.00 | 1.00 | -1.00 | -1.00 | 1.00 | -1.00 | 1.00 | 1.00 | 0.00 | 0.00 | 0.00 | 0.00 | 0.00 | 0.00 | 0.00 | 0.00 | 0.00 | 0.00 | 0.00 | 0.00 | 0.00 | 0.00 | -1.00 | -1.00 | -1.00 | -1.00 | 1.00 | -1.00 | 1.00 | -1.00 | 1.00 | -1.00 | -1.00 | -1.00 | 1.00 | -1.00 | 1.00 | 1.00 | 1.00 | 1.00 | 1.00 | 1.00 | 1.00 | 1.00 | 1.00 | 1.00 | 1.00 | 1.00 | 1.00 | 1.00 | -1.00 | -1.00 | 0.00 | 0.00 | -1.000.00 -1.00 0.00 1.00 0.00 -1.00 0.00 -1.00 -1.00 1.00 1.00 1.00 1.00 -1.00 1.00 -1.00 1.00 1.00 1.00 -1.00 1.00 1.00 |
| MESH Permanent crops | 85 | 1.00 | 1.00 | 0.00 | 0.00 | 1.00 | 1.00 | 1.00 | 1.00 | -1.00 | -1.00 | 1.00 | -1.00 | 1.00 | 1.00 | 0.00 | 0.00 | 0.00 | 0.00 | 0.00 | 0.00 | 0.00 | 0.00 | 0.00 | 0.00 | 0.00 | 0.00 | 0.00 | 0.00 | -1.00 | -1.00 | -1.00 | -1.00 | 1.00 | -1.00 | 1.00 | -1.00 | 1.00 | -1.00 | -1.00 | -1.00 | 1.00 | -1.00 | 1.00 | 1.00 | 1.00 | 1.00 | 1.00 | 1.00 | 1.00 | 1.00 | 1.00 | 1.00 | 1.00 | 1.00 | 1.00 | 1.00 | -1.00 | -1.00 | 0.00 | 0.00 | -1.000.00 -1.00 0.00 1.00 0.00 -1.00 0.00 -1.00 -1.00 1.00 1.00 1.00 1.00 -1.00 1.00 -1.00 1.00 1.00 1.00 -1.00 1.00 1.00 1.00 |

**Appendix S6:**


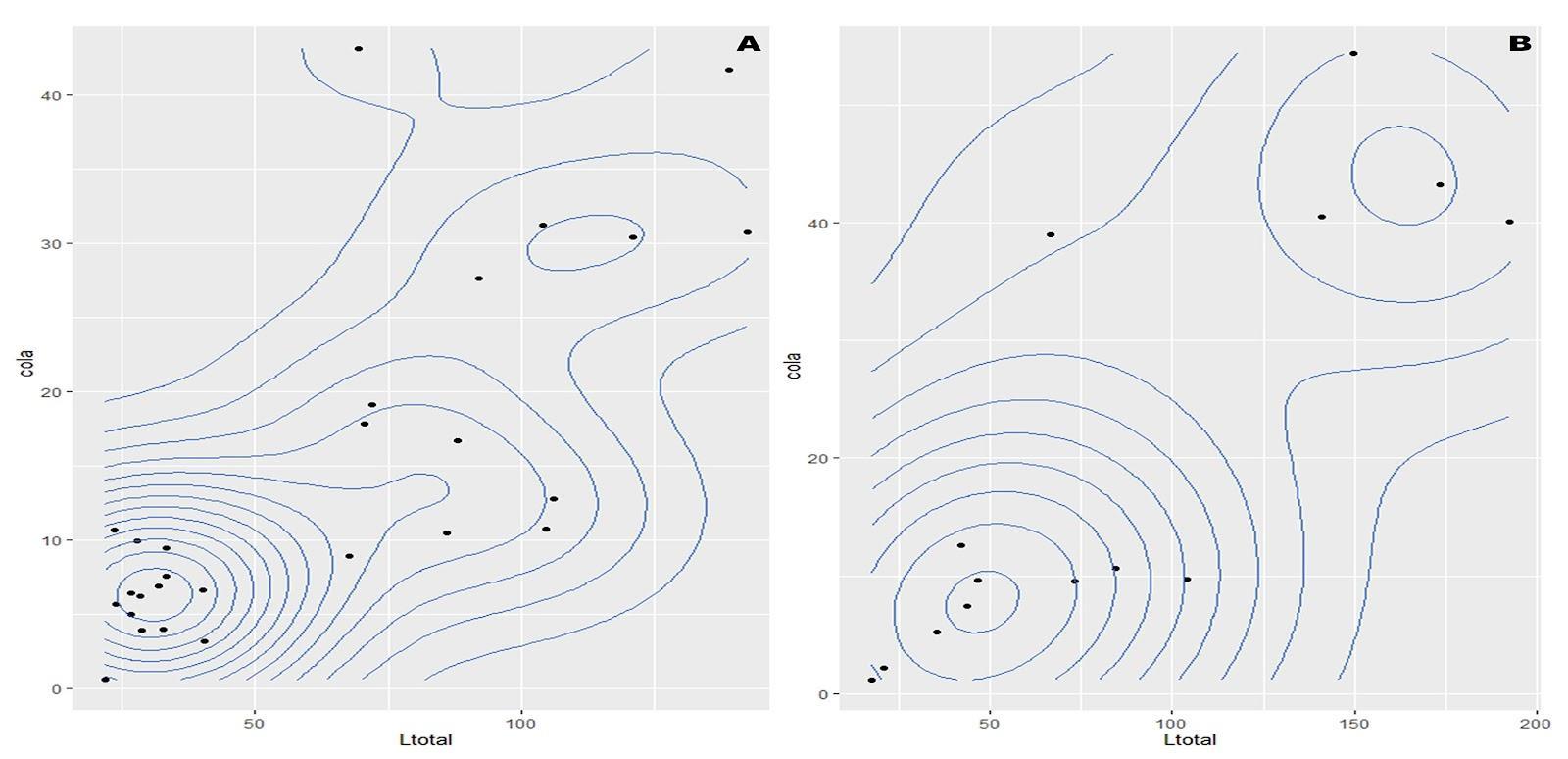


**Figure S2.** Inter-species variability of snakes based on two functional traits: LTotal = Total length; Cola = Tail length. **A.** killed on the highway; and **B.** alive on adjacent vegetation  in the Colombian Orinoco Region.

**Appendix S7:**

**Table S5**. Scale of the effect of habitat amount (NP forest landscape metric) on functional diversity indices of road-killed snakes and those inhabiting surrounding vegetation. The scale of the effect is that area of influence at which the R2 value of the linear regression between the amount of native forest and the index of functional diversity evaluated is greater (highlighted in bold).

|  |  | ***Area of influence of habitat amount (CA forest)*** | | | |
| --- | --- | --- | --- | --- | --- |
| **INDEX** | **Site** | **250** | **500** | **1000** | **2000** |
| Redundancy | Killed on road | **0.059** | 0,016684 | 0,0012605 | 0,011602 |
|  | Inhabiting adjacent vegetation | 0.119 | 0.023 | 0.140 | **0.270** |
| Fric | Killed on road | **0.434** | 0.280 | 0.017 | 0.097 |
|  | Inhabiting adjacent vegetation | **0.312** | 0.234 | 0.013 | 0.001 |
| Feve | Killed on road | 0.005 | 0.033 | **0.057** | 0.048 |
|  | Inhabiting adjacent vegetation | 0.000 | 0.006 | 0.051 | **0.082** |
| FDiv | Killed on road | 0.039 | **0.055** | 0.033 | 0.020 |
|  | Inhabiting adjacent vegetation | **0.196** | 0.043 | 0.100 | 0.089 |

**Appendix S8:**

**Table S6**. Best fitted models explaining changes in functional diversity indices on the road and adjacent coverages at four areas of influence (from 250 to 3000 m of measured spatial distances from the sampled site). For the best fitted models we report for each variable the pseudo F test statistic, its p-value and its percentage of explanation. The link for downloading the spreadsheet online is: <https://docs.google.com/spreadsheets/d/1N3pfuOvuHY8I6MCeoah7X7CsHO6J4bsD6dAkwXfmwFI/edit?usp=sharing>

|  |  |  |  |  |  |  | **MARGINAL TESTS** | | | | | | | | | | | | | | |
| --- | --- | --- | --- | --- | --- | --- | --- | --- | --- | --- | --- | --- | --- | --- | --- | --- | --- | --- | --- | --- | --- |
|  |  |  |  |  |  |  | **Variable #1** | | | | | **Variable #2** | | | | | **Variable #3** | | | | |
| **Location** | **Variable** | **Scale** | **AICc** | **R^2** | **RSS** | **No.Vars** | **Variable name** | **SS(trace)** | **Pseudo-F** | **P** | **Prop. (%)** | **Variable name** | **SS(trace)** | **Pseudo-F** | **P** | **Prop. (%)** | **Variable name** | **SS(trace)** | **Pseudo-F** | **P** | **Prop. (%)** |
| Run over on road | FRed | 250 | 14.512 | 0.70957 | 14.604 | 3 | CA Forest | 0.00029758 | 6.51E-05 | 0.9935 | 5.92E-06 | COHESION Forest | 8.6758 | 2.29E+00 | 0.16 | 1.73E-01 | CA permanent crops | 5.47E+00 | 1.3415 | 3.03E-01 | 0.1087 |
|  |  | 500 | 19.36 | 0.23164 | 38.636 | 1 | PD forest | 11.647 | 3.3161 | 0.0902 | 0.23164 |  |  |  |  |  |  |  |  |  |  |
|  |  | 1000 | 18.472 | 0.28234 | 36.086 | 1 | PARA_MN bodies of water | 14.197 | 4.3276 | 0.0606 | 0.28234 |  |  |  |  |  |  |  |  |  |  |
|  |  | 2000 | 16.422 | 0.38705 | 30.821 | 1 | ENN_SD pastures | 19.462 | 6.946 | 0.028 | 0.38705 |  |  |  |  |  |  |  |  |  |  |
|  | Fric | 250 | 102.14 | 0.43378 | 22522 | 1 | CA forest | 17254 | 8.427 | 0.0189 | 0.43378 |  |  |  |  |  |  |  |  |  |  |
|  |  | 500 | 105.27 | 0.27998 | 28639 | 1 | PLAND forest | 11136 | 4.2773 | 0.0648 | 0.27998 |  |  |  |  |  |  |  |  |  |  |
|  |  | 1000 | 106.25 | 0.22334 | 30892 | 1 | CONTIG_MN pasture | 8883.6 | 3.1633 | 0.1056 | 0.22334 |  |  |  |  |  |  |  |  |  |  |
|  |  | 2000 | 108.15 | 0.31165 | 27379 | 2 | AREA_MN pasture | 1381.7 | 0.39585 | 0.54 | 0.034736 | ENN_SD pasture | 5109.2 | 1.6212 | 0.22 | 0.12845 |  |  |  |  |  |
|  | Feve | 250 | -67.455 | 0.70035 | 0.026678 | 3 | COHESION pasture | 0.011525 | 1.6358 | 0.2256 | 0.12946 | PARA_MN forest | 3.88E-05 | 0.0047982 | 0.9494 | 0.00043601 | CA permanent crops | 0.031122 | 5.9121 | 0.0746 | 0.34958 |
|  |  | 500 | -61.009 | 0.31333 | 0.061133 | 2 | NP forest | 0.024834 | 4.2554 | 0.05 | 0.27894 | PD forest | 2.48E-02 | 4.2564 | 0.0678 | 0.27899 |  |  |  |  |  |
|  |  | 1000 | -63.965 | 0.28581 | 0.063583 | 1 | PARA_MN bodies of water | 0.025446 | 4.4022 | 0.0647 | 0.28581 |  |  |  |  |  |  |  |  |  |  |
|  |  | 2000 | -66.81 | 0.56051 | 0.039127 | 2 | PD_pasture | 0.04313 | 10.337 | 0.006 | 0.48445 | PD_Transit_crop | 0.036982 | 7.8162 | 0.014 | 0.4154 |  |  |  |  |  |
|  | FDiv | 250 | -74.012 | 0.75524 | 0.01611 | 3 | COHESION pastures | 0.0082766 | 1.5822 | 0.1787 | 0.12575 | CA transitory crops | 4.01E-05 | 0.0067139 | 0.9402 | 0.00060998 | CA permanent crops | 0.027195 | 7.7452 | 0.0761 | 0.41318 |
|  |  | 500 | -67.232 | 0.24866 | 0.049452 | 1 | PD forest | 0.016367 | 3.64E+00 | 0.08 | 0.24866 |  |  |  |  |  |  |  |  |  |  |
|  |  | 1000 | -70.758 | 0.42715 | 0.037704 | 1 | PARA_MN bodies of water | 0.028115 | 8.2023 | 0.0148 | 0.42715 |  |  |  |  |  |  |  |  |  |  |
|  |  | 2000 | -76.783 | 0.80222 | 0.013018 | 3 | PD transitory crops | 0.02447 | 6.5097 | 0.022 | 0.37178 | ENN_SD pastures | 0.0057946 | 1.0619 | 0.287 | 0.088039 | GYRATE_SD bodies of water | 0.0079553 | 1.5123 | 0.252 | 0.12087 |
| Living in adjacent vegetation | FRed | 250 | 5.1458 | 0.35855 | 9.4473 | 1 | PARA_MN forest | 5.2808 | 4.4717 | 0.0562 | 0.35855 |  |  |  |  |  |  |  |  |  |  |
|  |  | 500 | 8.3399 | 0.11716 | 13.003 | 1 | PD forest | 1.7256 | 1.0617 | 0.3494 | 0.11716 |  |  |  |  |  |  |  |  |  |  |
|  |  | 1000 | 6.674 | 0.25263 | 11.007 | 1 | AREA_SD pastures | 3.7208 | 2.7042 | 0.1358 | 0.25263 |  |  |  |  |  |  |  |  |  |  |
|  |  | 2000 | -0.016404 | 0.6172 | 5.6379 | 1 | AREA_MN forest | 9.0902 | 12.899 | 0.012 | 0.6172 |  |  |  |  |  |  |  |  |  |  |
|  | Fric | 250 | 84.988 | 0.62651 | 18059 | 2 | COHESION pasture | 17864 | 4.6875 | 0.049 | 0.36946 | CA forest | 15105 | 3.6348 | 0.0878 | 0.31241 |  |  |  |  |  |
|  |  | 500 | 87.891 | 0.23354 | 37059 | 1 | PLAND forest | 11292 | 2.4376 | 0.1515 | 0.23354 |  |  |  |  |  |  |  |  |  |  |
|  |  | 1000 | 87.259 | 0.28047 | 34790 | 1 | AREA_SD pastures | 13561 | 3.1183 | 0.1177 | 0.28047 |  |  |  |  |  |  |  |  |  |  |
|  |  | 2000 | 85.909 | 0.59047 | 19801 | 2 | AREA_SD urban infrastructure | 7490.3 | 1.4665 | 0.245 | 0.15491 | ENN_MN forest | 12659 | 2.8372 | 0.12 | 0.2618 |  |  |  |  |  |
|  | Feve | 250 | -38.896 | 0.2805 | 0.11551 | 1 | PARA_MN forest | 0.04503 | 3.1188 | 0.146 | 0.2805 |  |  |  |  |  |  |  |  |  |  |
|  |  | 500 | -37.174 | 0.14528 | 0.13722 | 1 | CA bodies of water | 0.023322 | 1.3598 | 0.3063 | 0.14528 |  |  |  |  |  |  |  |  |  |  |
|  |  | 1000 | -42.477 | 0.49709 | 0.080736 | 1 | AREA_SD pastures | 0.079802 | 7.9074 | 0.0269 | 0.49709 |  |  |  |  |  |  |  |  |  |  |
|  |  | 2000 | -56.606 | 0.92025 | 0.012804 | 2 | AREA_MN forest | 0.10028 | 13.312 | 0.019 | 0.62463 | ENN_SD forest | 0.090868 | 10.434 | 0.013 | 0.56603 |  |  |  |  |  |
|  | FDiv | 250 | -41.976 | 0.29227 | 0.084884 | 1 | PARA_MN forest | 0.035054 | 3.3037 | 0.1303 | 0.29227 |  |  |  |  |  |  |  |  |  |  |
|  |  | 500 | -39.237 | 0.069227 | 0.11164 | 1 | CA pastures | 0.0083029 | 0.595 | 0.4658 | 0.069227 |  |  |  |  |  |  |  |  |  |  |
|  |  | 1000 | -45.564 | 0.67794 | 0.038628 | 2 | AREA_SD pastures | 0.049108 | 5.5465 | 0.0468 | 0.40944 | CONTIG_SD urban infrastructure | 0.063604 | 9.0324 | 0.0974 | 0.53031 |  |  |  |  |  |
|  |  | 2000 | -49.578 | 0.88169 | 0.01419 | 3 | AREA_SD urban infrastructure | 0.042678 | 4.4191 | 0.078 | 0.35583 | CA forest | 0.010669 | 0.78115 | 0.41 | 0.088958 | ENN_SD forest | 0.042113 | 4.3289 | 0.074 | 0.35112 |
